# Proximity labeling puts ZFP36L1 as central hub for posttranscriptional regulation networks in T cells

**DOI:** 10.26508/lsa.202603825

**Published:** 2026-09-09

**Authors:** Anouk P Jurgens, Branka Popović, Floris PJ van Alphen, Maxime Steinmetz, Leyma Wardak, Antonia Bradarić, Sander Engels, Carmen van der Zwaan, Maartje van den Biggelaar, Arie J Hoogendijk, Julien Béthune, Monika C Wolkers

**Affiliations:** 1 Department of Research, T cell Differentiation Lab, Sanquin Blood Supply Foundation , Amsterdam, Netherlands; 2 Department of Research, Bleeding & Hemostasis, Sanquin Blood Supply Foundation , Amsterdam, Netherlands; 3 Landsteiner Laboratory, Amsterdam Institute for Infection & Immunity and Cancer Center Amsterdam, Amsterdam UMC, University of Amsterdam, Amsterdam, Netherlands; 4 Oncode Institute , Utrecht, Netherlands; 5 Department of Biotechnology, HAW Hamburg , Germany

## Abstract

Effective T cell responses against pathogens require a rapid yet tightly controlled remodeling of the proteome, and RNA binding proteins (RBPs) are key in this process. For instance, the RBP ZFP36L1 prevents excessive protein production and thereby limits immunopathology. ZFP36L1 is primarily known to mediate mRNA decay, but it can also regulate other processes. How its mode of action relates to its interaction partners is, however, not well-understood. Here, we mapped the ZFP36L1 interactome in primary human T cells. Using proximity labeling, we identified known and new interactors that regulate 3′UTR-mediated RNA degradation, deadenylation, stress granule/p-body formation, as well as 5′UTR-mediated translation repression and mRNA decapping. Snapshot analysis uncovered the ZFP36L1 interactome dynamics and RNA (in)dependency throughout T cell activation. Intriguingly, proximity labeling also uncovered regulators of ZFP36L1 protein expression. This included the helicase UPF1, which not only interacts with ZFP36L1 protein but that may also promote its protein expression. Altogether, this comprehensive interactome map underlines the versatility of interactions with ZFP36L1 and their possible role in cellular function.

## Abstract (precis)

RNA binding proteins (RBPs) are key regulators of controlling the proteome remodeling during T-cell activation. Using proximity labeling, we present a comprehensive interactome map of the RBP ZFP36L1, demonstrating the versatility of interactions and their possible functioning during T-cell activation.

## Introduction

T cells play a crucial role in clearing infected and malignant cells from our body. To exert their effector function, T cells rapidly remodel their proteome upon antigen recognition (Howden et al, 2019; Wolf et al, 2020; Sinclair & Cantrell, 2025). This includes their ability to produce effector molecules such as granzymes and pro-inflammatory cytokines, a central feature for their capacity to clear target cells (Zhang et al, 2008; Cullen et al, 2010). Yet, tight regulation of gene expression is key for preventing excessive responses, as it can lead to immunopathology (Morgan et al, 2010; Fara et al, 2020). To achieve effective yet balanced immune responses, several regulatory layers are in place, and RNA binding proteins (RBPs) are key contributors herein (Hentze et al, 2018; Turner & DÍaz-Muñoz, 2018; Gebauer et al, 2020; Salerno et al, 2020). Defined by the signals that T cells receive, RBPs control the magnitude and duration of cytokine production (Jeltsch & Heissmeyer, 2016; Behrens et al, 2021; Li et al, 2024), and they regulate cell cycle and checkpoint molecule production levels (Coelho et al, 2017; Petkau et al, 2024; Zandhuis et al, 2025). Notably, mutations in RBPs are linked to autoimmunity in humans (Tavernier et al, 2019; Cuchet-Lourenço et al, 2025 *Preprint*), highlighting the significance of this class of proteins in regulating adequate immune responses.

An important RBP for T cell effector function is ZFP36L1. ZFP36L1 belongs to the ZFP36 protein family and mediates mRNA decay of transcripts that contain AU-rich elements (ARE) in their 3′UTR (Lykke-Andersen & Wagner, 2005), as exemplified for mRNA coding for cell cycle proteins and cytokines (Moore et al, 2018; Cook et al, 2022; Petkau et al, 2022; Popović et al, 2023; Zandhuis et al, 2024). ZFP36L1 deletion in T cells results in increased cytokine production, which in turn can restore function in tumor-infiltrating T cells and improve anti-tumor responses in mice (Popović et al, 2023). Intriguingly, ZFP36L1 regulates cytokine expression upon T-cell activation in a time-dependent manner (Petkau et al, 2022; Popović et al, 2023; Zandhuis et al, 2025): in the first 4–24 h, ZFP36L1 restrains cytokine production, and it does so at least in part by recruiting the CCR4/CNOT complex (Popović et al, 2023). However, ZFP36L1 can also exert several other functions: together with its sister protein ZFP36L2, ZFP36L1 binds to ready-to-deploy cytokine mRNAs in memory T cells, thereby conferring a block of translation and preventing aberrant production in the absence of T cell stimuli (Salerno et al, 2018). Furthermore, ZFP36L1 has been implicated in other cell types in the formation of stress-granules, P-bodies and membraneless structures (Kedersha et al, 2005; Lykke-Andersen & Wagner, 2005; Ma & Mayr, 2018). Provided that most RBPs are highly interactive with RNA and proteins, forming multiple ribonucleoprotein (RNP) (Street et al, 2024) complexes, we hypothesized (1) that ZFP36L1 interacts with a broad set of proteins to carry out diverse functions, and (2) that ZFP36L1 may alter its interactome over the course of T cell activation to modulate its activity. However, to date there is only anecdotal evidence of protein interactions with ZFP36L1 in T cells, and a comprehensive map of the ZFP36L1 interactome throughout T-cell activation is lacking.

To shed light on how ZFP36L1 executes its multiple functions, and which interaction partners it employs to do so, we performed intracellular proximity labeling in human T cells using UltraID, an engineered protein biotin ligase, which rapidly biotinylates proteins within a range of 10 nm (Kubitz et al, 2022). We measured the ZFP36L1 interactome in resting T cells and during T cell activation, and we defined the dependency of RNA for ZFP36L1 interactors. This extensive interactome map uncovered known and previously unreported interactors with ZFP36L1 involved in RNA regulation. In addition, we identified known and novel regulators of ZFP36L1 protein levels. In conclusion, our study yields novel insights into the intricate regulation of gene expression by RBPs, as exemplified here for ZFP36L1.

## Results

### Optimizing proximity labeling in primary human T cells

**Figure 1.**
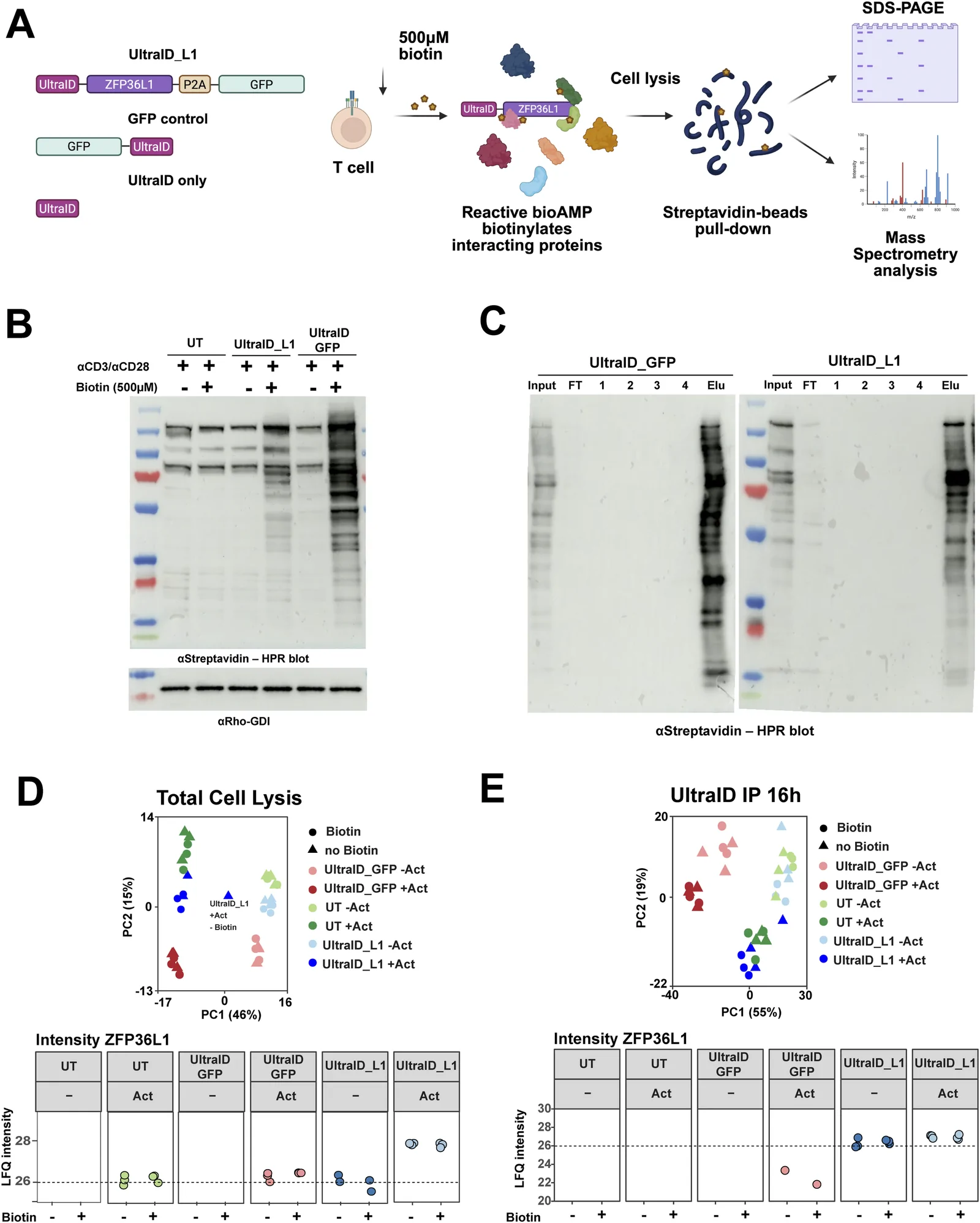
Proximity labeling in primary human T cells. **(A)** Schematic representation of proximity labeling protocol in CD3^+^ T cells and UltraID plasmids used. Indicated UltraID BirA* fusion genes were retrovirally transduced into CD3^+^ T cells (n = 3 donor pools, 40 donors each). After 6 d, GFP^+^ T cells were FACS-sorted on GFP expression. The next day, T cells were re-activated for 16 h with αCD3/αCD28 in the presence or absence of 500 μm biotin. Biotinylated proteins were captured with Streptavidin beads from cell lysates by biotin-streptavidin affinity capture. Biotinylation was confirmed by SDS–PAGE, and biotinylated proteins were identified by Mass Spectrometry. **(B)** Immunoblot for biotinylated proteins of untransduced (UT) T cells, and T cells transduced with UltraID_ZFP36L1_GFP (UltraID_L1) or UltraID_GFP (UltraID_GFP). aRho-GDI was used as loading control. Representative blot of two independently performed experiments, three donor pools each. **(C)** Immunoblot of input (10%), flow through and consecutive washes (1–4) of the pulldown, in addition to the elution (10%). Representative blot of two independently performed experiments, three donor pools each. **(D)** Top: Principal component analysis (PCA) of total cell lysates. Color indicates constructs expressed in T cells, circle/triangle indicates whether or not biotin was added. Bottom: LFQ intensity of ZFP36L1 in the presence or absence of αCD3/αCD28 activation and biotin. Dotted line indicates endogenous ZFP36L1 levels in untransduced activated T cells. n = 3 donor pools of 40 donors each. **(E)** Top: PCA for biotinylated proteins identified by MS upon streptavidin pulldown in T cell screen 1 of indicated conditions. Bottom: LFQ intensity of ZFP36L1 in the presence or absence of αCD3/αCD28 activation and biotin. Dotted line indicates endogenous ZFP36L1 levels in untransduced activated T cells. n = 3 donor pools of 40 donors each. See Fig S1. Source data are available for this figure.

**Figure S1.**
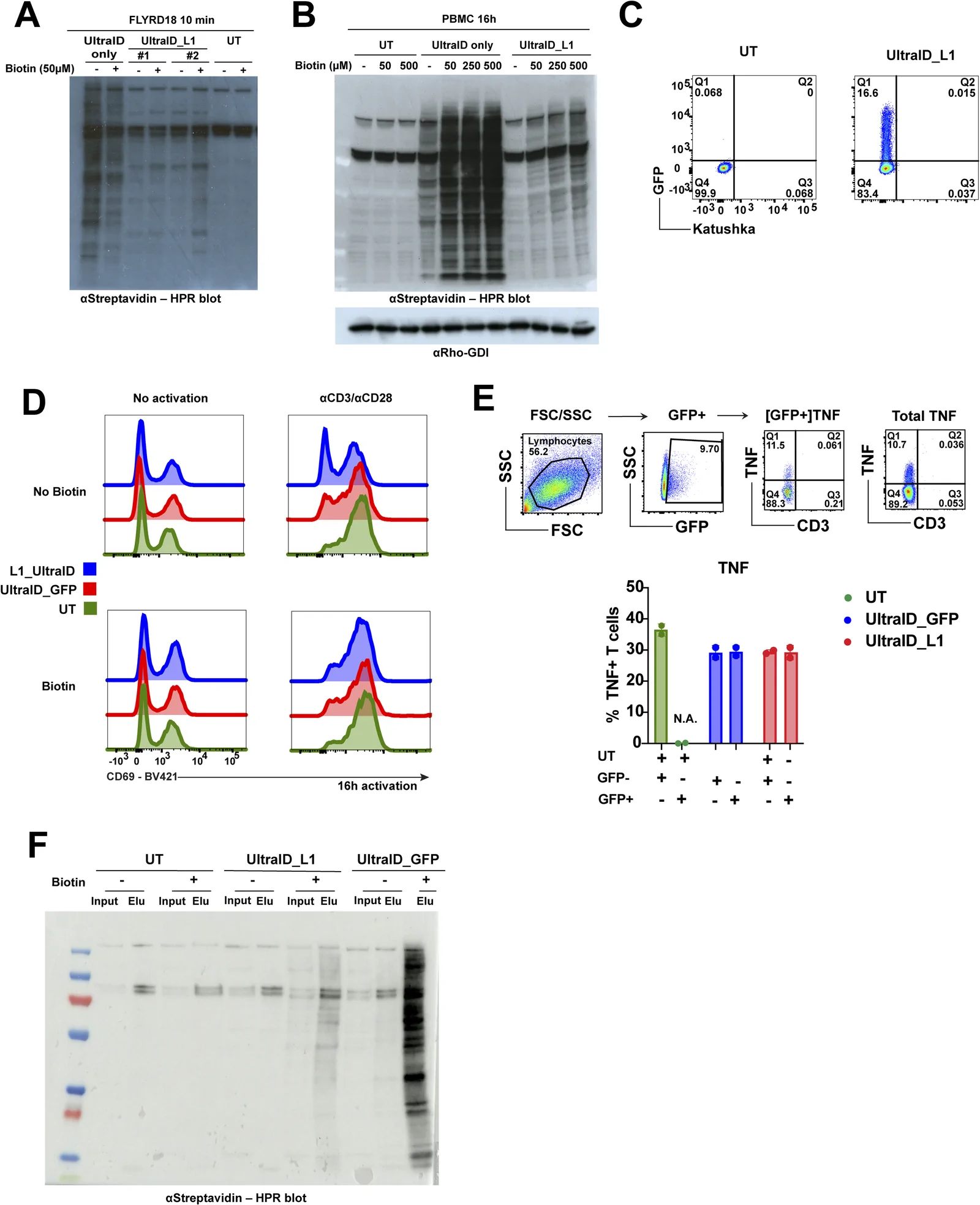
Optimizing Proximity labeling in primary human T cells. **(A)** Immunoblot of biotinylated proteins with aStreptavidin-HPR of FLYRD18 cells left untransduced (UT) or transduced with indicated constructs (UltraID_L1 with two different DNA preparations and UltraID_only) in the presence or absence of ±50 μm biotin for 10 min. Representative of two independently performed experiments. **(B)** Immunoblot of T cells transduced with UT, UltraID_L1 or UltraID_only in the presence or absence of αCD3/αCD28 and with indicated concentrations of Biotin for 16 h. Rho-GDI was used as loading control. Representative of at six donors in two independently performed experiments. **(C)** Flow cytometry plot of transduction efficiency based on GFP expression. **(D)** CD69 expression of untransduced (UT), UltraID_L1 (Blue) or UltraID_GFP (Red) T cells that were rested for 7 days, or that were re-activated with αCD3/αCD28 for 16 h in the presence or absence of 500 μm Biotin. Representative of at least three donors. **(E)** Untransduced (UT Green), UltraID_GFP (Red), and UltraID_L1 (Blue) transduced T cells were re-activated with αCD3/αCD28 for 5 h. Top panels: gating strategy. Bottom panel: percentage of TNF-expressing T cells. n = 3 donors. UT, untransduced T cells. GFP expression based on second left flow cytometry panel (GFP+ and GFP−). **(F)** Enrichment of biotinylated proteins as visualized by Streptavidin blot of 10% input and 10% of the final elution of T cells transduced with UltraID_L1 or UltraID_GFP, and that were re-activated with αCD3/αCD28 for 16 h in the presence of biotin. Representative of at least two independent experiments with three donor pools. See Fig 1. Source data are available for this figure.

To perform proximity labeling for ZFP36L1, we fused the biotin ligase variant UltraID (Kubitz et al, 2022) to the N-terminus of ZFP36L1 (UltraID_L1). To identify UltraID_L1 expressing cells, we co-expressed a GFP reporter gene with ZFP36L1 (UltraID_L1_P2A_GFP; Fig 1A). UltraID fused to the C-terminus of GFP (UltraID_GFP) and UltraID alone (UltraID only) served as controls (Fig 1A). As previously reported (Kubitz et al, 2022), clear biotinylation profiles were detected in the human fibrosarcoma cell line FLYRD18 after 10 min of incubation with 50 μm biotin (Fig S1A). This was, however, not the case in human T cells. T cells require biotin-dependent carboxylases for fatty acid synthesis and amino acid metabolism, and proliferating T cells absorb five-fold more biotin than resting T cells (Zempleni & Mock, 1998, 1999, 2000). Therefore, increasing the biotin levels to up to 500 μM was required to obtain detectable biotinylation profiles in human T cells (Fig S1B). Moreover, as 10 min appeared not sufficient to attain proximity labeling in human T cells, we increased the incubation time to 16 h in this optimization step, when T-cell activation plateaus, therefore requiring less biotin (Fig S1B, second & third lane: UltraID_only & UltraID_L1). Proximity labeling with UltraID_GFP displayed more specific biotinylated protein bands than UltraID protein alone (Figs 1B and S1B), possibly allowing the latter to freely diffuse and amply interact with proteins in the cytoplasm. In addition to, because GFP is of similar size as ZFP36L1, we considered UltraID_GFP as control more appropriate and used it in our analyses.

### Proximity labeling identifies the ZFP36L1 interactome in primary human T cells

Having established proximity labeling in human T cells, we questioned which proteins interact with ZFP36L1 upon T cell activation. To avoid alterations in T cell function by exogenous ZFL36L1 expression, we aimed for limited (∼20%) transduction efficiency (Fig S1C). This expression level did not influence T cell viability, the expression levels of the activation marker CD69, or the production of TNF, a reported target of ZFP36L1 (Kontoyiannis et al, 1999; Popović et al, 2023; Fig S1D and E). Furthermore, to limit inter-donor variability, we used 3 T cell pools of 40 healthy donors each. FACS-sorted UltraID_L1 and UltraID_GFP expressing T cells were expanded for 7 days and then activated for 16 h with αCD3/αCD28 in the presence or absence of biotin, a timeframe when ZFP36L1 displays its highest activity in T cells (Zandhuis et al, 2025). Immunoblots revealed distinct biotinylation patterns between UltraID_GFP and UltraID_L1 expressing T cells (Figs 1C and S1F), indicating differential protein interactions.

**Figure S2.**
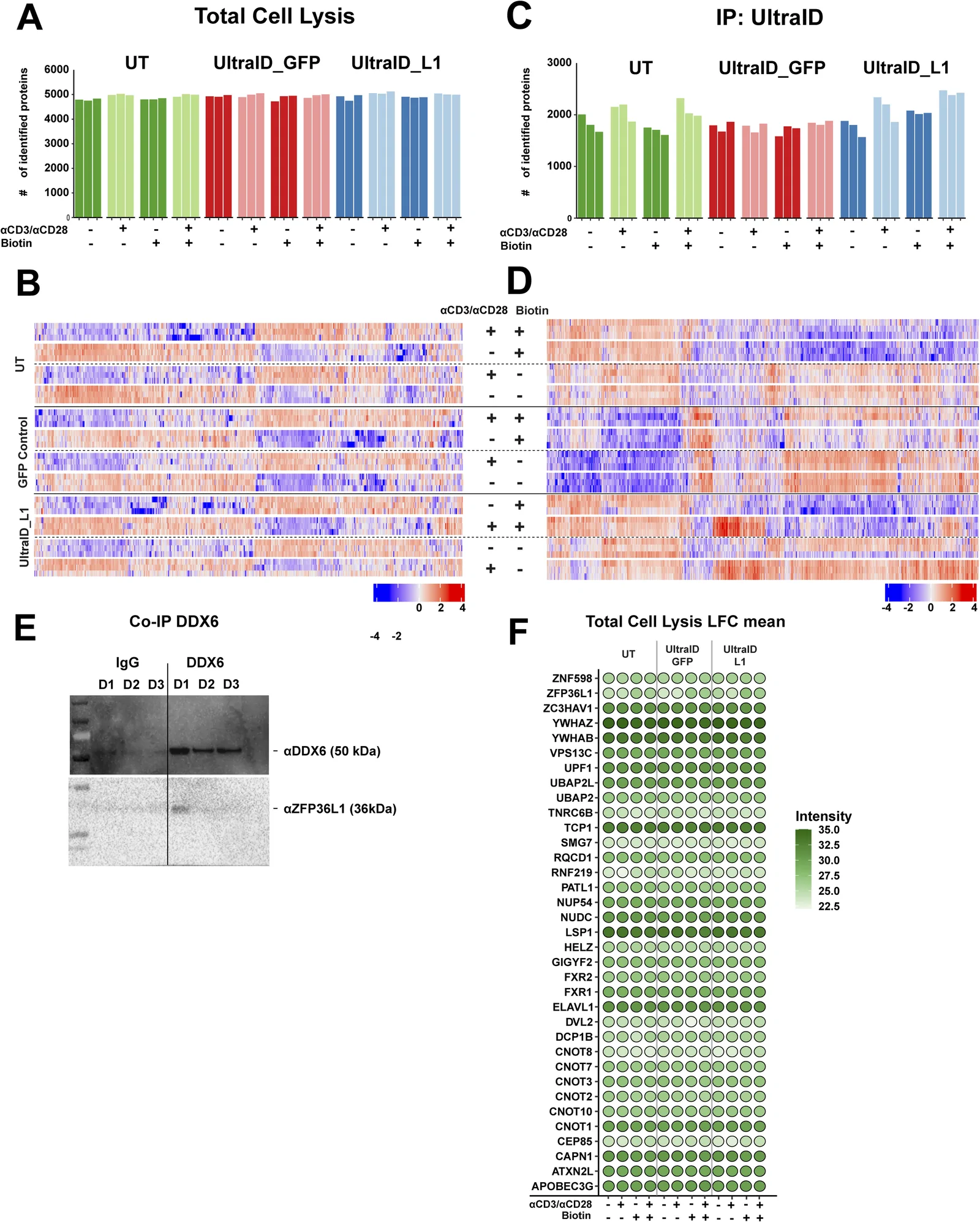
Proximity labeling identifies the ZFP36L1 interactome in primary human T cells. **(A)** Number of identified proteins of the total Cell Lysis (TCL) by Mass Spectrometry (MS). Each bar represents one donor pool of 40 donors. **(B)** Heatmap of all proteins that were significantly enriched in one of the 12 conditions in Total Cell Lysate (5250 proteins, LFC >1, *P*.adj <0.05). Color scale represents Z-scored log2 median-centered averaged intensities. **(C)** Number of identified proteins after UltraID_L1 streptavidin pulldown (IP) by MS. Each bar represents one donor pool of 40 donors. **(D)** Heatmap of proteins that were significantly enriched in the UltraID-L1 IP: T cell screen 1 (2,439 proteins, LFC >1, *P*.adj <0.05). Color scale represents Z-scored log2 median-centered averaged intensities. **(E)** Immunoblot analysis of co-immunoprecipitation with αDDX6 or IgG control from lysates of resting T cells. Lysates were immunoblotted for DDX6 and ZFP36L1.n = 3 donors. **(F)** LFC mean intensities of protein expression in the total cell lysates that were found to interact with ZFP36L1 as depicted in Fig 2B. Proteins with LFC >1, *P*. adj <0.05 are considered significantly enriched. Scale represents LFC average intensities. Mean of 3 donor pools. See Fig 2.

Mass spectrometry (MS) analysis of the total cell lysates identified in total 5,250 proteins (range 4,500–5,000 proteins/sample; Fig S2A and Table S1). Principal component analysis (PCA) confirmed that biotinylation per se did not affect the total proteome (Fig 1D). Rather, T-cell activation was the prime driver of differential protein expression (Figs 1D and S2B). As expected, differentially expressed proteins between resting and activated T cells included ZFP36L1, which was induced upon T cell activation (Fig 1D). MS analysis of isolated biotinylated proteins identified a total of 2,439 proteins (range: 1,700–2,500 proteins/sample; Fig S2C and Table S1). As expected from its fusion to the proximity labeling enzyme, ZFP36L1 was primarily detected in the UltraID_L1 pull down (Fig 1E). Again, PCA was mainly driven by T-cell activation status, together with the expression of UltraID_GFP versus UltraID_L1 (Figs 1E and S2D).

Table S1. MS analysis of total cell lysate and UltraID proximity labeling for ZFP36L1 in resting T cells and after 16 h of activation.

**Figure 2.**
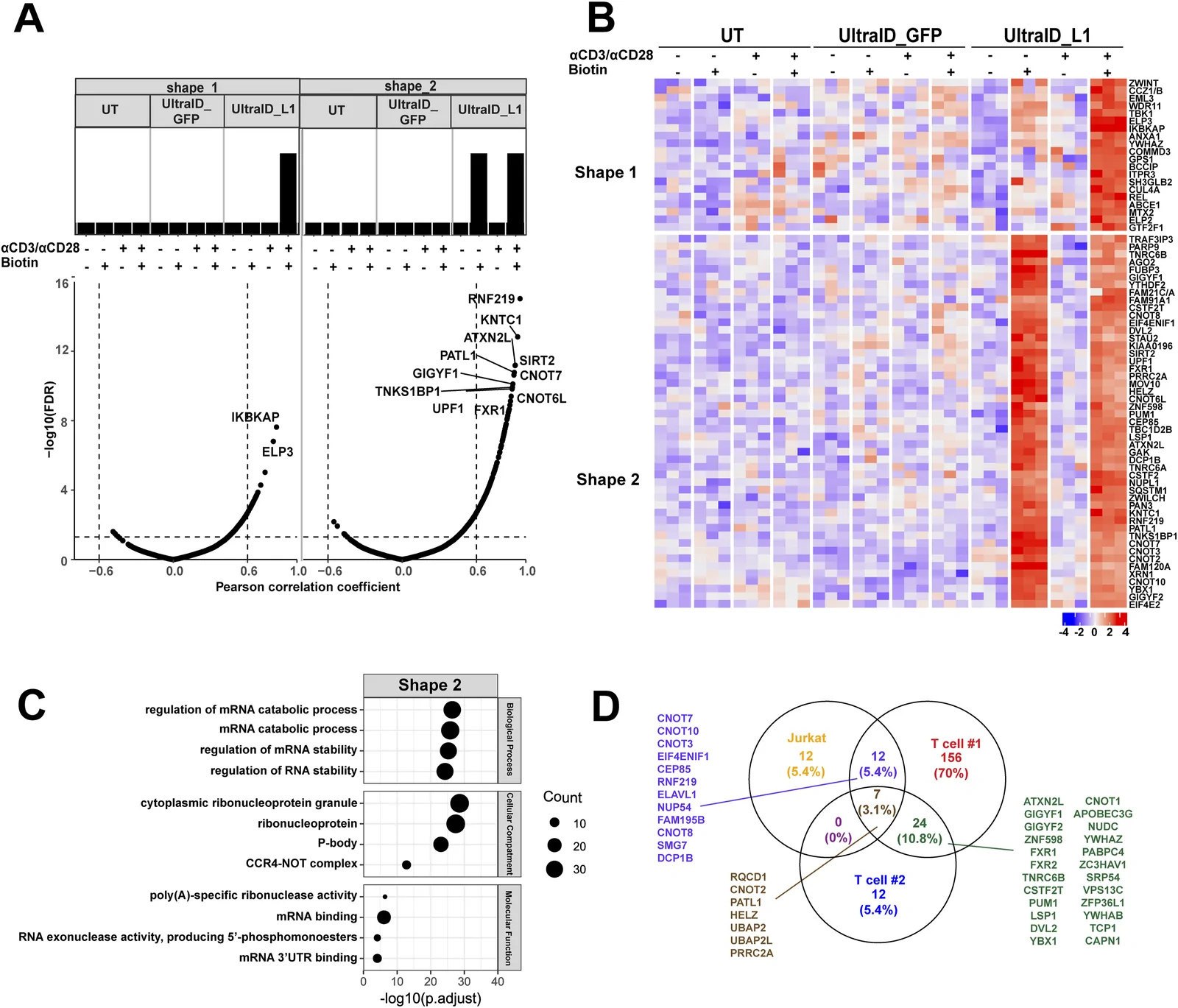
Proximity labeling identifies the ZFP36L1 interactome in primary human T cells. **(A)** Supervised classification of biotinylated proteins identified as ZFP36L1 interactors. Top panel indicates which sample(s) were set to an arbitrary high. Shape 1: biotinylated proteins enriched (LFC >1, *P*.adj <0.05) in activated T cells expressing UltraID_L1 compared with all other conditions. Shape 2: biotinylated proteins enriched in T cells expressing UltraID_L1 irrespective of T cell activation status. Correlation between hits and disorder-specific theoretical protein profiles using a Pearson correlation coefficient of >0.6 (dotted line) and Benjamini-Hochberg adjusted *P*-value (*P*.adj) <0.05 was used as threshold. **(B)** Heatmap of supervised classification displaying biotinylated proteins identified in A. Color scale represents Z-scored log2 median-centered averaged intensities. n = 3 donor pools of 40 donors each. **(C)** GO analysis of identified ZFP36L1 interactors from shape 2. Protein interactors were devided according to biological process, cellular component and molecular function. **(D)** Venn diagram depicting the overlap of identified ZFP36L1 interactors (Pearson correlation coefficient >0.5) from shape 1 and 2 for Jurkat cells and two independently performed pulldowns in primary T cells (T cell screen 1 and 2). See Figs S2 and S3.

To uncover the ZFP36L1 interactome, we utilized supervised classification (Kreft et al, 2023). In short, we generated theoretical profiles in which the conditions of interest (interacting with ZFP36L1 in resting T cells [shape 1] or in resting and activated T cells [shape 2]) were set to an arbitrary high and low in all other samples, after which differentially expressed proteins in the “high” sample were correlated with all statistically significant proteins (*for more details see Methods and Materials section*) (Fig 2A). Using a cut-off of log fold change (LFC) >1, adjusted *P*-value (*P*-aof bdj) <0.05, and Pearson correlation ≥0.6, we identified 156 proteins in close proximity to ZFP36L1. 27 ZFP36L1 interactions depended on the presence of UltraID_L1 and were found with T-cell activation only (shape 1), and 129 irrespective of the T cell activation status (shape 2; Figs 2A and B, S2E, and Table S1). As expected, gene ontology (GO) analysis from shape 2 identified RNA-related processes, such as mRNA decay, RNP granules, and mRNA binding (Fig 2C). Identified proteins included previously reported ZFP36L1 interactors, such as the deadenylating CCR4-CNOT complex members CNOT1, CNOT2, CNOT3, CNOT7, CNOT6L, CNOT10 and CNOT11 (Fabian et al, 2013; Ito-Kureha et al, 2020; Otsuka et al, 2020; Akiyama & Yamamoto, 2021; Fig 2B and C). Other known ZFP36L1 interactors were the 14-3-3 protein family members YWHAB and YWHAZ (Bustin & Mckay, 1999; Johnson et al, 2002) and the exocyst complex components EXOC1, EXOC3, EXOC4 (Popović et al, 2023; Fig 2B).

**Figure S3.**
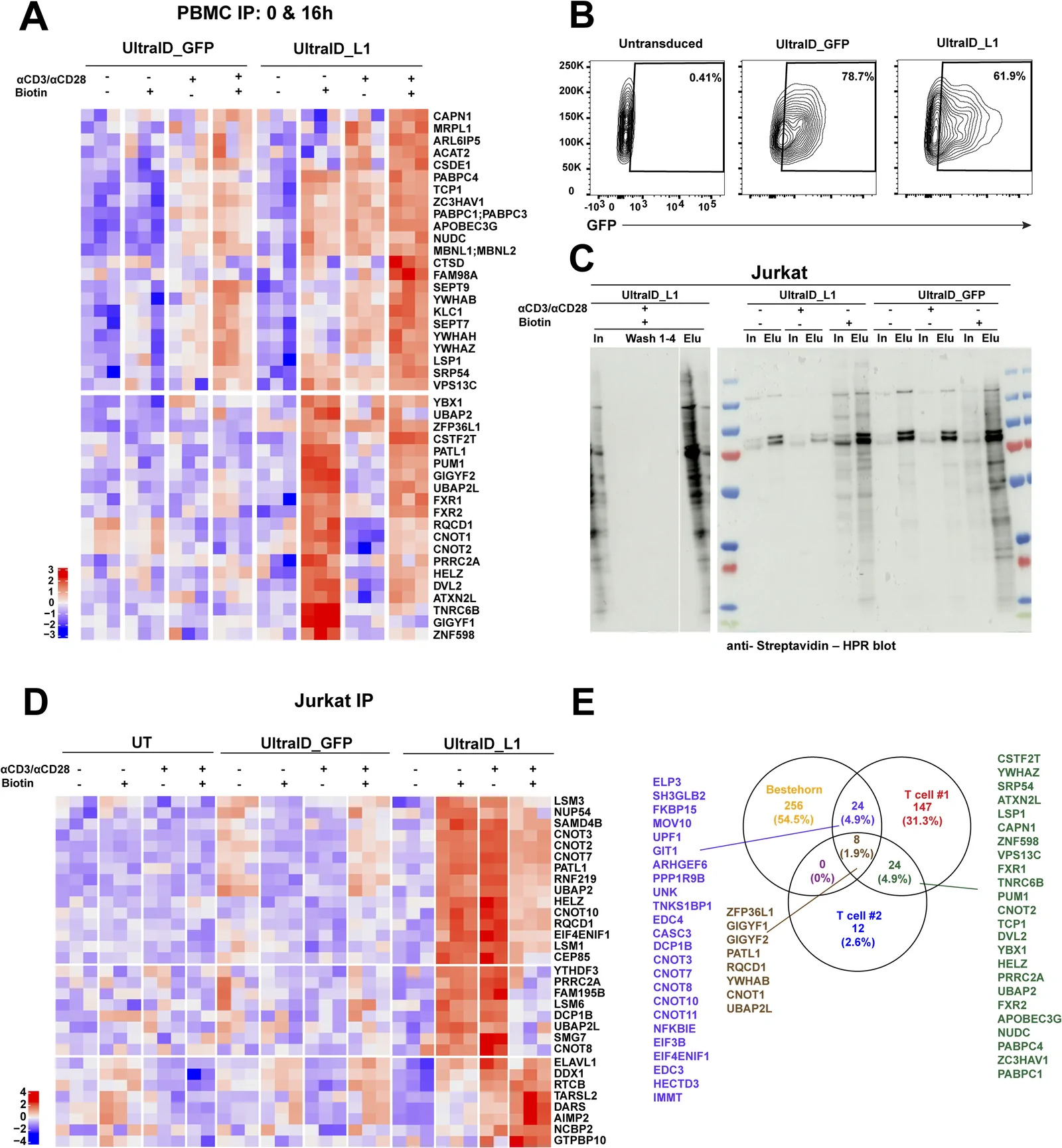
Proximity labeling identifies the ZFP36L1 interactome in Jurkat cells. **(A)** Repeat experiment of UltraID Pull down for ZFP36L1 (T cell screen 2). Heatmap of supervised classification displaying biotinylated proteins that were significantly enriched. Correlation between hits and disorder-specific theoretical protein profiles had a Pearson correlation of >0.5, *P*.adj <0.05, LFC >1. Color scale represents Z-scored log2 median-centered averaged intensities. n = 3 donor pools of 40 donors each. **(B)** Flow cytometry analysis of GFP-expressing Jurkat cells transduced with indicated constructs. Representative of three technical replicates. **(C)** Streptavidin immunoblot of input (10%), flow through and consecutive washes (1–4) of the pulldown, in addition to the elution (10%). Representative of at least two independent experiments with three technical replicates. **(D)** Heatmap of supervised classification displaying biotinylated proteins that were enriched in Jurkat UltraID_L1 16 h screen. Correlation between hits and disorder-specific theoretical protein profiles had a Pearson correlation of >0.5 and *P*.adj <0.05 and LFC >1. Color scale represents Z-scored log2 median-centered averaged intensities. n = 3 biological replicates. **(E)** Venn diagram of ZFP36L1 protein interactions from the UltraID T cell screen 1 and 2 identified in Fig 2 and Fig S3, compared with the ZFP36L1 interactome screen in HEK 293T cells from Bestehorn et al (2025). See Fig 2. Source data are available for this figure.

Repeating the UltraID proximity labeling in primary human T cells confirmed 31 proteins in close proximity to ZFP36L1. Thus, even with the lower coverage of the second screen, UltraID proximity labeling provides robust outcomes (Figs 2D, S3A, and Table S2). Furthermore, an UltraID-L1 pulldown in Jurkat cells yielded similar - yet not identical - proteins in proximity to ZFP36L1 (Figs 2D, S3B–D, and Table S2), highlighting the added value of performing proximity labeling in primary cells. Similarly, 32 proteins (6.8%) found in either one of both T cell screens overlapped with a recently published ZFP36L1-APEX proximity labelling in HEK293T cell line, and only eight proteins were shared between the two T cell screens and HEK293T cells (Bestehorn et al, 2025). Yet, 256 and 183 proteins were specifically enriched in HEK293T cells or T cells, respectively (Fig S3E), suggesting that either expression of certain proteins is cell-type specific, or ZFP36L1 has cell-type specific protein interactions, in addition to its core interactome. Combined, we here showcased the potential of UltraID proximity labeling in primary human T cells to measure protein–protein interactions, as exemplified for ZFP36L1.

Table S2. Repeat of the UltraID proximity labeling for ZFP36L1 in primary human T cells and Jurkat cells.

### ZFP36L1 interactome covers a multitude of RNA-related processes as well as protein turnover

**Figure 3.**
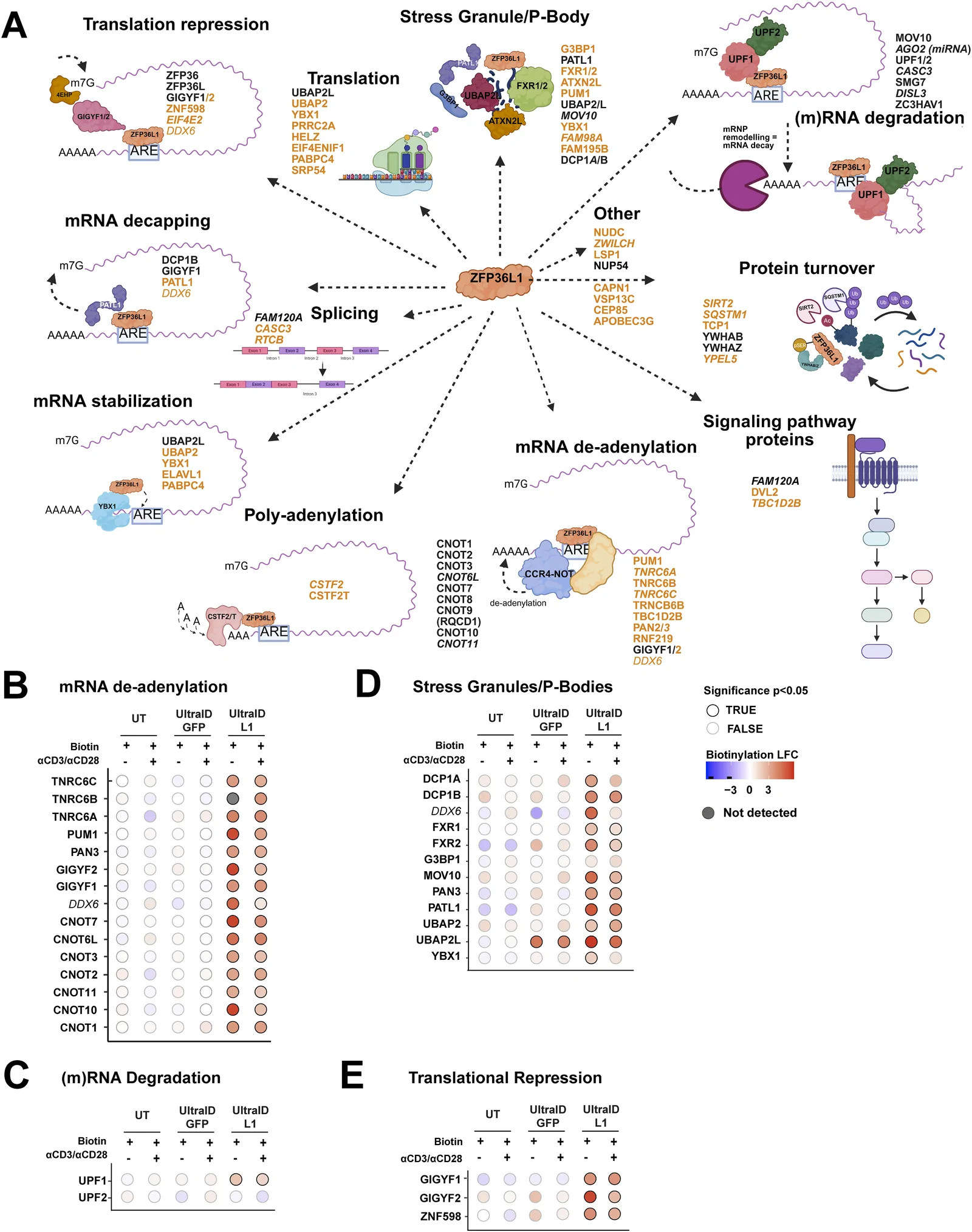
ZFP36L1 interactome correlates with a multitude of RNA control mechanisms. **(A)** Network of the ZFP36L1 interactome identified in at least 1/2 proximity labeling T cell screens and/or the Jurkat screen. Cut off for T cell screen 1 was Pearson correlation of 0.6, and for T cell screen 2 and Jurkat screen 0.5, and LFC >1 and *P*.adj <0.05 for all three. Proteins annotated according to molecular function or cellular location. Black: previously reported interactions, Orange: newly identified proteins interacting with ZFP36L1. Italic: Identified in only one proximity labeling T cell screen or in the Jurkat screen. Non-bold: protein part of a complex of identified proteins, but below the cut-off. *Created in BioRender. Jurgens, A*. (2026). **(B, C, D, E)** LFQ intensity plots of identified ZFP36L1 interactors. Black circles: significantly enriched proteins (*P*.adj <0.05) in Biotin over no Biotin samples. Grey circles: no significant enrichment. Fold-change (log2FC) enrichment is indicated by color code. Mean of 3 donor pools of 40 donors each.

We next zoomed in on the cellular processes annotated for known and candidate interactors of ZFP36L1. Specifically, we examined proteins that were either found in close proximity to ZFP36L1 in at least two out of three screens, or that showed strong correlation with ZFP36L1 proximity in our “shapes” in T cells screen 1 and that were part of a complex with another candidate interactor. As expected, most identified proteins contribute to mRNA decay processes such as mRNA stabilization, de-adenylation, de-capping and degradation (Fig 3A). In addition to the above-mentioned CCR4-NOT complex proteins (Fig 3A and B), we identified novel interactions from the de-adenylation complex. This included the PAN2/3 complex proteins (Fig 3A), which cooperate with the CCR4-Not complex in facilitating poly(A) tail shortening, a rate limiting step of mRNA degradation (Yamashita et al, 2005; Scheller et al, 2007). We also detected PUM1, which directly binds to the CCR4-NOT complex and supports its function in mRNA de-adenylation (Enwerem et al, 2021) and TNRC6C, which directly associates with the PAN2/3 and CCR4-NOT complexes (Braun et al, 2011), in addition to its contribution to microRNA degradation (Fig 3A). These ubiquitous interactions of ZFP36L1 with most - if not all - proteins involved in mRNA de-adenlyation pathway thus evidence ZFP36L1’s central place in this process. Of note, and in line with previous reports of the cell-cycle dependent nuclear localization of ZFP36L1 (Matsuura et al, 2020; Bestehorn et al, 2025), we detected interactions with the nuclear alternative poly-adenylation regulating proteins CSTF2 and CSTF2T (Chuvpilo et al, 1999) and with Fam120A, which associates with the splicing protein SRFS1 (Cho et al, 2023; Fig 3A).

In the mRNA degradation pathway, we identified the RNA helicase UPF1 as being in close proximity to ZFP36L1 (Fig 3A). UPF1 regulates nonsense-mediated mRNA decay (NMD), Staufen-mediated mRNA decay (SMD), and Regnase-1-mediated mRNA decay (Fiorini et al, 2015, 2018; Iwai et al, 2024; Musaev et al, 2024; Fig 3C). ZFP36L1 also interacted with MOV10 (Fig 3A), a 5′ to 3′ RNA helicase that supports UPF1 in target mRNA degradation (Gregersen et al, 2014). Intriguingly, UPF1 deficiency in early murine B cell development resembles the phenotype of ZFP36L1/ZFP36L2 deficiency, which leads to perturbed thymic and B cell development in mice (Hodson et al, 2010; Iwai et al, 2024). UPF1 and MOV10 interactions with ZFP36, the sister protein of ZFP36L1, were recently reported in HEK293T cells (Bestehorn et al, 2025; Fig S2E), indicating that interactions with UPF1 and MOV10 are conserved in different cell types and between ZFP36 family members. This also holds true for the poly(A) RNase PARN and the RISC complex member AGO2 (Fig 3A), confirming that ZFP36 proteins play a central role in regulating mRNA stability of their target genes.

In line with the described location of ZFP36L1 in stress granules (SG) and P-bodies (Kedersha et al, 2005), ZFP36L1 interacted with the SG and P-body associated proteins PATL1, UBAP2L, and MOV10 together with G3BP1 (formation of stress granules) and possibly with DDX6 (formation of p-bodies) (Teixeira et al, 2005; Bounedjah et al, 2014; Ayache et al, 2015; Tourrière et al, 2023), with the latter only reaching a *P*-value of 0.06 (Fig 3A, B, and D). A DDX6 Co-IP under native conditions to confirm interaction with ZFP36L1 was inconclusive, indicating that these interactions are weak or short-lived, if present (Fig S2E). UltraID proximity labeling extended this known list of SG and P-body associated ZFP36L1 candidate interactors to FXR1/2, ATXN2L, PUM1, UBAP2, and YBX1 (Fenger-Grøn et al, 2005; Scheller et al, 2007; Curdy et al, 2023).

We also identified proteins in close proximity to ZFP36L1 that are involved in 5′-UTR mediated RNA processes. This included the decapping proteins DCP2 and DCP1B (Chen et al, 2024) and PATL1, one of the strongest ZFP36L1 candidate interactors (Figs 3A and D and S2F), which also interacts with DCP1 (Lykke-Andersen & Wagner, 2005; Scheller et al, 2007). DDX6 could also possibly contribute to this 5′UTR interaction, as it can bind to the EDC complex to assist mRNA decapping by DCP1/2 and PATL1 (Sharif et al, 2013). Finally, the RBP scaffolding proteins GIGYF1, GIGYF2 and ZNF598, were consistently identified in close proximity to ZFP36L1 (Fig 3A and E). GIGYF1 and GIGYF2 interact with DDX6 and with the translation repressor 4EHP (Tollenaere et al, 2019; Weber et al, 2020), both of which were found interacting with ZFP36L1 (Figs 3A and 2B). Interestingly, ZFP36L1 also interacts with factors that can promote translation, such as UBAP2 and UBAP2L proteins (Luo et al, 2020), as well as the m6A reader PPRC2A, which is reported to regulate mRNA metabolism and facilitate translation of uORF-containing transcripts by promoting leaky scanning (Bohlen et al, 2023; Fig 3A).

In addition to RBP-ZFP36L1 interactions, we detected interactions between ZFP36L1 and the phosphoserine-containing protein binders YWHAB/Z (Fig 3A). These 14-3-3 proteins were previously described to regulate the protein turnover of ZFP36L1 (Stoecklin et al, 2004; Benjamin et al, 2006). In addition, ZFP36L1 interacted with the protein quality control protein SQSTM1 (p62), which relocates ubiquitinylated proteins to the lysosome for degradation (Fig 3A). Lastly, ZFP36L1 interacts with SIRT2 (Fig 3A), which de-acetylates lysines and thereby alters protein expression and function (Knyphausen et al, 2016). In conclusion, UltraID proximity labeling uncovered a multitude of ZFP36L1 interactions with proteins that contribute to a broad range of regulatory pathways, each defining different aspects of regulating the fate of target mRNA and protein expression.

### Time-dependent protein-protein interactions with ZFP36L1

**Figure 4.**
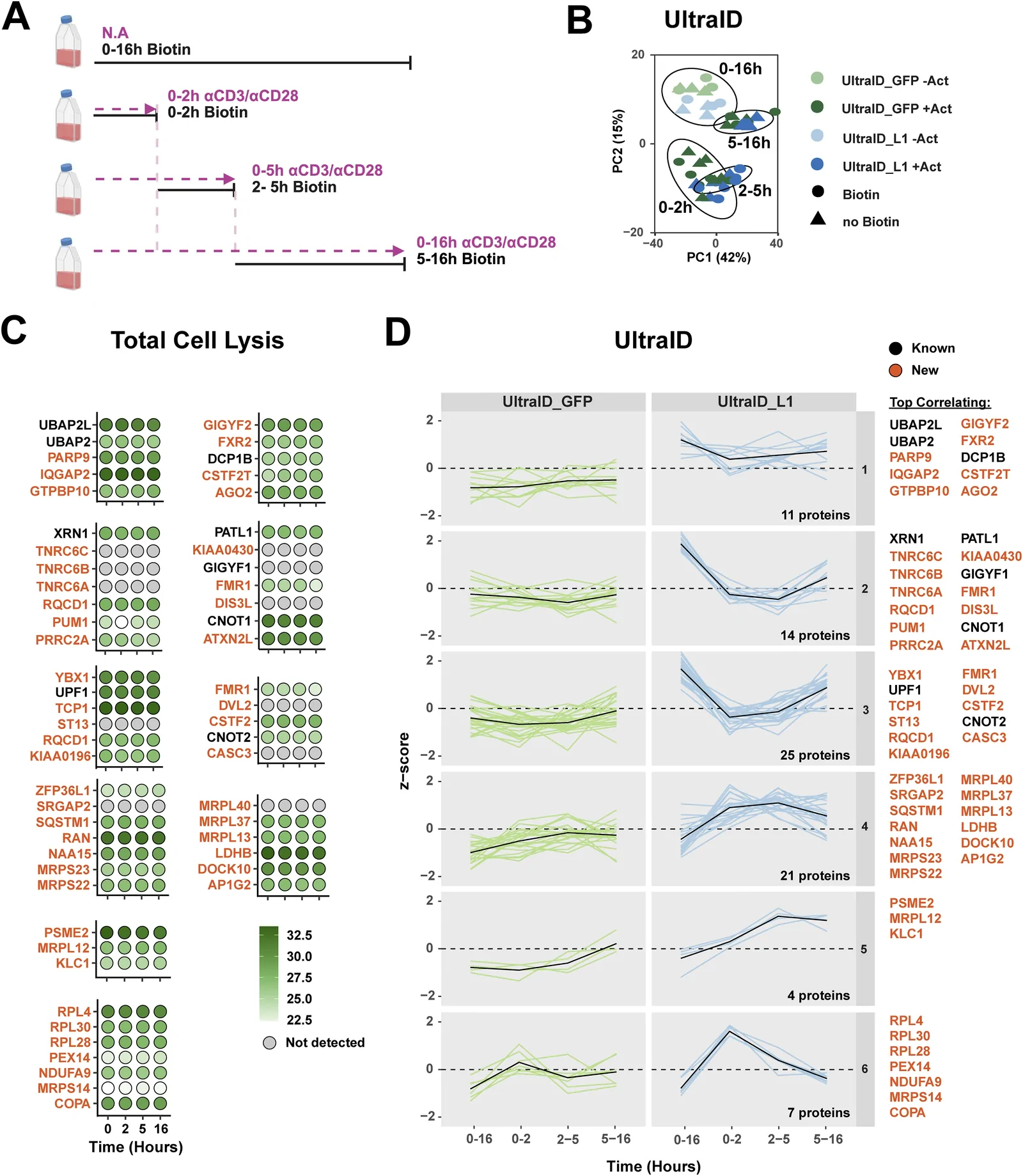
Time-resolved proximity labeling reveals dynamic interactions with ZFP36L1 during T cell activation. **(A)** Graphical representation of UltraID time course experiment. T cells were re-activated with αCD3/αCD28 for indicated time points (magenta dotted arrow) in the presence or absence of biotin. Biotin was added at indicated time points (black line). **(B)** PCA for indicated conditions. Outlined circles indicate different timepoints of T cell activation. Color indicates UltraID_L1 (Blue) and UltraID_GFP (Green). Circle indicates the addition of biotin, triangle: no biotin added. n = 3 donor pools of 40 donors each. **(C)** Protein abundance in full cell lysates of proteins that were significantly enriched for biotinylation (LFC >1, *P*. adj <0.05) in the time course of UltraID_L1 samples (panel D). Grey indicates not detected. Fold-change (log2FC) enrichment is indicated by color code. Mean of 3 donor pools of 40 donors each. **(D)** Scaled intensity profiles of UltraID_L1-enriched proteins across activation time points, as determined by supervised classification of six shapes (Fig S4B) identified to contain interaction partners with a Pearson correlation of >0.5, and *P*. adj <0.05 and LFC >1. Mean of 3 donor pools of 40 donors each. See Fig S4.

**Figure S4.**
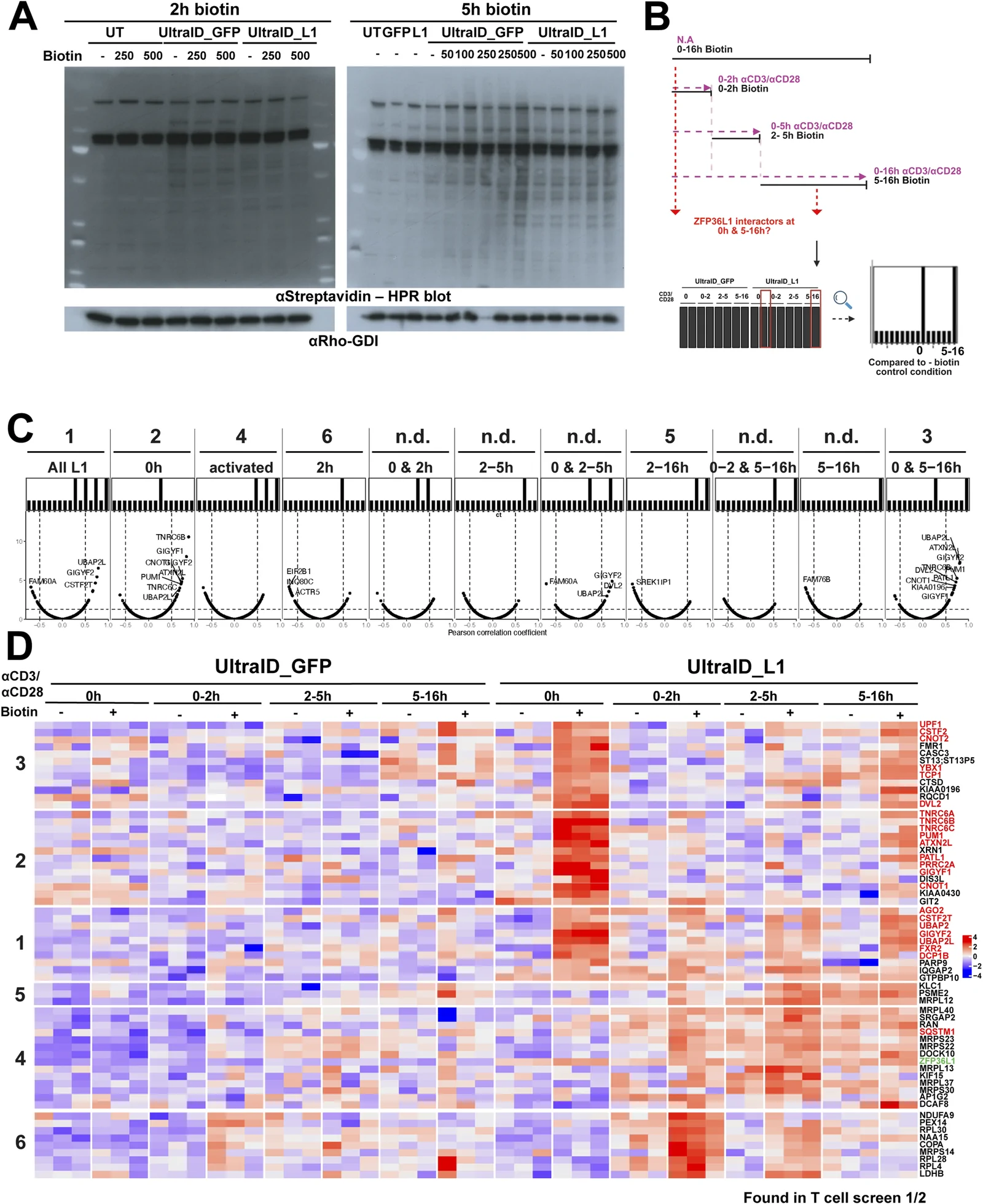
Time-resolved proximity labeling reveals dynamic interactions with ZFP36L1. **(A)** Immunoblot of biotinylated proteins of indicated T cells after 2 and 5 h of activation with αCD3/αCD28. Indicated concentration of biotin was added during the entire time of T cell activation. Rho-GDI served as loading control. Blot representative of three independent donors. **(B)** Graphical explanation of analysis pipeline resulting in shape analysis in C. Comparisons include the information on all time points on protein identification between + biotin and – biotin. *Created in BioRender. Jurgens, A. (2026)* https://BioRender.com/hgz8nye. **(C)** Shape analysis of biotinylated proteins from the T cell activation time course (Fig 5B) using supervised classification, identified to contain interaction partners with a Pearson correlation of >0.5, and *P*.adj <0.05 and LFC >1. Mean of 3 donor pools of 40 donors each. **(D)** Heatmap of supervised classification of biotinylated proteins enriched in the shape analysis from panel B. Proteins marked in red were also identified in T cell screen 1 and/or 2. Color scale represents Z-scored log2 median-centered averaged intensities. Mean of 3 donor pools of 40 donors each. See Fig 4. Source data are available for this figure.

Having identified a broad scala of proteins in close proximity with ZFP36L1 (Fig 3), we next questioned whether these interactions were constant, or whether they were subject to change over the course of T-cell activation. To answer this question, we performed a time-lapse proximity labeling, by activating T cells for 2, 5, and 16 h, and by adding biotin between these timepoints for 2 h, for 3 h (from 2–5 h of activation) or for 9 h (from 5–16 h of activation) (Figs 4A and S4A). These time points were chosen based on the expression pattern of ZFP36L1 in T cells: ZFP36L1 is rapidly induced upon T-cell activation, starting at 2 h post activation, peaking at 5 h of T-cell activation and its expression declines again at the later phase of T cell activation, i.e., at 16 h of T cell activation (Petkau et al, 2022; Zandhuis et al, 2025). Additionally, these expression kinetics are concomitant with the changes of interacting with target mRNAs (Petkau et al, 2022; Popović et al, 2023; Zandhuis et al, 2025).

PCA of biotinylated proteins revealed that the time of T-cell activation rather than the biotinylation time was the prime driver of differential protein abundances, and in addition that the UltraID_L1 pulldowns slightly clustered away from UltraID_GFP control pulldowns (Fig 4B and Table S3). Supervised classification of binding kinetics of proteins in proximity to ZFP36L1 (LFC >1, adjusted *P* < 0.05, Pearson correlation ≥0.5) resulted in 11 shapes, of which 6 shapes contained enriched proteins (Fig S4B and C and Table S3). MS analysis of total cell lysates revealed limited changes throughout T-cell activation of protein abundance of ZFP36L1 interactors, thereby excluding the possibility that alterations of interactions are a mere consequence of differential protein levels (Fig 4C and Table S3).

Table S3. Time-resolved MS analysis of total cell lysate and proximity labeling for ZFP36L1.

Many proteins in proximity to ZFP36L1 were already detected in resting T cells (0 h; Fig S4C and D), which points to robust, activation-independent interactions with ZFP36L1. Alternatively, some interactions may also result from the exogenous ZFP36L1 expression in UltraID_L1-containing T cells, which is slightly higher than that of endogenous ZFP36L1 (Fig 1D). Nevertheless, we could clearly identify differential protein interaction profiles with ZFP36L1 throughout the 16 h time course of T-cell activation. Some proteins such as the SG/PB-associated proteins UBAP2 and UBAP2L, the decapping protein DCP1B, as well as GIGYF2 and CSTF2T interacted with ZFP36L1 throughout T-cell activation (shape 1; Fig 4D). Other proteins reduced their interaction with ZFP36L1 at 2 h before they gradually increased their interactions again (shapes 2 + 3, Fig 4D). This class included proteins involved in mRNA degradation, and translation repression, such as including UPF1, GIGYF1, and CSTF2, but also the deadenylation proteins TNRC6A-C and the SG/PB-associated proteins ATXN2L, PUM1, PATL1, and YBX1 (Fig 4D).

We also identified proteins that increased their interaction with ZFP36L1 upon T-cell activation (shapes 4 + 5; Fig 4D). Intriguingly, the majority of these were mitochondrial small and large ribosomal proteins that are translated in the cytoplasm, after which they are transported to the mitochondria where they assemble into complexes (Christian & Spremulli, 2012) (MRPS & MRPL proteins, shape 4, Fig 4D). T-cell activation triggers mitochondrial biogenesis, which is essential for translating cytotoxic molecules and for maintaining sustained cytotoxicity (Scharping et al, 2016; Lisci et al, 2021; Lisci & Griffiths, 2023). Because the total protein levels of mitochondrial proteins did not change upon T-cell activation (Fig 4C and D and Table S3), ZFP36L1 may thus potentially regulate their localization and/or transport of translated proteins.

Lastly, we identified proteins that only briefly increased their interaction with ZFP36L1 at 2 h of activation (shape 6, Fig 4D). These included the ribosomal proteins RPL4, RPL28, and RPL30, and the mitochondrial proteins MRPS14 and NDUFA9, the latter being a component of Complex I of the respiratory chain (Baertling et al, 2018; Fig 4D). In conclusion, ZFP36L1 displays both constant and time-dependent interactions, and these altered interactions belong to different RNA regulatory pathways, which could define different emphasis of ZFP36L1 on its divergent PTR functions over the course of T cell activation.

### RNA-dependent and -independent interactions with ZFP36L1

**Figure S5.**
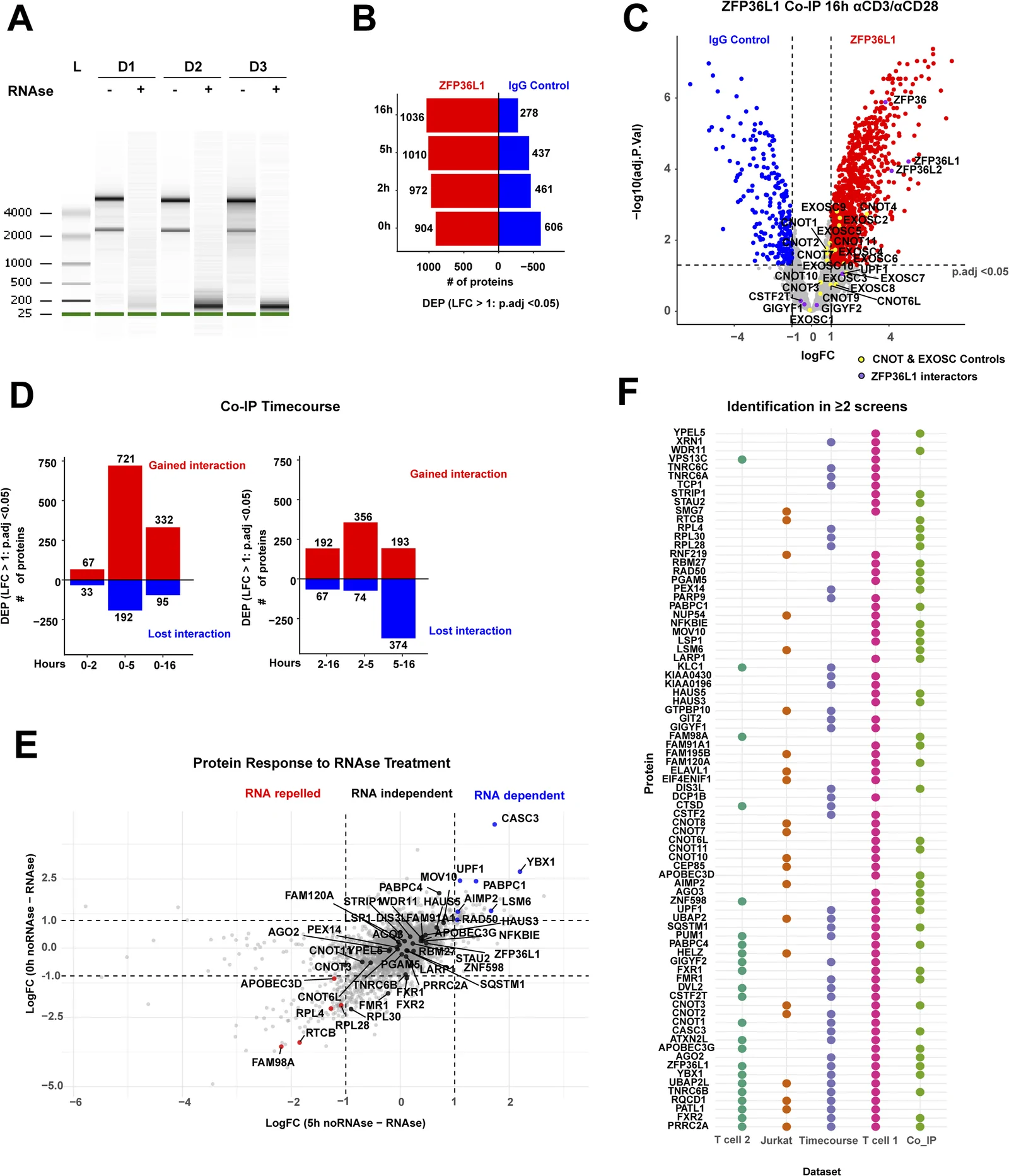
Co-IP reveals RNA-dependent and -independent interactions of ZFP36L1. **(A)** RNA profile of T cell lysates measured by Bioanalyser trace with or without treatment throughout entire protocol with RNAse A (200 U/ml) that were used for the ZFP36L1 Co-IP. (L) = ladder. n = 3 donor pools of 40 donors each. **(B)** Number of proteins that were significantly enriched in the ZFP36L1 Co-IP (Red) versus IgG controls (Blue) (LFC >1, *P*.adj <0.05) at different time points of T cell activation. **(C)** Volcano plot of proteins identified in the ZFP36L1 Co-IP at 16 h of T cell activation. Only proteins identified in all three replicates were considered putative interactors. Yellow dots indicate CNOT and EXOSC proteins. Purple dots indicate proteins that were among the top ZFP36L1 interactors in the UltraID_L1 interactome screens. Proteins with *P*.adj <0.05 and LFC >1 or LFC >−1 were considered significant. **(D)** Number of proteins identified as gained (red) or lost (blue) in ZFP36L1 Co-IP upon RNAse A treatment at indicated time points, compared with the IgG control. Proteins with *P*. adj <0.05 and LFC >1 or LFC >−1 were considered significant. **(E)** Scatter plot analysis of protein responses to RNase treatment between 0 and 5 h of αCD3/αCD28 stimulation. Proteins are classified as RNA-repelled (red), RNA-independent (black), or RNA-dependent (blue). Significance threshold of >1 LFC is indicated by dashed lines. **(F)** Dot plot depicting protein identification across multiple datasets (T cell 1 and 2, Jurkat, Timecourse, Co_IP). Each row represents a different protein, with colored dots indicating presence/detection in each dataset. Proteins are only shown when identified in >2 screens. See Fig 5.

**Figure 5.**
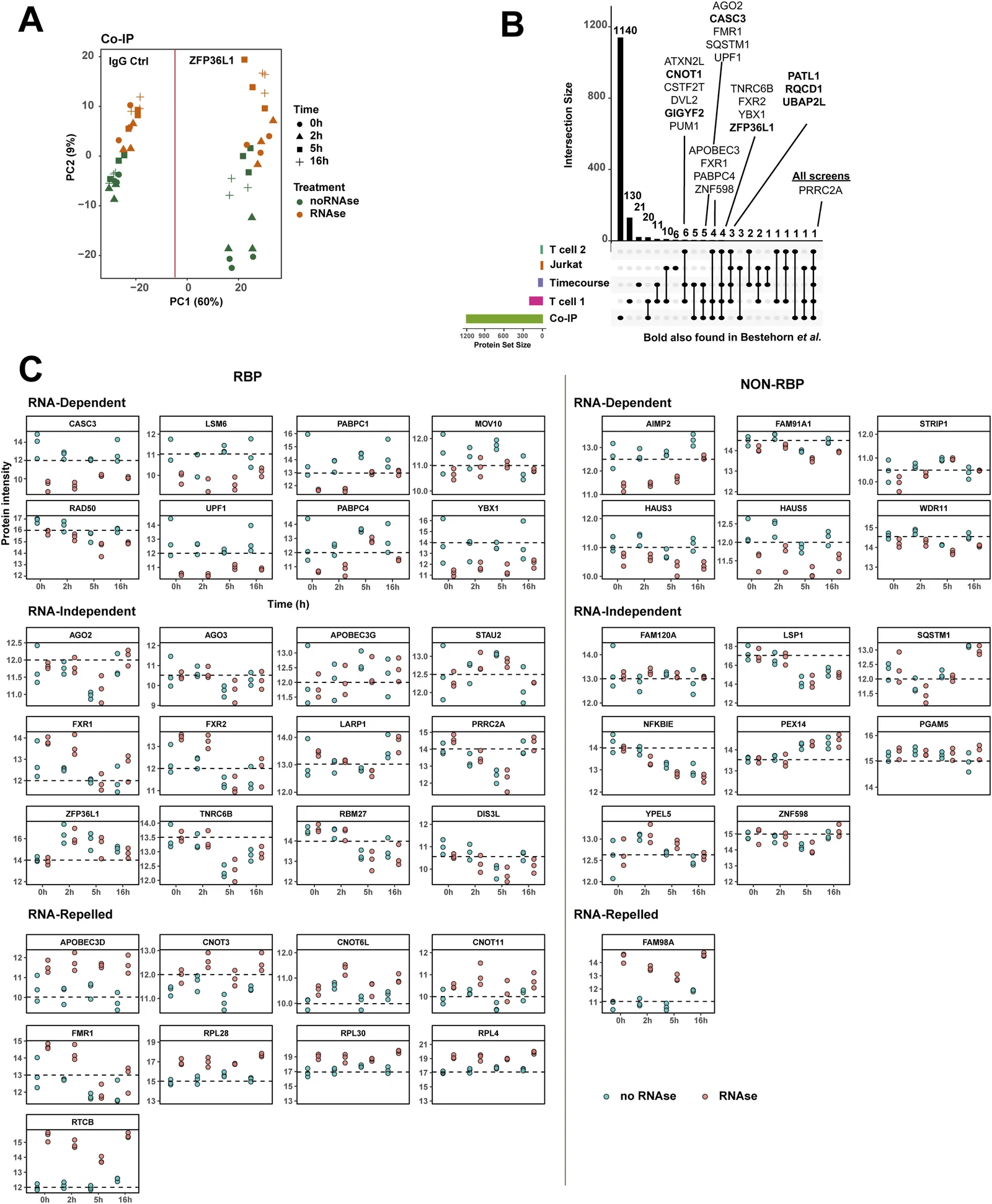
RNA-dependency of interactions with ZFP36L1. Co-IP with ZFP36L1 antibodies and IgG control with T cells lysates generated at indicated time points that were treated or not with 200 U/ml RNAse A during the entire protocol (lysis, incubation & washes). n = 3 donor pools of 40 donors each. **(A)** PCA of indicated conditions. Green dots: RNA present, Orange dots: RNAse A treated. Different shapes represent different timepoints of activation. **(B)** Upset plot of proteins identified as ZFP36L1 interactors in the Co-IP or indicated proximity labeling essay. Numbers refer to numbers of proteins found interacting with ZFP36L1 in indicated conditions. LFC >1, *P*. adj <0.05, shape correlation >0.5. Proteins indicated in bold were also identified in HEK293T cells (Bestehorn et al, 2025). **(C)** LFQ protein intensities from Co-IP time course of 44 proteins co-identified by the Co-IP and at least one proximity labeling (panel B). Green dots: RNA present, Orange dots: RNAse A treated. Proteins were categorized into known RNA-binding proteins (RBPs) and non-RBPs that displayed RNA-dependent, RNA-independent or RNA-repelled interactions with ZFP36L1 (as defined in Fig S5F). n = 3 donor pools of 40 donors each. See Fig S5.

Having deciphered the ZFP36L1 interactome and its kinetics, we next asked whether the interactions with ZFP36L1 required the presence of RNA. To study this, we performed a co-immunoprecipitation (Co-IP) of ZFP36L1 with untreated T-cell lysates or with RNAse pre-treated lysates (Fig S5A). MS analysis revealed that in addition to ZFP36L1 enrichment versus IgG control, treatment with RNAse defined the distribution of samples in the PCA (Figs 5A, S5B and C, and Table S4; LFC≥1; *P*.adj < 0.05).

Table S4. Co-immunoprecipitation for ZFP36L1 to measure RNA dependency of the ZFP36L1 interactome.

The Co-IP executed in the presence of RNA identified several prototypic ZFP36L1 interactors, including the CCR4-NOT and EXOCS proteins, together with its sister proteins ZFP36 and ZFP36L2 (Fig S5C). In line with the proximity labeling time course (Fig 4), the Co-IP also detected ZFP36L1 interactome dynamics throughout T-cell activation, with most differential interactions measured at 5 h of T-cell activation (Fig S5D). The majority of protein interactions with ZPF36L1 did not change with pre-treatment and were thus RNA independent, as exemplified for 0 h and 5 h of T-cell activation (Fig S5E). Yet, some interactions were reduced upon RNAse treatment (RNA-dependent) or even increased (RNA-repelled; Fig S5E). Because the overlap of ZFP36L1 interaction partners was limited between the Co-IP and proximity labeling experiments (Fig 5B), we focused for downstream analysis on the 44 proteins that were co-identified in at least one UltraID screen, and that we therefore considered high-confidence ZFP36L1 interactors (Fig S5F).

RBPs that displayed reduced interactions with ZFP36L1 in the absence of RNA included UPF1 (Fiorini et al, 2015; Musaev et al, 2024), YBX1, MOV10, the exon junction complex protein CASC3 (Zhang et al, 2017), LSM6, a component of the RNA degradation and spliceosome complex (He & Parker, 2000; Khusial et al, 2005), and the poly-A binding proteins PABPC1 and PABPC4 (Qi et al, 2022; Oliveira et al, 2023; Fig 5C, RNA-dependent; Table S4). The preferred interaction with ZFP36L1 in the presence of RNA was constant throughout time for all proteins, except for MOV10, which showed the highest RNA-mediated ZFP36L1 interaction at 5 h of T cell activation (Fig 5C).

Non-RBPs preferentially interacting with ZFP36L1 in the presence of RNA were AIMP2, a scaffold for the tRNA synthetase complex (Hahn et al, 2019; Zhou et al, 2020), in addition to WDR11 and FAM91A1, which belong to the complex that tethers vesicles to the Golgi apparatus (Navarro Negredo et al, 2018). We also identified HAUS3 and HAUS5 involved in microtubule generation (Lawo et al, 2009; Uehara et al, 2009) and STRIP1 (Fam40a), which regulates the cell shape by modulating actin filament dynamics (Bai et al, 2011; Suryavanshi et al, 2018), the latter with strongest effects of RNA depletion at 0 h and 2 h of T cell activation (Fig 5C, RNA-dependent). Interestingly, CNOT proteins showed increased binding to ZFP36L1 when RNA was depleted (Fig 5C, RNA-repelled), suggesting that the interaction to the c-terminal CNOT interacting motif of ZFP36 proteins (Fabian et al, 2013; Otsuka et al, 2020) does not require the presence of RNA to maintain its interaction with ZFP36L1.

Some RBPs were oblivious to RNA depletion for their interaction with ZFP36L1 (AGO2, AGO3, APOBEC3G, Stau2, FXR1, FXR2, LARP1, PRRC2A, TNCRB6B, RBM27, DISL3, and ZFP36L1) (Fig 5C, RNA-independent). As expected, interactions with SQSTM1 (p62) and proteins involved in protein turnover and post-translational modifications did not require RNA (FAM120A, LSP1, NFKBIE, PEX14, PGAM5, YPEL5, the scaffolding protein ZNF598), Fig 5C. Fam98A, which stimulates PRMT1-induced arginine methylation showed even increased interaction with ZFP36L1 when RNA was depleted (Akter et al, 2017; Fig 5C). In conclusion, this analysis uncovers the context-dependent nature of interactions with ZFP36L1.

### UPF1 KO and GIGYF1/2 KO T cells display cellular stress through increased protein translation

Lastly, we aimed to decipher the role of ZFP36L1 candidate interactors in T cells. We focused on 4 proteins that exert divergent post-transcriptional functions: the nonsense-mediated decay factor UPF1, the de-adenylation proteins/translational repressors GIGYF1 and GIGYF2, and the nuclear alternative poly-adenylation regulating protein CSTF2T. UPF1 interacts with ZFP36L1 preferentially at 0 and 16 h, and it does so in an RNA-dependent manner (Figs 4D and 5C). GIGYF1 shows similar interaction kinetics with ZFP36L1 as UPF1 (Fig 4D). Conversely, GIGYF2 and CSTF2T maintain their interaction with ZFP36L1 throughout T-cell activation (Fig 4D). Of note, UPF1, GIGYF1 and GIGYF2 belong to the core ZFP36L1 interactome, being identified also in HEK293T cells (Bestehorn et al, 2025; Figs S2, S5).

**Figure S6.**
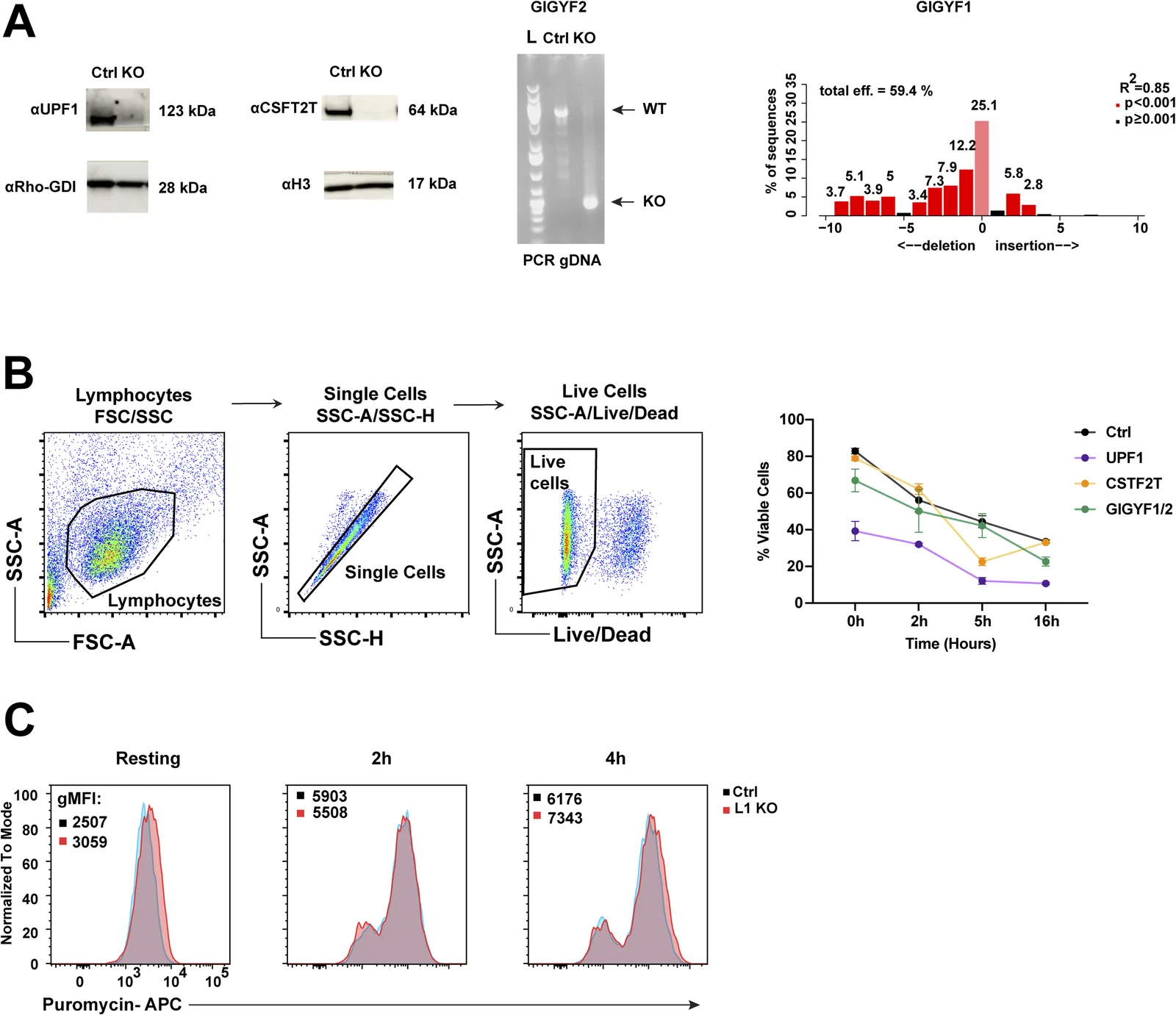
UPF1 and GIGYF1/2 deletion alters T-cell viability. **(A)** Validation of CRISPR-Cas9 gene-editing for indicated RBPs. 6 d after gene editing, efficiency was determined by immunoblot (left panel: UPF1 and CSFT2T, with Rho-GDI and H3 as loading control), by PCR on genomic DNA (middle panel: GIGYF2; arrows indicate PCR product of WT and KO product), or by TIDE analysis (right panel: GIGYF1 KO cells). Representative of n = 9 independent donors. **(B)** Cell viability of indicated T cells as defined by IR-dye through flow cytometry at indicated time points of activation post αCD3/αCD28 re-stimulation. Left: Gating strategy; Right: % viable cells per time point. n = 2 donors. **(C)** Puromycin incorporation assay in ZFP36L1 KO versus WT T cells activated with PMA/Iono for indicated time points, as quantified by flow cytometry. Numbers indicate gMFI values. Representative of three donors. See Fig 6.

**Figure 6.**
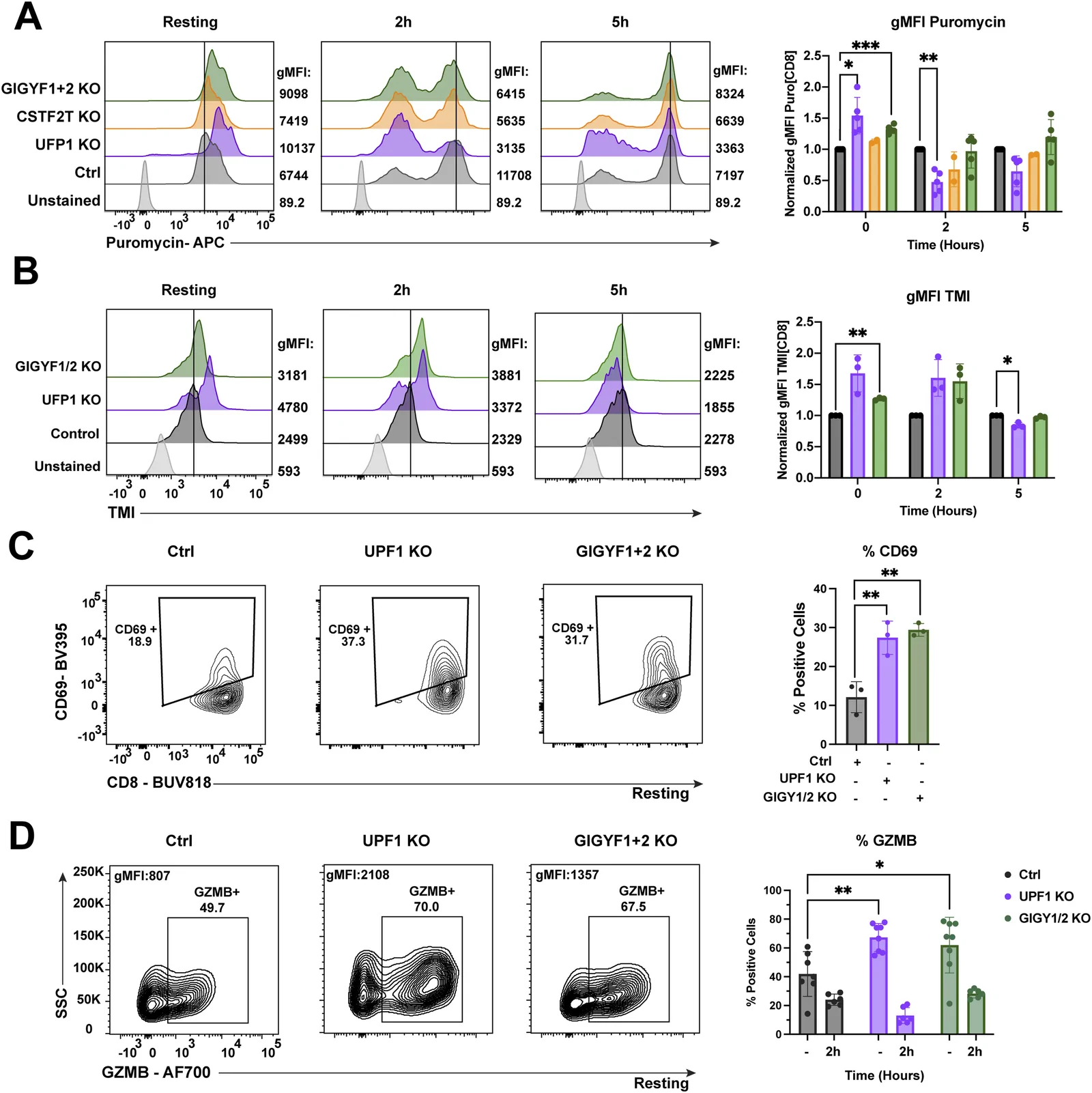
UPF1 and GIGYF1/2 deletion alters translation and stress responses in human CD8+ T cells. **(A)** Puromycin incorporation in indicated CRISPR-Cas9 gene-edited T cells or non-targeting crRNA (ctrl)-edited T cells at indicated time points of activation with αCD3/αCD28. Left panel: Representative plot of puromycin incorporation as quantified by flow cytometry. Geometric Mean Fluorescent Intensity (gMFI) value indicated on the right. Right panel: puromycin gMFI during rest or indicated time of stimulation with αCD3/αCD28. n = 2–5 donors. **(B)** Detection of unfolded proteins with tetraphenyl ethene maleimide (TMI) dye. Left panel: Representative plot of TMI as quantified by flow cytometry. Geometric Mean Fluorescent Intensity (gMFI) value indicated on the right. Right panel: TMI gMFI during rest or indicated time of stimulation with αCD3/αCD28. n = 3 donors. **(C)** Left panel: representative CD69 expression in resting control and indicated KO T cells measured by flow cytometry. Right panel: percentage of CD69^+^ T cells. Data shown as mean ± SD. n = 3 donors. **(D)** Left panel: Representative Granzyme B (GZMB) expression measured by flow cytometry. Right panel: percentage of GZMB + T cells in resting conditions and after 2 h of stimulation with αCD3/αCD28. n = 6 donors, data shown as mean ± SD (right). (A, B Two-way ANOVA with Dunnett’s correction C, D) One-way ANOVA with Dunnett’s correction. *P < 0.05, **P < 0.01, ***P < 0.001. See Fig S6.

We first gene-edited T cells with CRISPR-Cas9 to delete UPF1, CSTF2T, GIGYF1, and GIGYF2 (Fig S6A). Because GIGYF1 and GIGYF2 cooperate with each other (Giovannone et al, 2003), we generated GIGYF1/GIGYF2 double deficient T cells (GIGYF1/2 KO). The viability of GIGYF1/2 KO and CSTF2T KO T cells was comparable to that of control T cells (Fig S6B). Only UPF1 KO T cells were less viable (Fig S6B). We next measured the global translation rate with puromycin incorporation for 10 min. CSTF2T deficiency showed very limited effects in resting and in activated T cells (Fig 6A). In contrast, in resting T cells, UPF1 KO and GIGFYF1/2 KO T cells displayed substantially higher global translation rates compared with control T cells (Fig 6A). T cell activation for 2 h led to a substantial drop in puromycin incorporation in UPF1 KO compared with control T cells, while for GIGFYF1/2 KO T cells the puromycin incorporation dropped to equal levels as control T cells (Fig 6A). This could not merely be attributed to cell viability for GIGYF1/2 KO T cells (Fig S6B); although UPF1 KO displayed high cell death upon activation. Because ZFP36L1 KO T cells showed very limited changes in puromycin incorporation (Fig S6C), we conclude that either other ZFP36 family members may compensate for the loss of ZFP36L1, or that UPF1 and GIGYF1/2 are the driving force in changing the global translation rates.

Higher protein production can lead to unfolded protein response and consequentially, induce cell stress (Cao et al, 2019). We therefore measured the level of unfolded proteins with tetraphenyl ethene maleimide (TMI), which becomes fluorescent when engaging with free thiol side chains that are exposed in unfolded proteins (Chen et al, 2017). Concomitant with lower cell viability, UPF1 KO T cells showed increased levels of unfolded proteins (Fig 6B), Also GIGYF1/2 KO cells had higher levels of TMI staining at resting state, and particularly at 2 h of T-cell activation, although not statistically significant (Fig 6B). After 5 h of activation, however, the levels of unfolded proteins normalized for all control and KO T cells (Fig 6B).

T-cell activation substantially increases the translation rate (Marchingo et al, 2020; Wolf et al, 2020; Sinclair and Cantrell, 2025). We therefore questioned whether the observed higher puromycin incorporation in resting UPF1 KO and GIGYF1/2 KO T cells indicated an altered T-cell activation status. Indeed, the percentage of resting T cells expressing the activation marker CD69 more than doubled in UPF1 KO and GIGYF1/2 KO T cells compared with control T cells (Fig 6C). Granzyme B expression was also substantially higher in resting UPF1 KO and GIGYF1/2 KO T cells, and T-cell activation resulted in stronger release of Granzyme B (=loss of signal), most prominently in UPF1 KO T cells, again pointing to a high T-cell activation state in the absence of T-cell triggers (Fig 6D). Combined, these data suggest that UPF1 and GIGYF1/2 contribute to T-cell fitness by limiting protein translation and activation status in resting T cells.

### UPF1 promotes ZFP36L1 protein production

**Figure 7.**
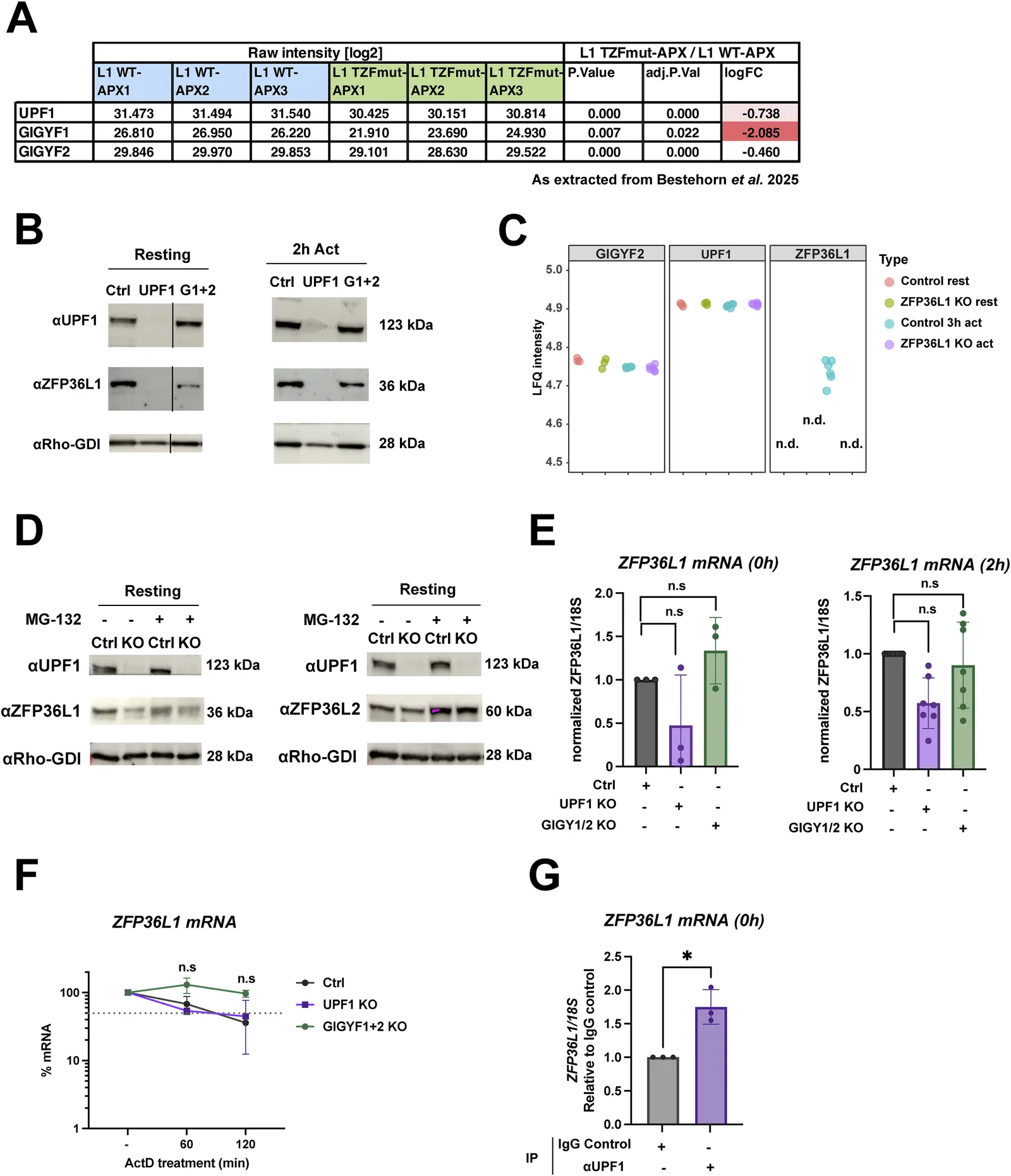
UPF1 interacts with ZFP36L1 mRNA and modulates its expression at the transcript level. **(A)** Quantification of ZFP36L1 interactors identified by immunoprecipitation–mass spectrometry (IP-MS) in HEK293T cells expressing either wild-type or zinc-finger mutant (TZFmut) ZFP36L1. Data extracted from Bestehorn et al (2025). **(B)** Immunoblot of ZFP36L1 and UPF1 in non-targeting crRNA (Ctrl) treated, UPF1 KO (UPF1) and GIGYF1+2 KO (G1+2) from resting or αCD3/αCD28 activated T cells (2 h). aRho-GDI was used as loading control. Representative of at least six donors. **(C)** WT and ZFP36L1 KO cells were analysed using MS. LFQ intensity of GIGYF2, UPF1 and ZFP36L1 from human control and ZFP36L1 gene edited T cells, extracted from MS data depicted at either resting state or 3 h PMA/Iono activations. n = 6 independent donors. n.d = not detected. **(D)** Immunoblot of ZFP36L1, ZFP36L2 and UPF1 in control and UPF1 KO (KO) T cells treated with MG132 or vehicle control for 2 h, executed in resting T cells (left) or 2 h αCD3/αCD28 re-activated T cells (right). Representative of three independent donors. **(E)** *ZFP36L1* mRNA levels in control, UPF1 KO and GIGYF1+2 KO cells at resting state (left) and 2 h of activation (right), measured by RT-qPCR. Data normalized to 18*S*. n = 3 donors. **(F)** RNA stability assay of *ZFP36L1* using actinomycin D treatment started after 2 h of activation. mRNA decay measurements of *ZFP36L1* mRNA. n = 3 donors. **(G)** Native RNA immunoprecipitation (RIP) with UPF1 antibody or IgG isotype control from lysates of resting T cells. qRT-PCR of endogenous mRNAs from RIP of UPF1 relative to IgG control. n = 3 donor pools, mean ± SD. **(E, F, G)** Two-way ANOVA with Dunnett’s correction. *P < 0.05. n.s = non-significant. Source data are available for this figure.

We next sought to decipher the interplay of ZFP36L1 with UPF1 and GIGYF1/2. Re-analysis of the comparative interactome study of wild type ZFP36L1 with a tandem zinc finger (TZF) mutant failing to interact with RNA by Bestehorn et al (2025) revealed that only GIGYF1, but not UPF1 and GIGYF2, lost its interaction with ZFP36L1 when the TZF was mutated (Fig 7A), indicating divergent requirements of these three proteins for interacting with ZFP36L1.

Strikingly, when we measured the effect of UPF1 and GIGYF1/2 depletion on ZFP36L1 protein expression, we found that UPF1 deletion substantially reduced the ZFP36L1 protein levels in both resting and activated T cells (2 h; Fig 7B). Because neither UPF1 nor GIGYF2 protein expression was altered in ZFP36L1 KO T cells (Fig 7C), we conclude that UPF1 may be an upstream regulator of ZFP36L1 protein expression. UPF1 does not modulate the protein stability of ZFP36L1 because blocking the proteasomal degradation with MG132 did not recover ZFP36L1 protein expression in UPF1 KO T cells (Fig 7D). In contrast, proteasomal block restored the protein expression of its sister protein ZFP36L2, irrespective of UPF1 deletion (Fig 7D). Thus, we concluded that UPF1 may regulate the generation of ZFP36L1 protein.

Protein output is in part defined by the number of mRNA templates. Interestingly, ZFP36L1 mRNA expression was reduced in UPF1 KO T cells, but not in GIGYF1/2 KO T cells, yet without overt effects on mRNA stability during the 2 h treatment with the transcription inhibitor Actinomycin D (Fig 7E and F). A native RNA-immunoprecipitation followed by qRT-PCR (RIP-qRT-PCR) on unmodified T cells confirmed that UPF1 interacts with *ZFP36L1* mRNA (Fig 7G), further supporting the hypothesis that UPF1 is an upstream regulator of ZFP36L1 expression. Thus, UPF1 not only interacts with ZFP36L1 protein as uncovered by proximity labeling, but UPF1 also appears to support the protein expression of ZFP36L1, at least in part by regulating its transcript levels, and we hypothesize that the helicase activity of UPF1 could potentially contribute to this. Combined, this finding showcases how proximity labeling can uncover novel protein–protein interactions and help identify novel regulatory nodes of proteins, as exemplified here for ZFP36L1.

## Discussion

In this study, we uncovered the multifaceted nature of the ZFP36L1 interactome in human T cells. We report versatile interaction kinetics with ZFP36L1 during T-cell activation, and we defined how RNA mediates these interactions. This comprehensive interactome analysis positions ZFP36L1 in multiple post-transcriptional regulatory pathways in T cells. Importantly, using primary T cells from different donors throughout this study, instead of using cell lines or clones of generated KO cells, substantially mitigates the risk of studying effects that are merely specific for one clone. Thus, beyond providing new insights in the activity of ZFP36L1, by optimizing proximity labeling for primary T cells, our study paves the way for studying other proteins in primary immune cells.

The proximity labeling presented here uncovered the protein networks in close proximity with ZFP36L1. Beyond its established role in recruiting the CCR4-NOT deadenylation complex (Fabian et al, 2013; Ito-Kureha et al, 2020; Otsuka et al, 2020; Akiyama & Yamamoto, 2021), we detected proximity of ZFP36L1 with not previously described proteins involved in mRNA decapping (PATL1) and mRNA degradation pathways, as well as translation repressors (GIGYF1, GIGYF2, ZNF598, and 4EHP). Notably, many of the newly identified candidate interactors are part of complexes of previously annotated ZFP36L1 interactors. Therefore, it would be interesting to decipher how ZFP36L1 comes in proximity with the different subunits, and to determine whether ZFP36L1 plays an active role in the formation of these RBP complexes. One way to address this question was defining the requirement of RNA for interacting with ZFP36L1. These data clearly depicted the RNA (in)dependency of several candidates. However, it should also be considered that some protein–protein interactions indicated as RNA-independent may initially be mediated by RNA and persist due to high affinity interactions or conformational locking.

A key finding of our study is that the ZFP36L1 interactome undergoes remodeling during T-cell activation. For instance, proteins involved in mRNA degradation and translation repression processes are reduced during early T-cell activation. We hypothesize that this temporary suspension of these interactions may be required to allow for efficient protein synthesis to occur, a key feature to drive rapid remodeling of the T-cell proteome upon T-cell activation (Howden et al, 2019; Wolf et al, 2020; Sinclair & Cantrell, 2025). The subsequent re-establishment of these interactions at 5 h of T-cell activation coincides with the time point when ZFP36L1 commences to dampen the T-cell activation program, including for instance the cytokine production (Petkau et al, 2022; Popović et al, 2023; Zandhuis et al, 2025). How the landscape of interaction partners is altered remains to be established. Because we find very limited alterations in RBP expression levels upon T-cell activation, it is conceivable that post-translational modifications (PTMs) define the alterations in the RBP interactome of ZFP36L1 to allow for the rapid shift in the proteome and production of effector molecules. Supporting this hypothesis, we identified several post-translational modifiers and PTM-binders interacting with ZFP36L1, including the 14-3-3 proteins YWHAB/YWHAZ interacting with post-translational modifications, the protein deadenlyase SIRT2, and the CTLH E3 ubiquitin protein ligase complex component YELP5 (Fig 3). Future studies should address which signaling pathways downstream of the T-cell receptor and costimulatory molecules drive these modifications and thus alter ZFP36L1’s interactions and its activity. It will also be interesting to define to which extend the ZFP36L1 interactome is identical or unique in specific T-cell subsets (CD4^+^ versus CD8^+^ T cells) or other cell types (e.g., macrophage cell lines [Bestehorn et al, 2025]). The comparative analysis with macrophages we provide here may not be complete, provided that the datasets in macrophages were generated with APEX labeling, in addition to being performed in different labs. In our hands, adding H_2_O_2_ (required for APEX labeling) to primary T cells resulted in immediate cell death, thus impeding the use of this method in T cells. Likewise, adding biotin to the culture media required for T cell survival may have resulted in higher background proximity labeling due to bona fide low-level proximity labeling by residual biotin present in the T cell cultures, which also holds true for minimum incubation time with biotin of 2 h we used here, instead of the regular 10-min time point. Also protein stickiness during pulldown or specific propensities of proteins can result in background measurements. Therefore, to increase confidence in calling ZFP36L1 interactors, we routinely included control measurements without adding additional biotin. Yet, the risk of off-target labeling can never be fully excluded, and downstream validation assays for targets of interest is warranted. Irrespective of these technical challenges that come with studying biological processes in primary human T cells, the time-lapse proximity labeling during T cell activation could effectively delineate that the ZFP36L1 interactome is context-dependent and versatile.

The advantage of proximity labeling is that it can detect interactions in living cells, capturing physiologically relevant interactions in their native cellular context, including weak and transient interactions (Roux et al, 2012; Branon et al, 2018). In particular transient interactions are typically lost during cell lysis procedures required for, e.g., Co-IP. For instance, whereas GIGYF1, GIGYF2, and CSTF2T interactions were clearly measured with proximity labeling, we failed to detect these interactions over background in the ZFP36L1 Co-IP. Furthermore, proximity labeling identified interactions of ZFP36L1 with nuclear factors such as the polyadenylation factors CSTF2 and CSTF2T, and the RNA decapping protein PATL1, which can shuttle between nucleus and cytoplasm (Marnef et al, 2012). Moreover, we also identified proteins in proximity to ZFP36L1 that are associated with granules (G3BP1 FXR1/2, ATXN2L, PUM1, YBX1, UBAP2/L, MOV10) (Curdy et al, 2023), structures, or membrane-bound organelles (NUPL1; nuclear pore complex [Ishikawa et al, 1997]), and these cellular compartments are generally disrupted during cell lysis. This included also interactions with non-RBPs, like SIRT2, YELP5, and SQSTM1. Interestingly, neither Bestehorn et al (2025) nor we identified ZFP36 or ZFP36L2 as interaction partners for ZFP36L1 in the proximity labeling assay, which is in line with recent reports indicating that ZFP36 proteins exert similar functions, but show different expression kinetics upon T-cell activation, and rather appear to act independently, and in a time-dependent manner (Jones Evans et al, 2025; Zandhuis et al, 2025).

Our investigation of functional interactions also revealed a previously uncharacterized relationship between UPF1 and ZFP36L1. UPF1, a central component of the nonsense-mediated mRNA decay pathway (Sun et al, 1998; Fiorini et al, 2015; Kim & Maquat, 2019; Musaev et al, 2024), interacts with ZFP36L1 protein primarily in resting T cells and at later time points of T-cell activation when translation activity is returning back toward levels of resting T cells. UPF1 orchestrates co-translational mRNA surveillance by recruiting the endonuclease SMG6 and UPF2 (Langer et al, 2024). These two proteins were not identified by proximity labeling as ZFP36L1 interactors with SMG6 or UPF2. Therefore, it remains to be determined whether the helicase activity of UPF1 supports the entry of ZFP36L1 to its target mRNA together with SMG6 and UPF2, or whether it exerts a fundamentally different activity with ZFP36L1.

UPF1, however, also appears to directly regulate the protein expression of ZFP36L1. ZFP36L1 protein expression is rapidly induced upon T-cell activation (Petkau et al, 2022; Popović et al, 2023; Zandhuis et al, 2025). In the absence of UPF1, ZFP36L1 protein expression in resting and activated T cells is substantially impaired. UPF1 binds to ZFP36L1 mRNA, and its depletion reduces the overall mRNA levels of ZFP36L1 mRNA, at least in part by acting on ZFP36L1 mRNA stability. UPF1 is a RNA helicase and is generally involved in promoting RNA decay through several mechanisms (Kim & Maquat, 2019; Staszewski et al, 2023). Our finding that UPF1 depletion rather increases mRNA degradation of ZFP36L1 could indicate that UPF1 depletion prevents another RNA stabilizing protein to interact with ZFP36L1, or alternatively, that it alters the subcellular localization of *ZFP36L1* mRNA and thereby promotes its degradation. Rescue experiments with UPF1 mutants could shed light on its mode of activity. However, the limited viability after UPF1 depletion in primary T cells prohibited such follow up experiments that require additional genetic modifications. Irrespective of its exact mode of action in the context of ZFP36L1, our findings identify UPF1 as an upstream regulator of ZFP36L1 protein expression, and they suggest that UPF1 possibly monitors and thereby safeguards the quality of ZFP36L1 transcripts, allowing for its protein production to occur. This, in combination with the evidence for ZFP36L1:UPF1 interaction, could implicate that ZFP36L1 can indirectly regulate its own levels through UPF1.

In conclusion, our findings position ZFP36L1 within a complex network of post-transcriptional regulatory mechanisms in T cells. Through its dynamic interactions with components of mRNA decay, translation repression, and potentially mitochondrial translation machineries, ZFP36L1 appears equipped to orchestrate the temporal sequence of gene/protein expression events required for proper T-cell activation. The regulatory relationship between UPF1 and ZFP36L1 highlights the intricate interdependence of post-transcriptional control systems and creates new opportunities for understanding how these mechanisms shape T-cell function in immunity and disease.

## Materials and Methods

### Cell culture

Peripheral blood mononuclear cells (PBMCs) not for transfusion from anonymized healthy donors were used in accordance with the Declaration of Helsinki (Seventh Revision, 2013) after written informed consent (Sanquin) and approval of study NVT258.01 by the donor physicians of Sanquin. PBMCs were isolated by Lymphoprep density gradient separation (Stemcell Technologies) and cryopreserved until further use. Human T cells and Jurkat cells were cultured in Culture Medium (Iscove’s Modified Dubecco’s Medium [IMDM] supplemented with 10% fetal bovine serum [FBS], 100 U/ml penicillin, 100 μg/ml streptomycin, and 2 mM L-glutamine supplemented with 100 IU/ml recombinant human rhIL2 [Protech] and 10 ng/mL rhIL-15 [Protech]). Of note, because biotin deprivation induces stress and inflammatory response in T cells (Elahi et al, 2018), T cells were cultured in biotin-containing media (53 nM). Human fibrosarcoma FLYRD18 cells (ADCC) were cultured in IMDM supplemented with 10% FBS, 1% L-glutamine, 1% Penicillin-Streptomycin antibiotic solution at 37°C, 5% CO_2_ and split every 2 d at 1:10. All cells were cultured at 37°C with 5% CO_2_.

### T-cell activation

T cells from defrosted PBMCs were activated for 48 h as previously described (Nicolet & Wolkers, 2022). Briefly, 24-well plates were pre-coated overnight at 4°C with 4 μg/ml rat a-mouse IgG2a (MW1483; Sanquin) in phosphate-buffered saline (PBS). Plates were washed with PBS and coated for >3 h with 1 μg/ml αCD3 (HIT3a; BioLegend) at 37°C. 1.3 × 10^6^ PBMCs/well were seeded with 1 μg/ml soluble αCD28 (CD28.2; BioLegend) in 1 ml Iscove’s Modified Dubecco’s Medium (IMDM) supplemented with 10% fetal bovine serum (FBS), 100 U/ml penicillin, 100 μg/ml streptomycin, and 2 mM L-glutamine. After 48 h of activation at 37°C, 5% CO_2_, cells were harvested and cultured in standing T25/T75 tissue culture flasks (Thermo Fisher Scientific) at a density of 0.8 × 10^6^/ml in culture medium. Media was refreshed every 2–3 d. Upon nucleofection, T cells were cultured in T-cell mixed media (Miltenyi) supplemented with 5% heat-inactivated human serum, 5% FBS, 100 U/ml penicillin, 100 μg/ml streptomycin, 2 mM L-glutamine, 100 IU/ml rhIL-2 (Peprotech), and 10 ng/ml rhIL-15 (Peprotech) for 4 d after switching back to Culture Medium for another 3 d. T cells were re-activated in Culture Medium in 96-wells plates with 10 ng/mL Phorbol Myristate Acetate (PMA) and 1 μm Ionomycin, or with αCD3 (Pelicluster; Sanquin) and αCD28 (BioLegend), 1 μg/ml each. For proteasomal inhibition, 2 μm of MG-132 (Sigma-Aldrich) was used.

### Generation UltraID constructs

UltraID_ZFP36L1_GFP or UltraID_GFP fusion proteins were generated as gBlock DNA by IDT. These were then transferred into the pMX_Puro backbone (Addgene) into ClaI and EcoRI restriction sites according to the manufacturer’s protocol (T4; NEB) (Table 2). pMX_UltraID was generated by digesting pMX_UltarID_ZFP36L1_P2A_GFP vector with NotI and ligating it back together to remove ZFP36L1_P2A_GFP. Plasmid DNA was amplified in DH5α super-competent bacteria (NEB) and isolated with NucleoSpin Plasmid Transfection-grade Mini-Prep isolation kit (Machery-Nagel). Sequences were confirmed with Sanger sequencing.

### Virus production and retroviral transduction

1 × 10^5^ FLYRD18 cells/well were pre-seeded in 6-well culture plates and cultured overnight at 37°C. Transfection was carried out with GeneJammer (Agilent) according to the manufacturer’s protocol. Cells were cultured for 48 h at 32°C. αCD3/αCD28-activated T cells (48 h) were retrovirally transduced as previously described (Popović et al, 2023). Briefly, non-tissue culture treated 24-well plates were pre-coated overnight with 50 μg/ml Retronectin (Takara). Plates were washed once with PBS, 800 μl viral supernatant/well was added and spun at 4°C at 2,820 g. Supernatant was removed and 1 × 10^6^ T cells/well were added, plates spun for 5 min at 180 g and incubated overnight at 37°C. Supernatant was replaced with fresh Culture Medium. After 48 h, T cells were harvested and cultured in T25/T75 flasks for 6–8 d as described above.

### Flow cytometry

To analyze GFP protein expression, T cells were washed with FACS buffer (PBS, containing 1% FBS and 2 mM EDTA) and labeled for 20 min at 4°C with indicated antibodies (see Table 1). Dead cells were excluded with Near-IR (Life Technologies). For intracellular cytokine staining, cells were cultured with 1 μg/ml brefeldin A for indicated time points, fixed, and permeabilized with Cytofix/Cytoperm kit according to the manufacturer’s protocol (BD Biosciences) prior to acquisition using FACSymphony. Data were analyzed with FlowJo (BD Biosciences, version 10).

**Table 1.** All recourses used in materials methods

Antibodies & dilution used	Source	Identifier
Rat anti-mouse IgG2a (clone MW1483) – 4 μg/ml	Sanquin	n/a
Mouse anti-CD28 (clone CD28.2) – 1 μg/ml	BioLegend	Cat# 302902; RRID:AB_314304
Mouse anti-CD3 (clone HIT3a) 1 μg/ml	BioLegend	Cat# 300302; RRID:AB_314038
Pelicluster CD3 - 1 μg/ml	Sanquin	Cat#564364
BUV737 mouse anti-human CD4 (clone SK3) – 1:300	BD Bioscience	Cat# 564305; RRID:AB_2713927
BUV805 mouse anti-human CD8 (clone SK1) - 1:300	BD Bioscience	Cat# 612889; RRID:AB_2833078
BUV395 mouse anti-human CD69 (clone FN50) - 1:200	BD Bioscience;	Cat# 564364; RRID:AB_2738770
Brilliant Violet 785 anti-human TNF (clone MAb11) - 1:300	BioLegend	Cat# 502948; RRID:AB_2565858
eFluor 450 mouse anti-human IFN gamma (clone 4S.B3) -1:300	eBiosciences	Cat# 48-7319-42; RRID:AB_2043866
APC anti-human IL2 (clone MQ1-17H12)- 1:300	BioLegend	Cat# 500310; RRID:AB_3083299
Alexa Fluor 647 mouse anti-human CD137 (clone 4B4-1) - 1:300	BioLegend	Cat# 309824; RRID:AB_2566258
LIVE/DEAD Fixable Near-IR Dead Cell Stain Kit, for 633 or 635 nm excitation – 1:800	Invitrogen	Cat# L34976
CellTrace Violet© - 0.25µm	Thermo Fisher Scientific	​
UPF1 rabbit anti-human polyclonal antibody – 1:500	Proteintech	Cat# 23379-1-AP ; RRID:AB_11232421
CSTF2T rabbit anti-human polyclonal - 1:500	Proteintech	Cat# 12419-1-AP; RRID:AB2230895
ZFP36L1 rabbit polyclonal antibody clone 1A3- 1:1,000	Sigma-Aldrich	Cat# WH0000677M2 RRID: AB_1844265
ZFP36L1 mouse anti-human monoclonal antibody - 1:1,000	Abcam	Cat# ab42473 RRID: AB_883662
ZFP36L2 rabbit anti-mouse polyclonal antibody- 1:500	Abcam	Cat# ab70775 RRID: AB_1271231
ZFP36 rabbit anti-mouse polyclonal antibody 1:500	Origene	Cat# OTI3D10
Mouse anti-RhoGDI - 1:1,000	Abnova	Cat# 89-113-917; RRID:AB
Rabbit polyclonal IgG – 1mg/ml	Sigma-Aldrich	Cat# 12-370
Mouse anti-rabbit IgG light chain (HRP) – 1:10,000	Abcam	Cat# ab99697; RRID:AB_10673897
Anti-DDX6 polyclonal antibody – 1mg/ml	Protein Tech	Cat# 14632-1-AP; RRID:AB_2091264
Bacterial and viral strains	​	​
E. coli DH5alpha super competent bacteria	​	Invitrogen Cat#1 8265017
Biological samples	​	​
Human PBMC	Sanquin	n/a
Chemicals, peptides, and recombinant proteins	​	​
Trypsin Gold, Mass Spectrometry Grade	Promega	Cat# V5280
GolgiPlug Protein Transport Inhibitor (containing Brefeldin A)	BD Biosciences	Cat# BD 555029
Recombinant human IL-15	Peprotech	Cat# 200-15
Recombinant human IL2	Peprotech	Cat# 200-02
GeneJammer	Agilent	Cat# 204130
RetroNectin	Takara Bio	Cat# T100B
Phorbol 12-myristate 13-acetate (PMA)	Sigma-Aldrich	Cat# P8139
Ionomycin calcium salt	Sigma-Aldrich	Cat# 13909
RNase A	Thermo Fisher Scientific	Cat# EN0531
Precision Counting Beads	BioLegend	Cat#424902
GelRed Nucleic Acid Gel Stain	Biotium	Cat# 41003
Actinomycin D	Sigma-Aldrich	Cat# A9415
Alt-R S.p. Cas9 Nuclease V3	DT	ICat# 10000735
Power SYBR Green PCR Master Mix	Applied Biosystems	Cat# 4367660
RNase A, DNase and protease-free (10 mg/ml)	Thermo Fisher Scientific	Cat# EN0531
RNaseOUT Recombinant Ribonuclease Inhibitor	Invitrogen	ICat# 10777019
Ribonucleoside Vanadyl Complex	New England Biolabs	Cat# S1402S
Halt Protease and Phosphatase Inhibitor Single-Use Cocktail, EDTA-Free (100X)	Thermo Fisher Scientific	Cat# 78443
Digitonin, High Purity	Calbiochem	Cat# 300410
Lympholyte-M	Cederlane	Cat# CL5035
Dynabeads Protein A for Immunoprecipitation	Invitrogen	Cat# 10008D
Dynabeads Protein G for Immunoprecipitation	Invitrogen	Cat# 10003D
Streptavidin Sepharose High Performance	GE Healthcare	Cat# GE17-5113-01
Critical commercial assays	​	​
NucleoSpin Plasmid Transfection grade, Mini kit for ultrapure plasmid DNA	Machery-Nagel	​
Fixation/Permeabilization Solution Kit with BD GolgiPlug	BD Biosciences	Cat# 555028
P2 Primary Cell 4D-Nucleofector X Kit S	Lonza	Cat# V4XP-2032
SuperScript III First-Strand Synthesis System	Invitrogen	Cat# 18080051
Equipment	​	​
VerityPro Thermal Cycler	Thermo Fisher Scientific	n/a
Mupid One Electrophoresis System	Nippon Genetics Europe	n/a
NGS platform	Illumina	n/a
NanoDrop 2000	Thermo Fisher Scientific	n/a
BD LSR Fortessa Cell Analyzer	BD Biosciences	n/a
FACS Symphony A5 Cell Analyzer	BD Biosciences	n/a
Amershamm ImageQuant 800	Cytiva	n/a
Casy Cell counter	Roche Innovatis	n/a
4D-Nucleofector X Unit	Lonza	Cat# AAF-1003X
Deposited data	​	​
Raw data files Mass Spectrometry analysis (ProteomeXchange)	This paper	PRIDE: PXD056415
Experimental models: Cell lines	​	​
Human: FLYRD18 cells	ECACC	Cat# 95091902
Human: Jurkat cell line	​	​
Recombinant DNA	​	​
GFP-TIS11B	Ma and Mayr (2018)	Addgene Cat # 121157
pSF3-UltraID	Kubitz et al (2022)	Addgene Cat # 172878
Software and algorithms	​	​
FlowJo version 10.8.1	BD Biosciences	https://www.flowjo.com/
GraphPad Prism version 9.1.1	GraphPad Software	https://www.graphpad.com/
R environment for statistical computing	The R foundation	https://www.r-project.org/
RStudio Version 2024.12.1+563	RStudio	https://www.rstudio.com/
StepOne Software v2.3	Applied Biosystems	https://www.thermofisher.com/nl/en/home/technical-resources/softwaredownloads/StepOne-andStepOnePlus-Real-Time-PCRSystem.html
Maxquant 1.6.10	Cox et al (2011)	https://www.maxquant.org/
MS data analysis script for R studio	R studio script	https://github.com/ajhoogendijk/ZFP36L1_UltraID

Not limited to, antibodies and dilutions, bacterial and viral strains, biological samples, chemicals, peptides, and recombinant proteins, critical commercial assays, equipment, deposited data, experimental models: cell lines, recombinant DNA and software and algorithms.

### Immunoblotting

Cell lysates (1 × 10^6^ cells) were prepared by standard procedures using RIPA lysis buffer (Thermo) supplemented with protease and phosphatase inhibitors (Thermo). Lysates were run on 4–12% SDS–PAGE (Thermo). SDS–PAGE gels were directly transferred onto nitrocellulose membranes (iBlot2, Thermo). Membranes were blocked with 5% BSA TBST solution (Fraction V, Sigma-Aldrich) and incubated overnight with α-Streptavidin-HRP, α-UPF1 (Proteintech), α-CSTF2T (Proteintech), ZFP36L1 (ab42473; Abcam), α-ZFP36L2 (Abcam), α-DDX6 (Protein Tech), or α-RhoGDI (Abnova), followed by α-Rabbit (Southern Biotech) or α-Mouse (Southern Biotech) HRP-conjugated secondary antibodies.

### UltraID

Proximity-dependent biotin identification assay was performed according to Kubitz et al (2022), with the following modifications. Retrovirally transduced Jurkat cells and human T cells were FACS-sorted based on GFP expression 3 d post transduction and expanded for another 7 d. Cells were activated with αCD3/αCD28 for 2–16 h or left unactivated (rested) in the presence or absence of (50-) 500 μm biotin dissolved in DMSO and incubated in the dark at 37°C. Cells were harvested, washed twice with ice-cold PBS, and lysed in 1 ml lysis buffer (10 mM Tris/HCl pH 7.5, 150 mM NaCl, 0.5 mM EDTA + HALT Protease/Phosphatase Inhibitor cocktail [1861281; Thermo] + 0.5% NP40 [vol/vol]) for 5 min in at 4°C. Lysates were sonicated 3 × 30 s using a Branson Sonifier 450 device, keeping cells on ice for 1 min in between sessions. Cell lysates were cleared by centrifugation 14,000*g* at 4°C for 20 min. Cleared lysates were added to pre-washed 200 μl Dynabeads MyOne Streptavidin C1 (65002; Invitrogen) beads (2× wash with PBS, equilibrated in lysis buffer for 5 min at 4°C and washed with lysis buffer). Mix was incubated overnight rotating at 4°C. The following day, beads were washed 3× using wash buffer 1 (10 mM Tris/HCl pH 7.5, 150 mM NaCl, 0.5 mM EDTA + HALT Protease/Phosphatase Inhibitor cocktail (1861281; Thermo)) and wash buffer 2 (10 mM Tris/HCl pH 7.5, 150 mM NaCl). Proteins were digested on-bead and prepared for Mass Spectrometry analysis as described below.

### Co-immunoprecipitation

For ZFP36L1 Co-IP: Cytoplasmic lysates of 50 × 10^6^ αCD3/αCD28-activated human CD3^+^ T cells were prepared (simultaneously prepared from same donor pools cells of the UltraID timecourse experiment of Fig 4) using lysis buffer (140 mM NaCl, 5 mM MgCl2, 20 mM Tris/HCl pH7.6, 1% Digitonin) freshly supplemented with 1% protease inhibitor cocktail (Sigma-Aldrich). Protein A Dynabeads (Invitrogen) were prepared according to the manufacturer’s protocol. Cell lysate was pretreated with 200 U/ml RNAse A (Thermo Fisher Scientific), immunoprecipitated for 4 h at 4°C with 10 μg polyclonal rabbit a-ZFP36L1 (1A3; Sigma-Aldrich) or with isotype control (12–370; Sigma-Aldrich) ± 200 U/ml RNAse A (Thermo Fisher Scientific). Beads were washed twice with wash buffer (150 mM NaCl, 10 mM Tris/HCl pH7.6, 2 mM EDTA, protease/phosphatase inhibitor cocktail) and twice with 10 mM Tris/HCl pH7.6. Immunoprecipitated proteins were reduced and on-bead alkylated. Proteins were detached with 250 ng trypsin for 2 h at 20°C. Beads were removed and proteins were further digested into peptides with 350 ng trypsin for 16 h. Peptides were prepared for MS analysis, as described below.

For DDX6 Co-IP: Co-immunoprecipitation was performed using the Pierce Classic Magnetic IP/Co-IP Kit (Thermo Fisher Scientific Scientific) according to the manufacturer’s instructions. Briefly, 1.5 × 10^6^ CD8^+^ T cells per condition were lysed in IP Lysis/Wash Buffer and clarified by centrifugation. For each immunoprecipitation, 1 μg of polyclonal antibody against DDX6 (ProteinTech), or Rabbit IgG control polyclonal antibody (Sigma-Aldrich) was added to the lysate and incubated at 4°C to allow immune complex formation. Subsequent binding to Protein A/G magnetic beads was performed according to the manufacturer’s protocol. After overnight incubation, beads were washed five times using a wash buffer consisting of 0.025 M Tris, 0.15 M NaCl, and 0.001 M EDTA. Immunoblotting was performed as described above.

### Mass spectrometry data acquisition

For the UltraID T cell screen 1 and Jurkat screen, tryptic peptides were separated by nanoscale C18 reverse phase chromatography coupled online to an Orbitrap Fusion Lumos Tribrid mass spectrometer (Thermo Fisher Scientific) via a nanoelectrospray ion source (Nanospray Flex Ion Source, Thermo Fisher Scientific). Peptides were loaded on a 20 cm 75–360 μm inner-outer diameter fused silica emitter (New Objective) packed in-house with ReproSil-Pur C18-AQ, 1.9 μm resin (Dr Maisch GmbH). The column was installed on a Dionex Ultimate3000 RSLC nanoSystem (Thermo Fisher Scientific) using a MicroTee union formatted for 360 μm outer diameter columns (IDEX) and a liquid junction. The spray voltage was set to 2.15 kV. Buffer A was composed of 0.1% formic acid and buffer B of 0.1% formic acid, 80% acetonitrile. Peptides were loaded for 17 min at 300 nl/min at 5% buffer B, equilibrated for 5 min at 5% buffer B (17–22 min) and eluted by increasing buffer B from 5-28% (22–80 min) and 28–40% (80–85 min), followed by a 5 min wash to 95% and a 5 min regeneration to 5%. Survey scans of peptide precursors from 375 to 1500 m/z were performed at 120 K resolution (at 200 m/z) with a 4 × 10^5^ ion count target. Tandem mass spectrometry was performed by isolation with the quadrupole with isolation window 0.7, HCD fragmentation with normalized collision energy of 30, and rapid scan mass spectrometry analysis in the ion trap. The MS2 ion count target was set to 3 × 10^4^, and the max injection time was 20 ms. Only those precursors with charge state 2–7 were sampled for MS2. The dynamic exclusion duration was set to 20 s with a 10 ppm tolerance around the selected precursor and its isotopes. Monoisotopic precursor selection was turned on. The instrument was run in top speed mode with 1 s cycles. All data were acquired with Xcalibur SII software (Kreft et al, 2024). For the UltraID T cell sreen 2, UltraID timecourse screen, and ZFP36L1 Co-IP, samples were measured using TimsTOF-DIA as previously described (Laan et al, 2025). One amendment was made to the protocol, which encompassed that 30 samples per day were measured instead of 60. For the total cell lysates, cells were lysed and acquired as previously described (Groten et al, 2024), using the Orbitrap Fusion Lumos Tribbrid mass spectrometer.

### Mass spectrometry data analysis

Raw mass spectrometry files were processed with the MaxQuant computational platform, version 1.6.2.10. Proteins and peptides were identified using the Andromeda search engine by querying the reviewed human Uniprot database (downloaded 09-22-2023 20426 entries). Standard settings with the additional options match between runs, and unique peptides for quantification were selected. The generated “proteingroups.txt” data were imported in R4.30/Rstudio 2024.04.1. “reverse,” “potential contaminants,” and “only identified by site” peptides were filtered out and label free quantification values were log2 transformed. Missing values were imputed by a normal distribution (width = 0.3, shift = 1.8), assuming these proteins were close to the detection limit. Statistical analyses were performed using moderated t-tests in the LIMMA package. A Benjamini-Hochberg adjusted *P*-value <0.05 and absolute log2 fold change >1 was considered statistically significant and relevant.

For supervised classification, we generated theoretical profiles in which conditions of interest intensities were set to an arbitrary high and low in all other samples (Kreft et al, 2023). These profiles were correlated with all statistically significant proteins. Correlation between data and theoretical protein profiles was performed using Pearson correlations, a ρ > 0.5 or 0.6 and Benjamini-Hochberg adjusted *P*-value <0.05 was used as threshold. Of note, the correlation threshold is set depending on the number of control samples and depth of data acquisition. Gene ontology overrepresentation analyses were performed using the clusterprofiler package (Yu et al, 2012). The R package ggplot2 was used for graphical representations. Scripts for MS data analysis are available at: https://github.com/ajhoogendijk/ZFP36L1_UltraID.

### Genetic modification of T cells with Cas9 RNPs

crRNAs were designed in Benchling (https://benchling.com; Table 2). Cas9 RNP production and T cell nucleofection was performed as previously described (Freen-van Heeren et al, 2020). Briefly, Alt-R crRNA and tracrRNA were reconstituted to 100 μm in Nuclease Free Duplex buffer (all IDT). As a negative control, nontargeting negative control crRNA #1 was used (IDT). Oligos were mixed at equimolar ratios (i.e., 4.5 ml total crRNA + 4.5 ml tracrRNA) in nuclease-free PCR tubes and denatured by heating at 95°C for 5 min. Nucleic acids were cooled down to room temperature prior to mixing them with 30 μg Alt-R S.p. Cas9 Nuclease V3 (IDT) to produce Cas9 ribonuclear proteins (RNPs). Mixture was incubated at room temperature for at least 10 min prior to nucleofection. For nucleofection, human CD3^+^ T cells activated for 48 h with αCD3/αCD28 were washed twice with PBS and resuspended in P2 buffer (Lonza). Cells were electroporated in 16-well strips in a 4D Nucleofector X unit (Lonza) with program EH100 for human T cells &P2 buffer. Knockout efficiency was determined 5 d after electroporation by Western blot, or by PCR.

**Table 2.** All oligonucleotide and sgRNA sequences used in manuscript.

Oligos and sgRNA sequences	​
Alt-R CRISPR-Cas9 tracrRNA	IDT Cat#1072534
Alt-R CRISPR-Cas9 Negative Control crRNA #1	IDT Cat# 1072544
GIGYF1 sgRNA sequence 1	GCA​GGA​CAA​GGA​GTT​CGC​CG
GIGYF1 sgRNA sequence 2	TGG​CTC​GTC​CTG​CAG​CAC​CG
GIGYF2sgRNA sequence 1	GGC​GAC​TAG​CTG​GAT​CAA​GG
GIGYF2 sgRNA sequence 2	GGG​AAC​ACA​TGG​AAC​GAC​GT
CSTF2T sgRNA sequence 1	CCT​AGA​GCT​CCT​ATA​CCT​CG
CSTF2T sgRNA sequence 2	GAT​GCT​CCA​AAT​GAC​CCA​CG
UPF1 sgRNA sequence 1	GTC​CTG​GAG​TGC​TAC​AAC​TG
UPF1 sgRNA sequence 2	GAT​ATG​CCT​GCG​GTA​CAA​AG
RPS18 Forward primer	CAG​AAG​TGA​CGC​AGC​CCT​CTA
RPS18 Reverse primer	AGA​CAA​CAA​GCT​CCG​TGA​AGA
UltraID_ZFP36L1_GFP gBlock	gag​gaa​ttc​ggc​cgg​ccc​cat​gga​aca​aaa​act​cat​ctc​aga​aga​gga​tct​cga​ctt​caa​gaa​cct​gat​ctg​gct​gaa​gga​ggt​gga​cag​cac​cca​gga​gag​act​gaa​gga​gtg​gaa​cgt​gtc​cta​cgg​cac​cgc​cct​ggt​ggc​cga​cag​aca​gac​caa​ggg​cag​agg​cgg​ccc​ggg​cag​aaa​gtg​gct​gag​cca​gga​ggg​cgg​cct​gta​ctt​cag​ctt​cct​gct​gaa​ccc​caa​gga​gtt​cga​gaa​cct​gct​gca​gct​gcc​cct​ggt​gct​ggg​cct​gag​cgt​gag​cga​ggc​cct​gga​gga​gat​cac​cga​gat​ccc​ctt​cag​cct​gaa​gtg​gcc​caa​cga​cgt​gta​ctt​cca​gga​gaa​gaa​ggt​gag​cgg​cgt​gct​gtg​cga​gct​gag​caa​gga​caa​gct​gat​cgt​ggg​cat​cgg​cat​caa​cgt​gaa​cca​gag​aga​gat​ccc​cga​gga​gat​caa​gga​cag​agc​cac​cac​cct​gta​cga​gat​cac​cgg​caa​gga​ctg​gga​cag​aaa​gga​ggt​gct​gct​gaa​ggt​gct​gaa​gag​aat​cag​cga​gaa​cct​gaa​gaa​gtt​caa​gga​gaa​ggt​cga​cgg​gcg​gcc​gcc​aac​cac​cac​cct​cgt​gtc​tgc​cac​cat​ctt​cga​ctt​gag​cga​agt​ttt​atg​caa​ggg​taa​caa​gat​gct​caa​cta​tag​tgc​tcc​cag​tgc​agg​ggg​ttg​cct​gct​gga​cag​aaa​ggc​agt​ggg​cac​ccc​tgc​tgg​tgg​ggg​ctt​ccc​tcg​gag​gca​ctc​agt​cac​cct​gcc​cag​ctc​caa​gtt​cca​cca​gaa​cca​gct​cct​cag​cag​cct​caa​ggg​tga​gcc​agc​ccc​cgc​tct​gag​ctc​gcg​aga​cag​ccg​ctt​ccg​aga​ccg​ctc​ctt​ctc​gga​agg​ggg​cga​gcg​gct​gct​gcc​cac​cca​gaa​gca​gcc​cgg​ggg​cgg​cca​ggt​caa​ctc​cag​ccg​cta​caa​gac​gga​gct​gtg​ccg​ccc​ctt​tga​gga​aaa​cgg​tgc​ctg​taa​gta​cgg​gga​caa​gtg​cca​gtt​cgc​aca​cgg​cat​cca​cga​gct​ccg​cag​cct​gac​ccg​cca​ccc​caa​gta​caa​gac​gga​gct​gtg​ccg​cac​ctt​cca​cac​cat​cgg​ctt​ttg​ccc​cta​cgg​gcc​ccg​ctg​cca​ctt​cat​cca​caa​cgc​tga​aga​gcg​ccg​tgc​cct​ggc​cgg​ggc​ccg​gga​cct​ctc​cgc​tga​ccg​tcc​ccg​cct​cca​gca​tag​ctt​tag​ctt​tgc​tgg​gtt​tcc​cag​tgc​cgc​tgc​cac​cgc​cgc​tgc​cac​cgg​gct​gct​gga​cag​ccc​cac​gtc​cat​cac​ccc​acc​ccc​tat​tct​gag​cgc​cga​tga​cct​cct​ggg​ctc​acc​tac​cct​gcc​cga​tgg​cac​caa​taa​ccc​ttt​tgc​ctt​ctc​cag​cca​gga​gct​ggc​aag​cct​ctt​tgc​ccc​tag​cat​ggg​gct​gcc​cgg​ggg​tgg​ctc​ccc​gac​cac​ctt​cct​ctt​ccg​gcc​cat​gtc​cga​gtc​ccc​tca​cat​gtt​tga​ctc​tcc​ccc​cag​ccc​tca​gga​ttc​tct​ctc​gga​cca​gga​ggg​cta​cct​gag​cag​ctc​cag​cag​cag​cca​cag​tgg​ctc​aga​ctc​ccc​gac​ctt​gga​caa​ctc​aag​acg​cct​gcc​cat​ctt​cag​cag​act​ttc​cat​ctc​aga​tga​cag​atc​tgc​tac​taa​ttt​ctc​ctt​gct​taa​gca​agc​tgg​tga​tgt​tga​aga​aaa​tcc​tgg​tcc​tat​ggt​gag​caa​ggg​cga​gga​gct​gtt​cac​cgg​ggt​ggt​gcc​cat​cct​ggt​cga​gct​gga​cgg​cga​cgt​aaa​cgg​cca​caa​gtt​cag​cgt​gtc​tgg​cga​ggg​cga​ggg​cga​tgc​cac​cta​cgg​caa​gct​gac​cct​gaa​gtt​cat​ctg​cac​cac​cgg​caa​gct​gcc​cgt​gcc​ctg​gcc​cac​cct​cgt​gac​cac​cct​gac​cta​cgg​cgt​gca​gtg​ctt​cag​ccg​cta​ccc​cga​cca​cat​gaa​gca​gca​cga​ctt​ctt​caa​gtc​cgc​cat​gcc​cga​agg​cta​cgt​cca​gga​gcg​cac​cat​ctt​ctt​caa​gga​cga​cgg​caa​cta​caa​gac​ccg​cgc​cga​ggt​gaa​gtt​cga​ggg​cga​cac​cct​ggt​gaa​ccg​cat​cga​gct​gaa​ggg​cat​cga​ctt​caa​gga​gga​cgg​caa​cat​cct​ggg​gca​caa​gct​gga​gta​caa​cta​caa​cag​cca​caa​cgt​cta​tat​cat​ggc​cga​caa​gca​gaa​gaa​cgg​cat​caa​ggc​gaa​ctt​caa​gat​ccg​cca​caa​cat​cga​gga​cgg​cag​cgt​gca​gct​cgc​cga​cca​cta​cca​gca​gaa​cac​ccc​cat​cgg​cga​cgg​ccc​cgt​gct​gct​gcc​cga​caa​cca​cta​cct​gag​cac​cca​gtc​cgc​cct​gag​caa​aga​ccc​caa​cga​gaa​gcg​cga​tca​cat​ggt​cct​gct​gga​gtt​cgt​gac​cgc​cgc​cgg​gat​cac​tct​cgg​cat​gga​cga​gct​gta​caa​gta​agt​ggc​cgc​atc​gat​ggg
UltraID_GFP gBlock	gag​gaa​ttc​ggc​cgg​ccc​cat​gga​aca​aaa​act​cat​ctc​aga​aga​gga​tct​cga​ctt​caa​gaa​cct​gat​ctg​gct​gaa​gga​ggt​gga​cag​cac​cca​gga​gag​act​gaa​gga​gtg​gaa​cgt​gtc​cta​cgg​cac​cgc​cct​ggt​ggc​cga​cag​aca​gac​caa​ggg​cag​agg​cgg​ccc​ggg​cag​aaa​gtg​gct​gag​cca​gga​ggg​cgg​cct​gta​ctt​cag​ctt​cct​gct​gaa​ccc​caa​gga​gtt​cga​gaa​cct​gct​gca​gct​gcc​cct​ggt​gct​ggg​cct​gag​cgt​gag​cga​ggc​cct​gga​gga​gat​cac​cga​gat​ccc​ctt​cag​cct​gaa​gtg​gcc​caa​cga​cgt​gta​ctt​cca​gga​gaa​gaa​ggt​gag​cgg​cgt​gct​gtg​cga​gct​gag​caa​gga​caa​gct​gat​cgt​ggg​cat​cgg​cat​caa​cgt​gaa​cca​gag​aga​gat​ccc​cga​gga​gat​caa​gga​cag​agc​cac​cac​cct​gta​cga​gat​cac​cgg​caa​gga​ctg​gga​cag​aaa​gga​ggt​gct​gct​gaa​ggt​gct​gaa​gag​aat​cag​cga​gaa​cct​gaa​gaa​gtt​caa​gga​gaa​ggt​cga​cgg​gcg​gcc​gcc​agt​cat​ggt​gag​caa​ggg​cga​gga​gct​gtt​cac​cgg​ggt​ggt​gcc​cat​cct​ggt​cga​gct​gga​cgg​cga​cgt​aaa​cgg​cca​caa​gtt​cag​cgt​gtc​tgg​cga​ggg​cga​ggg​cga​tgc​cac​cta​cgg​caa​gct​gac​cct​gaa​gtt​cat​ctg​cac​cac​cgg​caa​gct​gcc​cgt​gcc​ctg​gcc​cac​cct​cgt​gac​cac​cct​gac​cta​cgg​cgt​gca​gtg​ctt​cag​ccg​cta​ccc​cga​cca​cat​gaa​gca​gca​cga​ctt​ctt​caa​gtc​cgc​cat​gcc​cga​agg​cta​cgt​cca​gga​gcg​cac​cat​ctt​ctt​caa​gga​cga​cgg​caa​cta​caa​gac​ccg​cgc​cga​ggt​gaa​gtt​cga​ggg​cga​cac​cct​ggt​gaa​ccg​cat​cga​gct​gaa​ggg​cat​cga​ctt​caa​gga​gga​cgg​caa​cat​cct​ggg​gca​caa​gct​gga​gta​caa​cta​caa​cag​cca​caa​cgt​cta​tat​cat​ggc​cga​caa​gca​gaa​gaa​cgg​cat​caa​ggc​gaa​ctt​caa​gat​ccg​cca​caa​cat​cga​gga​cgg​cag​cgt​gca​gct​cgc​cga​cca​cta​cca​gca​gaa​cac​ccc​cat​cgg​cga​cgg​ccc​cgt​gct​gct​gcc​cga​caa​cca​cta​cct​gag​cac​cca​gtc​cgc​cct​gag​caa​aga​ccc​caa​cga​gaa​gcg​cga​tca​cat​ggt​cct​gct​gga​gtt​cgt​gac​cgc​cgc​cgg​gat​cac​tct​cgg​cat​gga​cga​gct​gta​caa​gta​agt​ggc​atg​cga​tgg​g

### RNA immunoprecipitation

Cytoplasmic lysates of 25 × 10^6^ human CD3^+^ T cells activated for 3 h with αCD3/αCD28 were prepared using lysis buffer (10 mM HEPES, pH 7.0, 100 mM KCl, 5 mM MgCl2, 0.5% NP40) freshly supplemented with 1 mM DTT, 100 U/ml RNase OUT (both Invitrogen), 0.4 mM Ribonucleoside Vanadyl Complex (NEB) and 1% EDTA-free protease/phosphatase inhibitor cocktail (Thermo Fisher Scientific). Protein G Dynabeads (Invitrogen) were prepared according to manufacturer’s protocol. The lysate was immunoprecipitated for 4 h at 4°C with 10 μg polyclonal α-UPF1 (ProteinTech) or a polyclonal rabbit IgG isotype control (12–370; Sigma-Aldrich). RNA was extracted directly from beads by using Zymo Quick-RNA Miniprep Kit, and mRNA expression was measured by RT-PCR as described above.

### Quantitative PCR analysis

T cells were activated for indicated time point with αCD3/αCD28. For mRNA half-life measurements, cells were treated for an additional 1 or 2 h with 5 μg/ml actinomycin D (ActD) (Sigma-Aldrich). Total RNA was extracted using Quick-RNA MiniPrep plus kip (Zymo). cDNA was synthesized with SuperScript III (Invitrogen), RT-PCR was performed using SYBR Green on a StepOne Plus (Applied Biosystems). Ct values were normalized to RPS18 levels.

### Quantification and statistical analysis

Results are shown as mean ± SD. Statistical analysis was performed with GraphPad Prism 10 with Wilcoxon two-tailed ratio paired or unpaired Student’s *t* test when comparing two groups, or with one-way ANOVA test with Dunnett correction when comparing more than two groups. *P* values <0.05 were considered statistically significant.

## Supplementary Material

Reviewer comments

## Data Availability

All data are available in the main text or the supplementary materials. The mass spectrometry proteomics data of the RNA Aptamer Pulldown have been deposited to the ProteomeXchange Consortium via the PRIDE (Perez-Riverol et al, 2022) partner repository with the dataset identifier PXD061869. This paper does not report original custom code. All codes used in this paper are available at: https://github.com/ajhoogendijk/ZFP36L1_UltraID. Any additional information required to reanalyze the data reported in this paper is available from the lead contact upon request.
