# Supplementary material for: Proximity labeling puts ZFP36L1 as central hub for posttranscriptional regulation networks in T cells
Source: Life Sci Alliance. 2026 Sep 9;9(11):e202603825. doi: 10.26508/lsa.202603825 (PMC13558869; doi:10.26508/lsa.202603825)
Supplement: Supplementary file 1 [file LSA-2026-03825_review_history.pdf]

Proximity labeling puts ZFP36L1 as central hub for posttranscriptional regulation networks in Tcells

Anouk Jurgens, Branka Popovic, Floris van Alphen, Maxime Steinmetz, Leyma Wardak, Antonia Bradaric, Sander Engels, Carmen van der Zwaan, Maartje van den Biggelaar, Arie Hoogendijk, Julien Béthune, and Monika Wolkers
DOI: https://doi.org/10.26508/lsa.202603825

Corresponding author(s): Monika Wolkers, Sanquin

Review Timeline:

Submission Date:	2026-06-22
Editorial Decision:	2026-07-22
Revision Received:	2026-08-23
Accepted:	2026-08-25

Scientific Editor: Sarita Hebbar

Transaction Report:

(Note: With the exception of the correction of typographical or spelling errors that could be a source of ambiguity, letters and reports are not edited. The original formatting of letters and referee reports may not be reflected in this compilation.)

Please note that the manuscript was reviewed at *Review Commons* and these reports were taken into account in the decision-making process at *Life Science Alliance.*

Reviews

Review #1

Evidence, reproducibility and clarity (Required)

The authors map the ZFP36L1 protein interactome in human T cells using UltraID proximity labeling combined with quantitative mass spectrometry. They optimize labeling conditions in primary T cells, profile resting and activated cells, and include a time course at 2, 5, and 16 hours. They complement the interactome with co-immunoprecipitation in the presence or absence of RNase to assess RNA dependence. They then test selected candidates using CRISPR knockouts in primary T cells, focusing on UPF1 and GIGYF1/2, and report effects on global translation, stress, activation markers, and ZFP36L1 protein levels. The work argues that ZFP36L1 sits at the center of multiple post-transcriptional pathways in T cells (which in itself is not a novel finding) and that UPF1 supports ZFP36L1 expression at the mRNA and protein level. The main model system is primary human T cells, with some data in Jurkat cells.

The core datasets show thousands of identified proteins in total lysates and enriched biotinylated fractions. Known partners from CCR4-NOT, decapping, stress granules, and P-bodies appear, with additional candidates like GIGYF1/2, PATL1, DDX6, and UPF1. Time-resolved labeling suggests shifts in proximity during early activation. Co-IP with and without RNase suggests both RNA-dependent and RNA-independent contacts. CRISPR loss of UPF1 or GIGYF1/2 increases translation at rest and elevates activation markers, and UPF1 loss reduces ZFP36L1 protein and mRNA while MG132 does not rescue protein levels; UPF1 RIP enriches ZFP36L1 mRNA.

Among patterns worth noting are that the activation state drives the principal variance in both proteome and proximity datasets. Deadenylation, decapping, and granule proteins are consistently near ZFP36L1 across conditions, while some contacts dip at 2 hours and recover by 5 to 16 hours. Mitochondrial ribosomal proteins become more proximal later. UPF1 and GIGYF1 show time-linked behavior and RNase sensitivity that fits roles in mRNA surveillance and translational control. These observations support a dynamic hub model, though they remain proximity-based rather than direct binding maps.

Major comments

The key conclusions are directionally convincing for a broad and dynamic ZFP36L1 neighborhood in human T cells. The data robustly recover established complexes and add plausible candidates. The time-course and RNase experiments strengthen the claim that interactions shift with activation state and RNA context. The functional tests around UPF1 and GIGYF1/2 point to biological relevance. That said, some statements could be qualified. The statement that ZFP36L1 "coordinates" multiple pathways implies mechanism and directionality that proximity data alone cannot prove. I suggest reframing as "positions ZFP36L1 within" or "supports a model where ZFP36L1 sits within" these networks.

UPF1, as an upstream regulator of ZFP36L1 expression, is a promising lead. The reduction of ZFP36L1 protein and mRNA in UPF1 knockout, the non-rescue by MG132, and the UPF1 RIP on ZFP36L1 mRNA together argue that UPF1 influences ZFP36L1 transcript output or processing. This claim would read stronger with one short rescue or perturbation that pins the mechanism. A compact test would be UPF1 re-expression in UPF1-deficient T cells with wild-type and helicase-dead alleles. This is realistic in primary T cells using mRNA electroporation or virus-based systems. Approximate time 2 to 3 weeks, including guide design check and expansion. Reagents and sequencing about 2 to 4k USD depending on donor numbers. This would help separate viability or stress effects from a direct role in ZFP36L1 mRNA handling.

The inference that ZFP36L1 proximity to decapping and deadenylation complexes reflects pathway engagement is reasonable and, frankly, expected. Still, where the manuscript moves from proximity to function, the narrative works best when supported by orthogonal validation. Two compact additions would raise confidence without opening new lines of work. First, a small set of reciprocal co-IPs for PATL1 or DDX6 at endogenous levels in activated T cells, run with and without RNase, would tie the RNase-class assignments to biochemistry. Second, a short-pulse proximity experiment using a reduced biotin dose and shorter labeling window in activated cells would address whether long incubations drive non-specific labeling. Both are feasible in 2 to 3 weeks with minimal extra cost for antibodies and MS runs if the facility is in-house.

Reproducibility is helped by donor pooling, repeated T-cell screens, Jurkat confirmation, and detailed methods including MaxQuant, LIMMA, and supervised patterning. Deposition of MS data is listed. The authors should consider adding a brief, stand-alone analysis notebook in SI or on GitHub with exact filtering thresholds and "shape" definitions, since the supervised profiles are central to claims. This would let others reproduce figures from raw tables with the same code and workflows.

Replication and statistics are mostly adequate for discovery proteomics. The thresholds are clear, and PCA and correlation frameworks are appropriate. For functional readouts in edited T cells, please make the number of donors and independent experiments explicit in figure legends, and indicate whether statistics are paired by donor. Where viability differs (UPF1), note any gating strategies used to avoid bias in puromycin or activation marker measurements. These clarifications are quick to add.

Minor comments

The UltraID optimization in primary T cells is useful, but the long 16-hour labeling and high biotin should be framed as a compromise rather than a standard. A short statement about potential off-target labeling during extended incubations would set expectations and justify the RNase and time-course controls.

The overlap across T-cell screens and with HEK293T APEX datasets is discussed, but a compact quantitative reconciliation would help. A table that lists shared versus cell-type-specific interactors with brief notes on known expression patterns would make this point concrete.

Figures are generally clear. Where proximity and total proteome PCA are shown, consider adding sample-wise annotations for donor pools and activation time to help readers link variance to biology. Ensure all volcano plots and heatmaps display the exact cutoffs used in text.

Prior work on ZFP36 family roles in decay, deadenylation via CCR4-NOT, granules, and translational control is cited within the manuscript. In a few places, recent proximity and interactome papers could be more explicitly integrated when comparing overlap, especially where conclusions differ by cell type. A concise paragraph in Discussion that lays out what is truly new in primary T cells would help clarify the contribution of this work to the field.

Significance (Required)

Nature and type of advance. The study is a technical and contextual advance in mapping ZFP36L1 proximity partners directly in human primary T cells during activation. The combination of time-resolved labeling and RNase-class assignments is informative. The CRISPR perturbations provide an initial functional bridge from proximity to phenotype, especially for UPF1.

Context in the literature. ZFP36 family proteins have long been linked to ARE-mediated decay, CCR4-NOT recruitment, and granule localization. The present work confirms those cores and extends them to include decapping and GIGYF1/2-4EHP scaffolds in primary T cells with temporal resolution. The UPF1 link to ZFP36L1 expression adds a plausible surveillance angle that merits follow-up. The cell-type specificity analysis versus HEK293T underscores that proximity networks vary with context.

Audience. Readers in RNA biology, T-cell biology, and proteomics will find the dataset valuable. Groups studying post-transcriptional regulation in immunity can use the resource to prioritize candidate nodes for mechanistic work.

Expertise and scope. I work on post-transcriptional regulation, RNA-protein complexes, and T-cell effector biology. I am comfortable evaluating the conceptual claims, experimental design, and statistical treatment. I am not a mass spectrometry specialist, so I rely on the presented parameters and deposited data for MS acquisition specifics.

To conclude, the manuscript delivers a substantive proximity map of ZFP36L1 in human T cells, with useful temporal and RNA-class information. The UPF1 observations are promising and would benefit from a compact rescue to secure causality. A few minor additions for biochemical validation and transparency in replication would further strengthen the paper.

3. How much time do you estimate the authors will need to complete the suggested revisions:

Estimated time to Complete Revisions (Required)

(Decision Recommendation)

Less than 1 month

***Review Commons* values the work of reviewers and encourages them to get credit for their work. Select 'Yes' below to register your reviewing activity at [Web of Science Reviewer Recognition Service]() (formerly Publons); note that the content of your review will not be visible on Web of Science.**

Web of Science Reviewer Recognition

Yes

Review #2

Evidence, reproducibility and clarity (Required)

The manuscript by Wolkers and colleagues describes the protein interactome of the RNA-binding protein ZFP36L1 in primary human T-cells. There is inherent value in the use of primary cells of human origin, but there is also value in that the study is quite complete, as it is performed in a variety of conditions: T-cells that have been activated or not, at different time points after activation, and by two methods (co-IP and proximity labeling). One might imagine that this basically covers all what can be detected for this protein in T-cells. The authors report a large amount of new interactors involved at all steps in post-transcriptional regulation. In addition, the authors show that UPF1, a known interactor of ZFP36L1, actually binds to ZFP36L1 mRNA and enhances its levels. In sum, the work provides a valuable resource of ZFP36L1 interactors. Yet, how the data add to the mechanistic understanding of ZFP36L1 functions and/or regulation of ZFP36L1 remains unclear.

Major comments:

1) Fig 2: It is confusing that the Pearson correlation to define ZFP36L1 interactors is changed depending on figure panel. In panels A-C, a correlation {greater than or equal to} 0.6 is used, while panel D uses a correlation > 0.5, which changes the nº of interactors. Then, this is changed again in Fig 3A for some cell types but not for others. Why has this been done? It would be better to stick to the same thresholds throughout the manuscript.

2) Fig 3A: It would be nice to have the information of this Figure panel as a Table (protein name, molecular process(es), known or novel, previously detected in which cells) in addition to the figure.

3) Fig 6: To what extent are the effects of UPF1 and GIGFYF1 knock-out on translation and T-cell hyper-activation mediated by ZFP36L1? If deletion of ZFP36L1 itself has no effect on these processes, it seems unlikely that it is involved. In this respect, I am not sure that Fig 6 contributes to the understanding of ZFP36L.

4) Fig 7E: Differences in ZFP36L1 mRNA expression are claimed as a consequence of UPF1 deletion, and indeed there is a clear tendency to reduction of ZFP36L1 mRNA levels upon UPF1 KO. Yet the difference is statistically non-significant. Please, repeat this experiment to increase statistical significance. In addition, a clear discussion on how UPF1 -generally associated to mRNA degradation- contributes to increase ZFP36L1 mRNA levels would be appreciated.

5) Fig 6A: The decrease in global translation by GIGFYF1 knock-out upon activation claimed by the authors is not clear in Fig 6A and is non-significant upon quantification. Please, modify narrative accordingly.

6) Page 6: The authors state 'This included the PAN2/3 complex proteins which trim poly(A) tails prior to mRNA degradation through the CCR4/NOT complex'. To the best of my knowledge, the CCR4/NOT complex does not degrade the body of the mRNA. Both PAN2/3 and CCR4/NOT are deadenylases that function independently.

7) Please, label all Table sheets. Right now one has to guess what is being shown in most of them. Furthermore, it would be convenient to join all Tables related to the same Figure in one unique Excel with several sheets, rather than having many Tables with only one sheet each.

Minor comments:

8) Fig 1E: Shouldn't there be a better separation by biotinylation in the UltraID IP principal component analysis? In theory, only biotinylated proteins should be immunoprecipitated.

9) Fig 3B-E: Is the labeling not swapped, top (always +) is Biotin and bottom (- or +) is aCD3/aCD28?

10) Fig 7A data is from another paper, so I suggest to move this panel to Supplementary materials.

11) Fig S1A: Why is there so much labeling in the UltraID only lane without biotin?

12) Fig S1E: Please, explain better. What is WT?

13) Fig S4B: Please, explain the labels on top of the shapes.

14) Page 3: A time-course of incubation with biotin is lacking in Fig S1B, and thereby it is confusing that the authors direct readers to this figure when an increased to 16h incubation is claimed to be better.

Significance:

Significance (Required)

Strengths: A thorough repository of ZFP36L1 interactors in primary human T-cells. A valuable resource for the community.

Weaknesses: There is little mechanistic insight on ZFP36L1 function or regulation.

How much time do you estimate the authors will need to complete the suggested revisions:

Estimated time to Complete Revisions (Required)

(Decision Recommendation)

Between 1 and 3 months

***Review Commons* values the work of reviewers and encourages them to get credit for their work. Select 'Yes' below to register your reviewing activity at [Web of Science Reviewer Recognition Service] (formerly Publons); note that the content of your review will not be visible on Web of Science.**

Web of Science Reviewer Recognition

Yes

Review #3

Evidence, reproducibility and clarity (Required)

The authors have analyzed the interactome of ZFP36L1 in primary human T cells using a biotin-based proximity labeling method. In addition to proteins that are known to interact with ZFP36L1, the authors defined a multitude of novel interactions involved in mRNA decapping, mRNA degradation pathways, translation repressors, stress granule/p-body formation, and other regulatory pathways. Time-lapse proximity labeling revealed that the ZFP36L1 interactome undergoes remodeling during T cell activation. Co-IP for ZFP36L1 executed in the presence/absence of RNA further revealed the interactome and possible regulators of ZFP36L1, including the helicase UPF1. In addition to interacting with ZFP36L1, UPF1 promotes the ZFP36L1 protein expression, seemingly by binding to the ZFP36L1 mRNA transcript, and in some way stabilizing it. This comprehensive interactome map highlights the widespread interactions of ZFP36L1 with proteins of many types, and its potential roles in diverse T cell processes. Although somewhat descriptive, rather than hypothesis-testing, this work represents an important contribution to understanding the potential roles of the ZFP36 family proteins, and sets up many future experiments which could test molecular details.

Major points:

1) Can the authors discuss the specificity of the antibody for ZFP36L1 used in the Co-IP experiments? The antibody listed in Appendix A is abcam catalog number ab42473, although the catalog number for this antibody (unlike the others major ones used) is not listed in the Methods section - please add this to the Methods to make it easier for

readers to find this detail. Could this antibody also be immunoprecipitating ZFP36 or ZFP36L2? Other antibodies have had cross-reactivity for the different family members. It is also notable that this antibody has been discontinued by the manufacturer (https://www.abcam.com/en-us/products/unavailable/zfp36l1-antibody-ab42473). Have the authors tried the current abcam anti-ZFP36L1 antibody being sold, catalog number ab230507?

2) On this point, the authors report interactions between ZFP36L1 and its related proteins ZFP36 and ZFP36L2 in the Co-IP experiment (Supp 5C). Did these proteins interact in the proximity labeling? Ideally this could be discussed in the Discussion section.

3) Can the authors discuss more fully the limited overlap in identified interactors across the two proximity labeling screens performed in primary T cells (Fig 2C)? Likewise, can the authors comment on the very limited overlap between the screens in T cells and the published ZFP36L1-APEX proximity labelling experiment performed in the HEK293T cell line by Bestehorn et al. (ref 42)? Only 6.8% of proteins found in either T cell screen were found as interactors in this cell line. The authors comment that this may be because "...either expression of certain proteins is cell-type specific, or [because] ZFP36L1 has cell-type specific protein interactions, in addition to its core interactome". While I agree that cell-type specific interactions may be at play, I would think most of the interactors found in the T cell screens are widely expressed proteins necessary for central cell functions.

Minor comments:

4) In Figure 3D, the legend states that black circles indicate significantly enriched proteins in biotin samples, while grey circles indicate non-significant enrichment. However, some genes, including DCP1A, DDX6, YBX1, have black circles in the -biotin group and grey in the +biotin group, which creates confusion in interpretation.

5) Did the authors find any interactors whose expression is known to be specific to CD4 or CD8 T cells?

Significance (Required)

The authors present the first comprehensive analysis of the ZFP36L1 interactome in primary T cells. The use of biotin-based proximity labeling enables detection of physiologically relevant interactions in live cells. This approach revealed many novel interactors.

Strengths include the overall richness of the dataset, and the hypothesis-provoking experiments that could follow in the future. Limitations include somewhat limited overlap with a published proximity labeling dataset from performed in a different cell line, suggesting that there may be artifacts in one or both datasets.

The audience for this article would include those interested broadly in RNA binding proteins and those interested in post-transcriptional and translational regulation.

I have immunology expertise on T cell activation and differentiation and expertise on transcriptional and post-transcriptional regulation of gene expression in T cells.

How much time do you estimate the authors will need to complete the suggested revisions:

Estimated time to Complete Revisions (Required)

(Decision Recommendation)

Between 1 and 3 months

***Review Commons* values the work of reviewers and encourages them to get credit for their work. Select 'Yes' below to register your reviewing activity at Web of Science Reviewer Recognition Service (formerly Publons); note that the content of your review will not be visible on Web of Science.**

Web of Science Reviewer Recognition

No

Manuscript number: RC-2025-03185
Corresponding author(s): Monika Wolkers

1. General Statements

In this study we set out to generate a comprehensive and time-resolved map of the ZFP36L1 protein neighborhood in primary human T cells. Using UltraID-based proximity labeling, co-immunoprecipitation ± RNase, and CRISPR perturbations in primary T cells (with supporting data in Jurkat cells), we define a robust core interactome that links ZFP36L1 to deadenylation, decapping, mRNA surveillance, granule formation, translational control, and more, and identify UPF1 and GIGYF1/2 as functionally relevant proximity partners. We view the resulting datasets as a toolbox and resource for the RNA/T-cell community, providing hypotheses and candidates for future in-depth mechanistic work, with the UPF1–ZFP36L1 axis as one such example.

We are grateful to the reviewers for their thorough and constructive assessments. In the revision we have (i) clarified our wording so that we place ZFP36L1 "within" post-transcriptional networks rather than implying directional coordination; (ii) strengthened the resource aspect by adding comparative tables, clearer statistics, and code/threshold documentation; (iii) better explained technical constraints and design choices (biotin dependence of primary T cells, labeling times, antibody specificity, correlation cut-offs); and (iv) explicitly framed the UPF1 data as yielding first indications of mechanism that however warrant further follow-up for full dissection. We believe these changes improve transparency and reproducibility while preserving the central value of the work as a well-annotated interactome of ZFP36L1 in human T cells.

2. Description of the planned revisions: None

3. Description of the revisions that have already been incorporated in the transferred manuscript

Reviewer #1 (Evidence, reproducibility and clarity (Required)):

The authors map the ZFP36L1 protein interactome in human T cells using UltraID proximity labeling combined with quantitative mass spectrometry. They optimize labeling conditions in primary T cells, profile resting and activated cells, and include a time course at 2, 5, and 16 hours. They complement the interactome with co-immunoprecipitation in the presence or absence of RNase to assess RNA dependence. They then test selected candidates using CRISPR knockouts in primary T cells, focusing on UPF1 and GIGYF1/2, and report effects on global translation, stress, activation markers, and ZFP36L1 protein levels. The work argues that ZFP36L1 sits at the center of multiple post-transcriptional pathways in T cells (which in itself is not a novel finding) and that UPF1 supports ZFP36L1 expression at the mRNA and protein level. The main model system is primary human T cells, with some data in Jurkat cells.
The core datasets show thousands of identified proteins in total lysates and enriched biotinylated fractions. Known partners from CCR4-NOT, decapping, stress granules, and P-bodies appear, with additional candidates like GIGYF1/2, PATL1, DDX6, and UPF1. Time-resolved labeling suggests shifts in proximity during early activation. Co-IP with and without RNase suggests both RNA-dependent and RNA-independent contacts. CRISPR loss of UPF1 or GIGYF1/2 increases translation at rest and elevates activation markers, and UPF1 loss reduces ZFP36L1 protein and mRNA while MG132 does not rescue protein levels; UPF1 RIP enriches ZFP36L1 mRNA.
Among patterns worth noting are that the activation state drives the principal variance in both proteome and proximity datasets. Deadenylation, decapping, and granule proteins are consistently near ZFP36L1 across conditions, while some contacts dip at 2 hours and recover by 5 to 16 hours. Mitochondrial ribosomal proteins become more proximal later. UPF1 and GIGYF1 show time-linked behavior and RNase sensitivity that fits roles in mRNA surveillance and translational control. These observations support a dynamic hub model, though they remain proximity-based rather than direct binding maps.

We thank the reviewer for their careful reading and thoughtful summary. Please find our point-to- point response below.

Major comments

1) The key conclusions are directionally convincing for a broad and dynamic ZFP36L1 neighborhood in human T cells. The data robustly recover established complexes and add plausible candidates. The time-course and RNase experiments strengthen the claim that interactions shift with activation state and RNA context. The functional tests around UPF1 and GIGYF1/2 point to biological relevance. That said, some statements could be qualified. The statement that ZFP36L1 "coordinates" multiple pathways implies mechanism and directionality that proximity data alone cannot prove. I suggest reframing as "positions ZFP36L1 within" or "supports a model where ZFP36L1 sits within" these networks.

We thank this reviewer for considering our data ‘directionally convincing, and robust, adding new plausible candidates as interactors with ZFP36L1’. We agree that the proposed wording is more appropriate and have changed it accordingly (**page 15)**.

3) The inference that ZFP36L1 proximity to decapping and deadenylation complexes reflects pathway engagement is reasonable and, frankly, expected. Still, where the manuscript moves from proximity to function, the narrative works best when supported by orthogonal validation. Two compact additions would raise confidence without opening new lines of work. First, a small set of reciprocal co-IPs for PATL1 or DDX6 at endogenous levels in activated T cells, run with and without RNase, would tie the RNase-class assignments to biochemistry. Second, a short-pulse proximity experiment using a reduced biotin dose and shorter labeling window in activated cells would address whether long incubations drive non-specific labeling. Both are feasible in 2 to 3 weeks with minimal extra cost for antibodies and MS runs if the facility is in-house.

We fully agree with the reviewer that orthogonal biochemical validation is valuable. Therefore, we had combined proximity labeling between 0-2h, 2-5h and 5-16h to obtain a time-resolved ZFP36L1 co-IP ± RNase, allowing us to address the dynamic behavior and potential temporal broadening of the interactome.
As to reciprocal co-IPs: we indeed had attempted to perform them. However, we could not find suitable antibodies for PATL1, which convincingly identified PATL1 even on Western blot (see below).

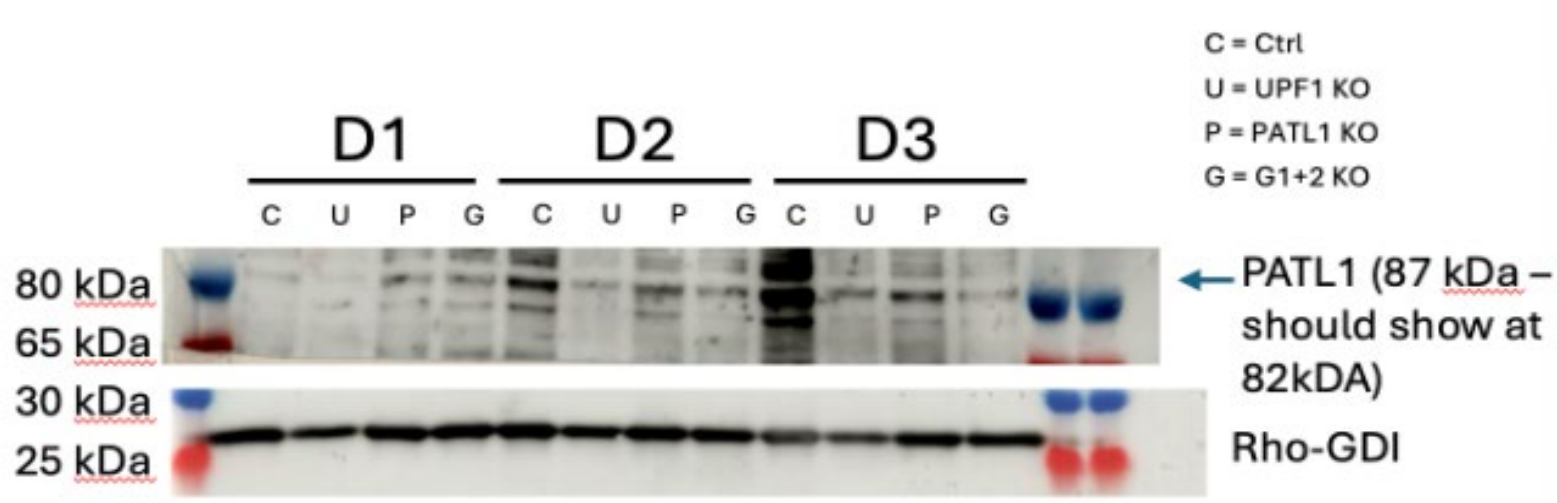

Westernblot analysis of Control gene-edited T cells (C), or edited for UPF1 KO (U), PATL1 KO (P), or GIGYF1+2 KO (G). T cells were stained for PATL1, and Rho-DGI as control. PATL1 should run at 87kDA. D1-3: indicates 3 donors.

As to DDX6, we now performed a reciprocal coIP (with clone #14632-1-AP, see data below). However, even though we effectively pulled down DDX6 from $1.5x10^6$ T cells, ZFP36L1 co-immunoprecipitation was not consistent throughout (see below). Also MS analysis of a native DDX6 co-IP did not display significant enrichment of ZFP36L1 (not shown). This could also point to fast and weak interactions..

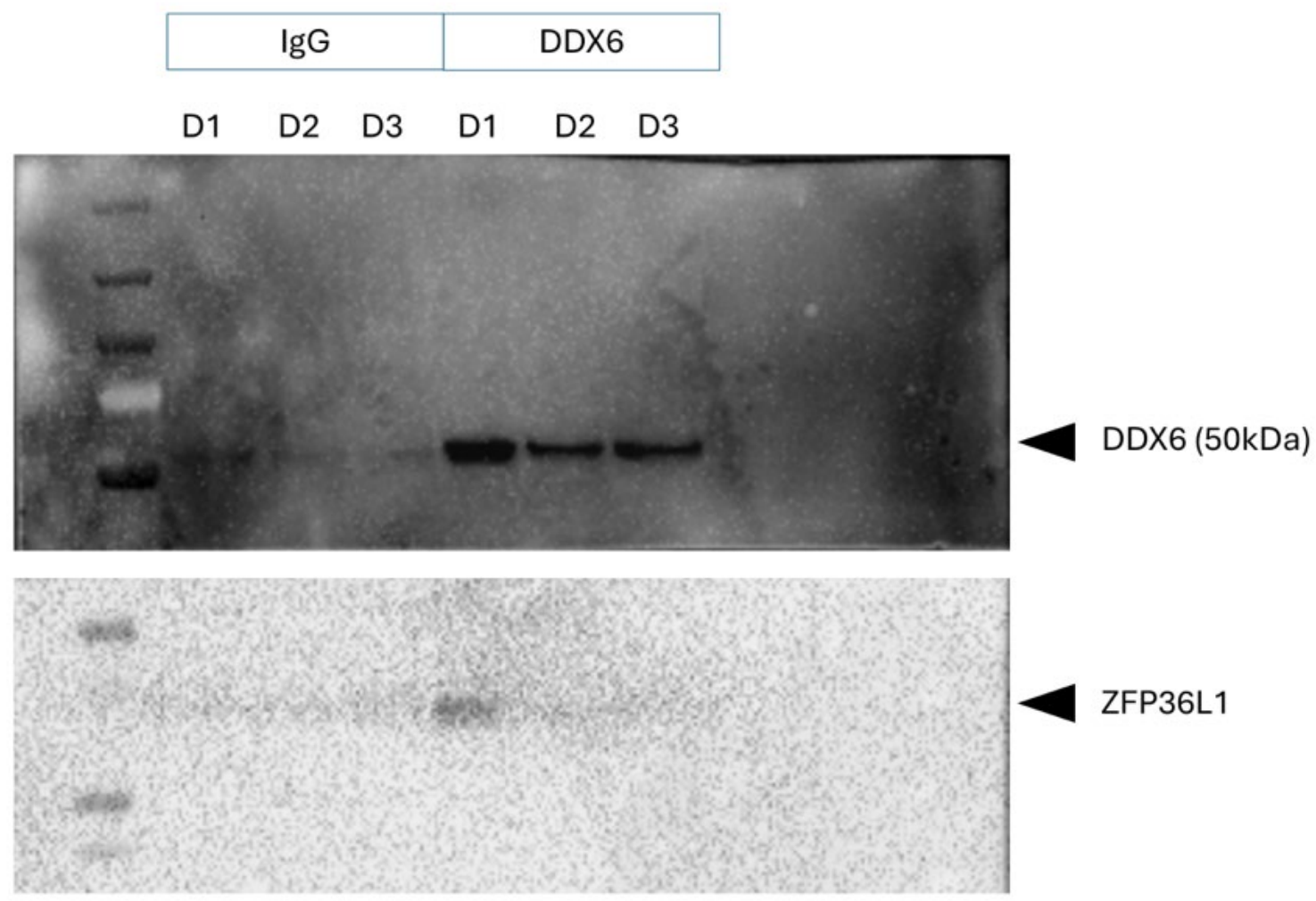

Co-Immunoprecipitation of DDX6 with ZFP36L1. 1.5×10^6 resting T cells. D1-3 indicates 3 individual donors.

We therefore adjusted our statement on DDX6 from the text (page 7), and indicate that DDX6 was identified with less confidence. Please be advised that DDX6 was already depicted in italic in Figure 3A of the original manuscript, i.e. its measurement was just below the threshold (a statement we failed to indicate, and we apologize).

To the other raised points:

1) Lowering the dose of biotin: As described in **Figure S1**, this appeared unsuccessful in primary human T cells, which is at least in part due to the reported dependence and increased use of biotin in primary T cells upon activation (Ref's 31-33 of this manuscript). This fact also prohibited T cell cultures in biotin-free medium prior to labeling, as most protocols do when working with cell lines.

2) Shorter labeling times: Please be advised that time of labeling was chosen based on the reported protein induction and activity on target mRNAs: A) ZFP36L1 expression peaks at 2h of T cell activation (Zandhuis, 2025; 0.1002/eji.202451641, Petkau, 2024; 10.1002/eji.202350700), B) time points when the strongest effects on T cell function are observed (between 4-5h), and when T cell responses enter the late phase of activity (5-16h; Popovic, 2023; 10.1016/j.celrep.2023.112419). We realize that additional explanation is warranted for this rationale, which we now provide on page 8).

4) Reproducibility is helped by donor pooling, repeated T-cell screens, Jurkat confirmation, and detailed methods including MaxQuant, LIMMA, and supervised patterning. Deposition of MS data is listed. The authors should consider adding a brief, stand-alone analysis notebook in SI or on GitHub with exact filtering thresholds and "shape" definitions, since the supervised profiles are central to claims. This would let others reproduce figures from raw tables with the same code and workflows.

We thank the reviewer for this suggestion. We uploaded our code to GitHub, and included the link in the methods section (page 24): https://github.com/ajhoogendijk/ZFP36L1_UltraID

5) Replication and statistics are mostly adequate for discovery proteomics. The thresholds are clear, and PCA and correlation frameworks are appropriate. For functional readouts in edited T cells, please make the number of donors and independent experiments explicit in figure legends, and indicate whether statistics are paired by donor. Where viability differs (UPF1), note any gating strategies used to avoid bias in puromycin or activation marker measurements. These clarifications are quick to add.

We now highlighted the requested information for each figure panel, rather than putting it at the bottom of the figure legends (which test used, donor numbers etc).

Minor comments

6) The UltraID optimization in primary T cells is useful, but the long 16-hour labeling and high biotin should be framed as a compromise rather than a standard. A short statement about potential off-target labeling during extended incubations would set expectations and justify the RNase and time-course controls.

Please be advised that as indicated above 1) high biotin was required because primary T cells depend on biotin and 2) increase biotin absorption a 2-7-fold upon activation (Ref 31-33 from the paper). For better time resolution, we included a labeling of 2h (from 0-2h of activation), 3h (from 2-5h) and 9h (from 5-16h) of T cell activation. Nevertheless, we agree that we cannot exclude the risk of off-target labeling, which in fact is inherent to any type of labeling and pulldown method. We now include such statement in the discussion, and highlight that further downstream validation experiments are warrented (page 16).

7) The overlap across T-cell screens and with HEK293T APEX datasets is discussed, but a compact quantitative reconciliation would help. A table that lists shared versus cell-type-specific interactors with brief notes on known expression patterns would make this point concrete.

We agree and we now included this table as a new tab in "**Supplemental Table _Figure 2 & Sup Fig 2" named "Overlap all data sets".**

8) Figures are generally clear. Where proximity and total proteome PCA are shown, consider adding sample-wise annotations for donor pools and activation time to help readers link variance to biology. Ensure all volcano plots and heatmaps display the exact cutoffs used in text.

We agree that sample-wise annotations would be a nice addition. However, when implementing it for e.g. **FIgure 1D&E,** we realized that due to the high numbers of variables including also sample-wise annotations made the panels very difficult to read. We therefore decided against it.

As to cut-offs of volcano plots**:** we have adjusted it (**Supplemental Figure 5)**.

9) Prior work on ZFP36 family roles in decay, deadenylation via CCR4-NOT, granules, and translational control is cited within the manuscript. In a few places, recent proximity and interactome papers could be more explicitly integrated when comparing overlap, especially where conclusions differ by cell type. A concise paragraph in Discussion that lays out what is truly new in primary T cells would help clarify the contribution of this work to the field.

We appreciate this suggestion. The comparison with the Bestenhorn data set is reported in Supplementary table 1, tab "Overlap" (colom C-F versus H). Indeed, we find differences between identified targets. For instance, UltraID identifies DCP1B, CNOT3, and CNOT8 (row 9, 11, 13 of the table), whereas APEX in HEK293 T/RAW cells does not. However, while co-identification between the two data sets may yield more comfort in calling a protein a ZFP36L1 interactor, we find it difficult to interpret the differences in identification. At this point, it is impossible to disentangle whether differences in identification are due to the different cell types, or due to the different methods employed (Ultra-ID versus APEX), or even due different culture conditions. Only a direct comparison with changing one parameter at a time would allow to disentangle the underlying reasons. However, we cannot employ APEX in T cells: when setting up the systems and deciding for the methods to be used, we learned that the addition of H_2O_2 (required for APEX labeling) to primary T cells results in immediate cell death, thus impeding the use of APEX in T cells. Whereas this technical limitation prohibits direct comparisons without deep time and financial investments, it underscores the valuable contribution of the UltraID-based approach we present here. We now indicated these considerations in the Discussion (page 16).

Reviewer #21 (Significance (Required)):
Nature and type of advance. **The study is a technical and contextual advance in mapping ZFP36L1 proximity partners directly in human primary T cells during activation. The combination of time-resolved labeling and RNase-class assignments is informative. The CRIS PR perturbations provide an initial functional bridge from proximity to phenotype, especially for UPF1.**
Context in the literature. **ZFP36 family proteins have long been linked to ARE-mediated decay, CCR4-NOT recruitment, and granule localization. The present work confirms those cores and extends them to include decapping and GIGYF1/2-4EHP scaffolds in primary T cells with temporal resolution. The UPF1 link to ZFP36L1 expression adds a plausible surveillance angle that merits follow-up. The cell-type specificity analysis versus HEK293T underscores that proximity networks vary with context.**
Audience**. Readers in RNA biology, T-cell biology, and proteomics will find the dataset valuable. Groups studying post-transcriptional regulation in immunity can use the resource to prioritize candidate nodes for mechanistic work.**

Expertise and scope. **I work on post-transcriptional regulation, RNA-protein complexes, and T-cell effector biology. I am comfortable evaluating the conceptual claims, experimental design, and statistical treatment. I am not a mass spectrometry specialist, so I rely on the presented parameters and deposited data for MS acquisition specifics.**

To conclude, the manuscript delivers a substantive proximity map of ZFP36L1 in human T cells, with useful temporal and RNA-class information. The UPF1 observations are promising and would benefit from a compact rescue to secure causality. A few minor additions for biochemical validation and transparency in replication would further strengthen the paper.

We thank the reviewer for this comprehensive and constructive assessment. We agree that our study primarily provides a substantive and well-annotated proximity map of ZFP36L1 in human T cells, including temporal and RNA-class information, and that the UPF1 observations constitute a promising lead that merits more detailed mechanistic analysis in follow-up studies.

Reviewer #2 (Evidence, reproducibility and clarity (Required)):

The manuscript by Wolkers and colleagues describes the protein interactome of the RNA-binding protein ZFP36L1 in primary human T-cells. There is inherent value in the use of primary cells of human origin, but there is also value in that the study is quite complete, as it is performed in a variety of conditions: T-cells that have been activated or not, at different time points after activation, and by two methods (co-IP and proximity labeling). One might imagine that this basically covers all what can be detected for this protein in T-cells. The authors report a large amount of new interactors involved at all steps in post-transcriptional regulation. In addition, the authors show that UPF1, a known interactor of ZFP36L1, actually binds to ZFP36L1 mRNA and enhances its levels. In sum, the work provides a valuable resource of ZFP36L1 interactors. Yet, how the data add to the mechanistic understanding of ZFP36L1 functions and/or regulation of ZFP36L1 remains unclear.

We thank the reviewer for this enthusiasm on our experimental setups, considering the use of primary T cells of inherent value and our study with the variety of conditions complete.

Major comments:

1) Fig 2: It is confusing that the Pearson correlation to define ZFP36L1 interactors is changed depending on figure panel. In panels A-C, a correlation {greater than or equal to} 0.6 is used, while panel D uses a correlation > 0.5, which changes the nº of interactors. Then, this is changed again in Fig 3A for some cell types but not for others. Why has this been done? It would be better to stick to the same thresholds throughout the manuscript.

Please be advised that different correlation thresholds arise from the composition of the individual datasets: they in depth, number of controls, and the overall dynamic range. The initial proximity labeling experiment (**Figure 2A–C**) had a higher depth and a larger number of suitable control samples, which allowed us to apply a stricter cutoff ($r \geq 0.6$). The time-course experiment and some of the cross-cell-type comparisons have fewer controls and somewhat lower depth, which then required a more permissive threshold (e.g. $r > 0.5$) to retain known core interactors.

We fully agree that this rationale needs to be explicit. In the revised manuscript we (i) clearly state for each dataset which correlation cutoff is used (ii) explain how these thresholds are set (methods section **page 24**).

2) Fig 3A: It would be nice to have the information of this Figure panel as a Table (protein name, molecular process(es), known or novel, previously detected in which cells) in addition to the figure.

We thank the reviewer for this suggestion. We decided to combine this information with the suggestion of Reviewer 1 and created a table that has detailed description of function of the interactors found in Figure 3 as well as overlap of all screen (including HEK screen by Bestehorn *et al*). We now provided this in **Supplemental Table 1 tab "Overlap all data sets"**.

3) Fig 6: To what extent are the effects of UPF1 and GIGFYF1 knock-out on translation and T-cell hyper-activation mediated by ZFP36L1? If deletion of ZFP36L1 itself has no effect on these processes, it seems unlikely that it is involved. In this respect, I am not sure that Fig 6 contributes to the understanding of ZFP36L.

We appreciate this conceptual question. In our dataset, ZFP36L1 knockout affects T-cell activation markers, but does not recapitulate the increased global translation observed upon UPF1 or GIGYF1/2 deletion. We now discuss this finding more explicitly in the Results and Discussion. We discuss the possibility that other ZFP36 family members (e.g. ZFP36/TTP, ZFP36L2) may partially compensate for the absence of ZFP36L1 in some readouts (Zandhuis et al. 2025; 0.1002/eji.202451641, Petkau et al. 2024; 10.1002/eji.202350700), Moreover, we emphasize that at this point it is not clear whether ZFP36L1's contribution to UPF1 and GIGYF1 protein levels is direct or indirect.

We nonetheless consider Fig. 6 an important component of the story, as it demonstrates that proximity partners emerging from the interactome (UPF1, GIGYF1/2) have measurable functional consequences on T cell activation and translational control, thereby illustrating how the resource can guide mechanistic hypotheses. We now more carefully phrase this as "first indications of mechanism" and avoid implying that these phenotypes are mediated exclusively via ZFP36L1.

4) Fig 7E: Differences in ZFP36L1 mRNA expression are claimed as a consequence of UPF1 deletion, and indeed there is a clear tendency to reduction of ZFP36L1 mRNA levels upon UPF1 KO. Yet the difference is statistically non-significant. Please, repeat this experiment to increase statistical significance. In addition, a clear discussion on how UPF1 -generally associated to mRNA degradation- contributes to increase ZFP36L1 mRNA levels would be appreciated.

We respectfully disagree with including repeats for increasing (or until meeting) statistical power. We would like to stress that trends at 0h with n=3 donors are similar to those for n=7 at 3h of activation (**Fig. 7E**). This is evident despite the big variation between individual samples, a feature we regularly observe when working with primary human material. However, it does not alter the overall biological conclusion, which is further strengthened by our findings upon 3h activation (Fig 7E). We will, however, include a point on how UPF1 possibly may contribute to the decreased ZFP36L1 mRNA levels, as suggested, on page 17).

5) Fig 6A: The decrease in global translation by GIGFYF1 knock-out upon activation claimed by the authors is not clear in Fig 6A and is non-significant upon quantification. Please modify narrative accordingly.

Indeed, this was not phrased well. We now adjusted our description to match the statistical analysis (**page 12)**.

6) Page 6: The authors state 'This included the PAN2/3 complex proteins which trim poly(A) tails prior to mRNA degradation through the CCR4/NOT complex'. To the best of my knowledge, the CCR4/NOT complex does not degrade the body of the mRNA. Both PAN2/3 and CCR4/NOT are deadenylases that function independently.

We thank the reviewer for highlighting this inaccuracy. PAN2/3 and CCR4–NOT are indeed both deadenylase complexes that function independently rather than one acting strictly upstream of the other in degrading the mRNA body. We have corrected this statement: PAN2/3 and CCR4–NOT cooperate in facilitating poly(A) tail shortening. They do not actively degrade the mRNA body, which is instead handled by the downstream decay machinery **(page 6).**

7) Please, label all Table sheets. Right now one has to guess what is being shown in most of them. Furthermore, it would be convenient to join all Tables related to the same Figure in one unique Excel with several sheets, rather than having many Tables with only one sheet each.

We appreciate this suggestion, and have implemented it, including fusing the tables.

Minor comments:

8) Fig 1E: Shouldn't there be a better separation by biotinylation in the UltraID IP principal component analysis? In theory, only biotinylated proteins should be immunoprecipitated.

In theory, this should be the case. However, in practice, pull down experiments always suffer from background stickiness of proteins to tubes, beads, specific properties of proteins, etc. Combined, these known background issues highlight how important it is to include appropriate control samples, allowing for unequivocal call of proteins above background.

Furthermore, and as already indicated in the manuscript on page 3, primary T cells depend on Biotin for survival/division, which prohibited us to culture T cells in biotin-free medium prior to labeling, as is employed for cell lines (it resulted in profound cell death even for short periods). Therefore, as biotin is continuously present, some of the 'background' biotinylation signal may in fact even already stem from low level proximity labeling. We now discuss this point on page 16.

9) Fig 3B-E: Is the labeling not swapped, top (always +) is Biotin and bottom (- or +) is aCD3/aCD28?

We thank the reviewer for catching this mistake- we have corrected it.

11) Fig S1A: Why is there so much labeling in the UltraID only lane without biotin?

This is a phenomenon also reported by others (Kubitz 2022; 10.1038/s42003-022-03604-5: **Figure 5A**). UltraID is a small protein (19.7KD), which a comparable size to TurboID variants (Kubitz 2022; 10.1038/s42003-022-03604-5). If not fused to a protein and thus not tethered to a specific compartment, these molecules can diffuse through the cytoplasm and seem to biotinylate any protein they 'bump' into. Please be advised that we included this control to test this in T cells, and to to substantiate as to why we use GFP-UltraID- as control rather than empty UltraID, to limit such background effects. We now better highlight this point in the results section (page 3/4).

12) Fig S1E: Please, explain better. What is WT?

We thank the reviewer for catching this inconsistency. We adjusted the figure, and changed WT to UT, i.e. untransduced T cells (Figure legends, page 34). We also improved the figure layout as well for clarity purposes.

13) Fig S4B: Please, explain the labels on top of the shapes.

We updated figure S4B, to better explain how labels above each shape are chosen (e.g. indicating specific clusters, functional categories, or experimental conditions, as appropriate). This should make the reading more intuitive.

14) Page 3: A time-course of incubation with biotin is lacking in Fig S1B, and thereby it is confusing that the authors direct readers to this figure when an increased to 16h incubation is claimed to be better.

Please be advised that short labeling times yielded no results in primary human T cells. We therefore performed all initial analyses with 16h biotinylation (**Figure S1B)**. Only after achieving good results (**Figure 1B**), we performed time course experiments (presented in **Figure 4,** lowering incubation times to 2h, 3h and 9h). We realize that this is confusing and we have rephrased our considerations on page 3.

Reviewer #2 (Significance (Required)):
Strengths: A thorough repository of ZFP36L1 interactors in primary human T-cells. A valuable resource for the community.
Weaknesses: There is little mechanistic insight on ZFP36L1 function or regulation.

We would like to highlight that the purpose of our study was to provide a comprehensive interactome analysis of ZFP36L1, and to study the dynamics of these interactions. In addition to known interactors, we identified novel putative interactors of ZFP36L1. We indeed did not follow up on all interactions, which we consider beyond the scope of this manuscript. Rather, we consider our study as a toolbox for the community. In Fig 6-7, we show first examples of such follow-up studies and how our findings help provide mechanistic insights on ZFP36L1 interactors, thereby exemplifying how the findings of this resource paper can be used by the community.

Reviewer #3 (Evidence, reproducibility and clarity (Required)):

The authors have analyzed the interactome of ZFP36L1 in primary human T cells using a biotin-based proximity labeling method. In addition to proteins that are known to interact with ZFP36L1, the authors defined a multitude of novel interactions involved in mRNA decapping, mRNA degradation pathways, translation repressors, stress granule/p-body formation, and other regulatory pathways. Time-lapse proximity labeling revealed that the ZFP36L1 interactome undergoes remodeling during T cell activation. Co-IP for ZFP36L1

executed in the presence/absence of RNA further revealed the interactome and possible regulators of ZFP36L1, including the helicase UPF1. In addition to interacting with ZFP36L1, UPF1 promotes the ZFP36L1 protein expression, seemingly by binding to the ZFP36L1 mRNA transcript, and in some way stabilizing it. This comprehensive interactome map highlights the widespread interactions of ZFP36L1 with proteins of many types, and its potential roles in diverse T cell processes. Although somewhat descriptive, rather than hypothesis-testing, this work represents an important contribution to understanding the potential roles of the ZFP36 family proteins, and sets up many future experiments which could test molecular details.

We thank the reviewer for these thoughtful points, and for recognizing our paper as an important contribution for the field as resource, that should support future experiments.

Major points:
1) Can the authors discuss the specificity of the antibody for ZFP36L1 used in the Co-IP experiments? The antibody listed in Appendix A is abcam catalog number ab42473, although the catalog number for this antibody (unlike the others major ones used) is not listed in the Methods section - please add this to the Methods to make it easier for readers to find this detail. Could this antibody also be immunoprecipitating ZFP36 or ZFP36L2? Other antibodies have had cross-reactivity for the different family members. It is also notable that this antibody has been discontinued by the manufacturer (https://www.abcam.com/en-us/products/unavailable/zfp36l1-antibody-ab42473). Have the authors tried the current abcam anti-ZFP36L1 antibody being sold, catalog number ab230507?

We apologize for not having clarified this important technical point. Catalog numbers of the anti-ZFP36L1 antibody used for co-IP are now included (ab42473, Abcam) to the Methods section, in addition to Appendix A. The antibody ab42473 has indeed been discontinued by the manufacturer. We have contacted the manufacturer on multiple occasions with no luck.

We tested multiple alternatives for anti-ZFP36L1 antibodies, including the Abcam antibody ab230507 (indicated as 'old'; see western blots below). In our hands, none represented a good alternative with either much weaker or less specific detection of ZFP36L1. In fact, only the antibody 1A3 recognized ZFP36L1, which we then used for the Co-IP. Importantly, irrespective of lower detection signal, the migration patterns resemble those of ab42473 in our co-IP experiments and match the expected molecular weight of ZFP36L1. Importantly, ZFP36 or ZFP36L2 are larger, and therefore this antibody does not seem to cross-react. To clarify, we now added the band sizes to the WB in **figure 7D**).

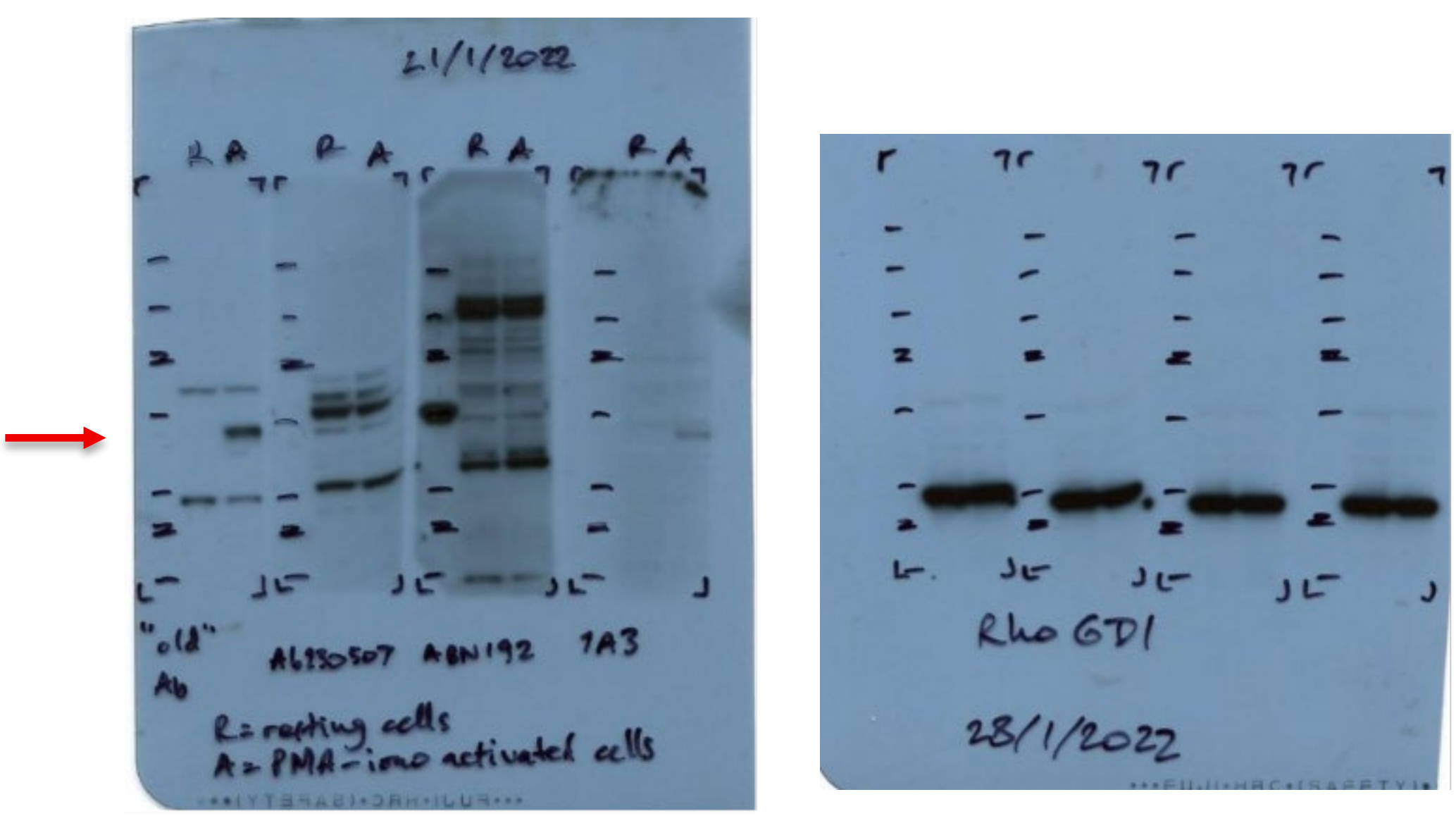

(Left blot): Test run of 4 different ZFP36L1 antibodies on T cells that were either resting (R) or activated for 4h with PMA/ionomycin activated (A). 1st 'Old':ab42473; 2nd: Ab230507; 3rd: ABN192; 4th: 1A3. (Right blot): matching Rho-GDI as loading control.

2) On this point, the authors report interactions between ZFP36L1 and its related proteins ZFP36 and ZFP36L2 in the Co-IP experiment (Supp 5C). Did these proteins interact in the proximity labeling? Ideally this could be discussed in the Discussion section.

ZFP36 and ZFP36L2 were indeed detected as co-precipitating with ZFP36L1 in the co-IP experiments but were not found as high-confidence interactors in the UltraID proximity labeling datasets (Supplemental Table 1 tabs IP_results & Shape correlations & Supplemental Table 5). Also in the APEX proximity labeling in RAW macrophage cells (Bestehorn, 10.1016/j.molcel.2025.01.001) ZFP36 or ZFP36L1 were not reported as ZFP36L1 interactors. We now explicitly discuss this finding on **page 17**.

3) Can the authors discuss more fully the limited overlap in identified interactors across the two proximity labeling screens performed in primary T cells (Fig 2C)? Likewise, can the authors comment on the very limited overlap between the screens in T cells and the published ZFP36L1-APEX proximity labelling experiment performed in the HEK293T cell line by Bestehorn et al. (ref 42)? Only 6.8% of proteins found in either T cell screen were found as interactors in this cell line. The authors comment that this may be because "...either expression of certain proteins is cell-type specific, or [because] ZFP36L1 has cell-type specific protein interactions, in addition to its core interactome". While I agree that cell-type specific interactions may be at play, I would think most of the interactors found in the T cell screens are widely expressed proteins necessary for central cell functions.

First, the apparent overlap percentage depends on depth and filtering. As noted above and now detailed in a new Supplementary table, a core set of decapping, deadenylation, and granule-associated factors is consistently recovered across our T-cell screens and the HEK293T APEX dataset. However, beyond this core protein, the overlap between data sets is reduced, reflecting several factors: (i) differences in expression levels of many interactors between HEK293T cells and primary T cells; (ii) the activation-dependent nature of ZFP36L1 function in T cells, which cannot be fully mimicked in HEK293T; (iii) different proximity labeling enzymes and fusion constructs (APEX vs UltraID, different tags, expression levels); and (iv) distinct experimental designs and control strategies, which influence statistical filtering and the effective “depth” of each interactome.

In the revised Discussion and in the new comparative table, we now emphasize that while many of the ZFP36L1 proximity partners identified in T cells are indeed widely expressed, their effective labeling and enrichment are strongly context dependent. We therefore interpret the relatively limited overlap as highlighting both a robust core interactome and substantial context-specific remodeling, rather than as evidence of artifacts in one or the other dataset.

Minor comments:

4) In Figure 3D, the legend states that black circles indicate significantly enriched proteins in biotin samples, while grey circles indicate non-significant enrichment. However, some genes, including DCP1A, DDX6, YBX1, have black circles in the -biotin group and grey in the +biotin group, which creates confusion in interpretation.

We thank the reviewer for this comment. We have accidentally switched the labeling of biotin and activation. We have now fixed it.

5) Did the authors find any interactors whose expression is known to be specific to CD4 or CD8 T cells?

In our current dataset we did not identify interactors whose presence was restricted to CD4 or CD8 T-cells. This T cell subset specificity for measuring differential ZFP36L1 interactomes represents an interesting avenue for future targeted studies, which we now indicated in the discussion (**page 16**).

Reviewer #3 (Significance (Required)):

The authors present the first comprehensive analysis of the ZFP36L1 interactome in primary T cells. The use of biotin-based proximity labeling enables detection of physiologically relevant interactions in live cells. This approach revealed many novel interactors. Strengths include the overall richness of the dataset, and the hypothesis-provoking experiments that could follow in the future. Limitations include somewhat limited overlap with a published proximity labeling dataset from performed in a different cell line, suggesting that there may be artifacts in one or both datasets. The audience for this article would include those interested broadly in RNA binding proteins and those interested in post-transcriptional and translational regulation. I have immunology expertise on T cell activation and differentiation and expertise on transcriptional and post-transcriptional regulation of gene expression in T cells.

4. Description of analyses that authors prefer not to carry out

Reviewer 1:

Major comments

2) UPF1, as an upstream regulator of ZFP36L1 expression, is a promising lead. The reduction of ZFP36L1 protein and mRNA in UPF1 knockout, the non-rescue by MG132, and the UPF1 RIP on ZFP36L1 mRNA together argue that UPF1 influences ZFP36L1 transcript output or processing. This claim would read stronger with one short rescue or perturbation that pins the mechanism. A compact test would be UPF1 re-expression in UPF1-deficient T cells with wild-type and helicase-dead alleles. This is realistic in primary T cells using mRNA electroporation or virus-based systems. Approximate time 2 to 3 weeks, including guide design check and expansion. Reagents and sequencing about 2 to 4k USD depending on donor numbers. This would help separate viability or stress effects from a direct role in ZFP36L1 mRNA handling.

We agree that a rescue experiment with wild-type and helicase-dead UPF1 in UPF1-deficient primary T cells would be interesting. Unfortunately, however, UPF1 knockout T cells are less viable and divide less (**Supp Figure 6B)**, making further manipulations such as re-expression by viral transduction technically impossible. We have clarified this limitation in the Discussion on **page 18**, and have more explicitly indicated that UPF1 promotes ZFP36L1 mRNA and protein expression, while acknowledging that the precise mechanistic contribution of UPF1 (e.g. to transcript processing, export, or surveillance) remain to be fully resolved.

10) Fig 7A data is from another paper, so I suggest to move this panel to Supplementary materials.

We respectfully disagree. We extracted the data from a previously published dataset, which is a widely accepted method and certainly regularly used for main figure panels. Our re-analysis from Bestenhorn *et al* 2025; (10.1016/j.molcel.2025.01.001) confirms that ZFP36L1 interacts with UPF1 and GIGYF1/2 in the RAW 264.7 macrophage cell line, which we consider an important consolidation of our findings (and thus material for a main figure). To emphasize that this table is a re-analysis of published data, we now added to the panel Fig 7a 'extracted from Bestenhorn *et al*

Reviewer 2:

Major comments:

1) Fig 2: It is confusing that the Pearson correlation to define ZFP36L1 interactors is changed depending on figure panel. In panels A-C, a correlation {greater than or equal to} 0.6 is used, while panel D uses a correlation > 0.5, which changes the nº of interactors. Then, this is changed again in Fig 3A for some cell types but not for others. Why has this been done? It would be better to stick to the same thresholds throughout the manuscript.

Please be advised that different correlation thresholds arise from the composition of individual datasets: they differ in number of identified proteins, number of samples, controls, and the overall dynamic range (which is inherent to experiments performed at different days and different MS runs). Because the initial proximity labeling experiment (**Figure 2A–C**) identified more proteins and contained more control samples, we could apply a stricter cutoff ($r \geq 0.6$). As to the time-course experiment, it contained fewer control samples relative to the measured samples and the protein identification was a bit lower. We therefore opted for a more permissive threshold (e.g. $r > 0.5$), which we based on the identification of known core interactors.

We fully agree that this rationale needs to be more explicit. In the revised manuscript we (i) clearly state for each dataset which correlation cutoff is used (ii) emphasize that these thresholds are somewhat arbitrary and should not be directly compared across experiments, and (iii) highlight that our key biological conclusions do not depend on the exact boundary chosen but rather on the consistent enrichment of core complexes and pathways across analyses.

July 22, 2026

RE: Life Science Alliance Manuscript #LSA-2026-03825-T

Dr. Monika C Wolkers
Sanquin
Hematopoiesis
Plesmanlaan 125
Amsterdam 1066CX
Netherlands

Dear Dr. Wolkers,

Thank you for submitting your revised manuscript entitled "Proximity labeling reveals ZFP36L1 as a central hub for post-transcriptional regulation networks in T cells". Your manuscript was sent back to Reviewers 1 and 3 as per our original offer on your ms. As you will note both reviewers have acknowledged your efforts in resolving their previous concerns. That said, both of them had some minor concerns that we agree must be resolved in your ms text.
-Whilst we do not need any new experimental data, we agree with Reviewer 1 that you must soften your claims or explicitly state related caveats. Our suggestion is that you (1) incorporate the text changes sought by this reviewer concerning UPF1, including in the abstract, and (2) explicitly discuss the long-16hour biotinylation as a potential caveat in your discussion.
-We concur with Reviewer 3 that you must provide accurate information on the anti-ZFP36L1 antibody used.
-In agreement with Reviewer 1, we strongly urge you to include the negative data (DDX6) provided in your response letter as a supplemental figure.

Overall, we would be happy to publish your paper in Life Science Alliance pending final revisions necessary to meet our formatting guidelines, and resolution of points outlined above.

MANUSCRIPT ORGANISATION AND FORMATTING:

To avoid unnecessary delays in the acceptance and publication of your paper, please read the following information carefully. Full guidelines are available on our Instructions for Authors page, https://www.life-science-alliance.org/authors

Submission of a paper that does not conform to Life Science Alliance guidelines will delay the acceptance of your manuscript.
AWMR points:
-In figure 7B, the lanes corresponding to G1+2 (Resting) for the blots (UPF1 and ZFP36L1) appear as if they arise from different blots when compared to corresponding lanes (Ctrl and UPF1). If this is the case, please indicate with a clearer separation or a black line delineating the boundary between the gels.
-Please remove the claim on single deletions of GIGYF1 and GIGYF2 (line 344) or provide the appropriate evidence as per LSA's guidelines on 'data not shown'.
-Please identify the committee approving the experiments with PBMCs from human subjects (Line 514).
-Kindly re-label the tables in appendix 1 and appendix 2 as Table 1 and Table 2 in the main ms and accordingly callout to them in the materials and methods section.
-Please remove the phrase, 'sequence upon request', line 547 and provide the corresponding information. Please also include the GitHub link for MS data analyses scripts in your statement on data and code availability. Please note that LSA discourages making data available upon request rather than having it publicly available and we suggest that you modify your existing statement in alignment with our guidelines.
-Please provide concentrations for all antibodies in the provided table (page 27).
-Please include primer sequence for RPS18 in table on page 30.
-Please add a Running Title and a Summary Blurb / Alternate Abstract in our system.
-Please consult with your secondary corresponding author if they connected their account with their Orcid ID -- They should have received instructions on how to do so.
-Please add a Category for your manuscript in our system.
-Please add the X and Bluesky handles of your host institute/organization, as well as your own, and/or one of the authors, in our system.
-Please add your main, supplementary figure, and table legends to the main manuscript text after the references section.
-Please add Author Contributions to our system as well.
-Please rename "COMPETING INTERESTS" to "Conflict of Interest".
-Please add a callout for Figure S4D to your main manuscript text.
-Please be sure that the authorship listing and order is correct.

We encourage our authors to provide original source data, particularly uncropped/-processed electrophoretic blots and spreadsheets for the main figures of the manuscript. If you would like to add source data, we would welcome one PDF/Excel-file per figure for this information. These files will be linked as supplementary "Source Data" files.

It is Life Science Alliance policy that if requested, original data images must be made available to the editors. Failure to provide original images upon request will result in unavoidable delays in publication. Please ensure that you have access to all original data images prior to final submission.

If you are planning a press release on your work, please inform us immediately to allow informing our production team and scheduling a release date.

We welcome submissions of potential cover images for the issue of LSA in which your work would appear. If you have high quality images associated with this work, please feel free to email these, with a caption, to the journal office.

LSA encourages authors to provide a 30-60 second video where the study is briefly explained. These videos will be appear embedded with the manuscript online at Life Science Alliance, and on social media to promote the published paper and authors (for examples, see https://docs.google.com/document/d/1-UWCfbE4pGcDdcgzcmiuJl2XMBJnxKYeqRvLLrLSo8s/edit?usp=sharing). Corresponding or first-authors are welcome to submit the video. Please submit only one video per manuscript. The video can be emailed to contact@life-science-alliance.org

FINAL FILES:

To upload the final version of your manuscript, please log in to your account: https://lsa.msubmit.net/cgi-bin/main.plex
You will be guided to complete the submission of your revised manuscript and to fill in all necessary information. Please get in touch in case you do not know or remember your login name.

The following items are required for acceptance.

-- An editable version of the final text (.DOC or .DOCX) is needed for copyediting (no PDFs).

-- High-resolution figure, supplementary figure and video files uploaded as individual files: See our detailed guidelines for preparing your production-ready images, https://www.life-science-alliance.org/authors

-- Summary blurb (enter in submission system): A short text summarizing in a single sentence the study (max. 200 characters including spaces). This text is used in conjunction with the titles of papers, hence should be informative and complementary to the title. It should describe the context and significance of the findings for a general readership; it should be written in the present tense and refer to the work in the third person. Author names should not be mentioned.

The license to publish form must be signed before your manuscript can be sent to production. A link to the license to publish form will be available to the corresponding author only. Please take a moment to check your funder requirements.

Reviews, decision letters, and point-by-point responses associated with peer-review at Life Science Alliance will be published, alongside the manuscript. If you do want to opt out of having the reviewer reports and your point-by-point responses displayed, please let us know immediately.

Thank you for your attention to these final processing requirements. Please revise and format the manuscript and upload materials as soon as you are able.

Thank you for this interesting contribution to the literature. We look forward to publishing your paper in Life Science Alliance.

Sincerely,

Sarita Hebbar, PhD
Scientific Editor
Life Science Alliance
http://www.lsajournal.org

Reviewer #1 (Comments to the Authors (Required)):

I'm largely satisfied with the revision, which reads well as a resource, and most of my points are resolved. Two things still nag, both around the the UPF1 story. First, dropping the rescue rests on viral transduction being unworkable in poorly dividing knockout cells, but my original suggestion was mRNA electroporation precisely because it needs neither proliferation nor

selection; a transient WT versus helicase-dead experiement, read out early before viability diverges, would actually pin down whether helicase activity matters for ZFP36L1 mRNA and protein. As it stands the directionality (an NMD-associated helicase supporting a target's own transcript) is counterintuitive and still correlative, so I'd ask the authors either to attempt the electroporation or, failing that, to engage that route directly rather than only the viral one, and to soften the mechanistic wording accordingly. Second, the honest attempt at reciprocal co-IPs is appreciated, but the negative DDX6 result and the +/- RNase comparison should still appear in the supplement, since the negative was precisely what I asked for, with the RNase-class calls flagged as provisional. The short-pulse control is likewise argued around rather than done, which is worth a line alongside the new caveat. None of this is fatal and I'm supportive of publication; if the rescue isn't done I defer to the editor on whether this suffices, though the claim should then be qualified. Minor- the GitHub link needs checking and "Bestehorn" is spelt several different ways.

Reviewer #3 (Comments to the Authors (Required)):

The authors have acceptably answered the questions I brought up in my major and minor points. I have one question remaining about the identity of the antibody used for the co-IP experiments of ZFP36L1. In the Methods section and Appendix A, the authors now list catalog number ABN192 from Sigma-Aldrich (rabbit anti-ZFP36L1, polyclonal) as the antibody used for co-IP experiments. But in their Revision Plan document they state the following:

"We tested multiple alternatives for anti-ZFP36L1 antibodies, including the Abcam antibody ab230507 (indicated as 'old'; see western blots below). In our hands, none represented a good alternative with either much weaker or less specific detection of ZFP36L1. In fact, only the antibody 1A3 recognized ZFP36L1, which we then used for the Co-IP. Importantly, irrespective of lower detection signal, the migration patterns resemble those of ab42473 in our co-IP experiments and match the expected molecular weight of ZFP36L1. Importantly, ZFP36 or ZFP36L2 are larger, and therefore this antibody does not seem to cross-react. To clarify, we now added the band sizes to the WB in figure 7D)."

This paragraph in the Revision Plan seems to indicate that the antibody 1A3 (Sigma Aldrich Catalog number WH0000677M2, Monoclonal Anti-ZFP36L1 antibody produced in mouse, clone 1A3) was used for the co-IP experiments. Also, in the Western Blot provided in the Revision Plan, it appears that "ABN192" does not specifically immunoblot ZFP36L1, while "1A3" does, although weakly. Is the listing of ABN192 in the Methods and Appendix A a mistake, when instead Catalog number WH0000677M2 (Monoclonal Anti-ZFP36L1 antibody produced in mouse, clone 1A3) should have been listed?

August 25, 2026

RE: Life Science Alliance Manuscript #LSA-2026-03825-TR

Dr. Monika C Wolkers
Sanquin
Hematopoiesis
Plesmanlaan 125
Amsterdam 1066CX
Netherlands

Dear Dr. Wolkers,

Thank you for submitting your Research Article entitled "Proximity labeling puts ZFP36L1 as central hub for posttranscriptional regulation networks in Tcells". It is a pleasure to let you know that your manuscript is now accepted for publication in Life Science Alliance. Congratulations on this interesting work.

The final published version of your manuscript will be deposited by us to PubMed Central upon publication.

Your manuscript will now progress through copyediting and proofing. It is journal policy that authors provide original data upon request.

Your article will publish open access upon publication under a CC-BY license.

Reviews, decision letters, and point-by-point responses associated with peer-review at Life Science Alliance will be published, alongside the manuscript. If you do want to opt out of having the reviewer reports and your point-by-point responses displayed, please let us know immediately.

IMPORTANT: If you will be unreachable at any time, please provide us with the email address of an alternate author. Failure to respond to routine queries may lead to unavoidable delays in publication.

Scheduling details will be available from our production department. You will receive proofs shortly before the publication date. Only essential corrections can be made at the proof stage so if there are any minor final changes you wish to make to the manuscript, please let the journal office know now.

DISTRIBUTION OF MATERIALS:
Authors are required to distribute freely any materials used in experiments published in Life Science Alliance. Authors are encouraged to deposit materials used in their studies to the appropriate repositories for distribution to researchers.

You can contact the journal office with any questions, contact@life-science-alliance.org

Again, congratulations on a very nice paper. I hope you found the review process to be constructive and are pleased with how the manuscript was handled editorially. We look forward to future exciting submissions from your lab.

Sincerely,

Sarita Hebbar, PhD
Scientific Editor
Life Science Alliance
http://www.lsajournal.org
